# Global burden of cancer in adolescents and young adults aged 10–24 years: a trend analysis

**DOI:** 10.3389/fonc.2025.1663523

**Published:** 2025-11-03

**Authors:** Junjun Yang, Guoping Luo, Qin Xiang, Shuangliang Li, Yuan Feng

**Affiliations:** ^1^ Department of Infectious Diseases, Beijing Anzhen Nanchong Hospital of Capital Medical University & Nanchong Central Hospital, Nanchong, China; ^2^ Department of Hepatobiliary Pancreatic and Spleen Surgery, Beijing Anzhen Nanchong Hospital of Capital Medical University & Nanchong Central Hospital, Nanchong, China

**Keywords:** global burden of disease, cancer, adolescents and young adults, sociodemographic index, trend analysis

## Abstract

**Background:**

Cancer is an important cause of human death. We aimed to analyze the cancer burden in adolescents and young adults aged 10–24 years at global, regional, and national levels from 1990 to 2021.

**Methods:**

We analyzed global burden of disease (GBD) data from 1990 to 2021 to assess the cancer-related incidence, prevalence, death, disability-adjusted life-years (DALYs), and the corresponding age-standardized rates (ASRs) in adolescents and young adults aged 10–24 years by region, country, sociodemographic index(SDI), etiology and gender stratification. In addition, we evaluated health inequities caused by cancer burden from 1990 to 2021 and used bayesian age-period-cohort (BAPC) model to assess trend of the total cancer burden in adolescents and young adults aged 10–24 years.

**Results:**

In 2021, there were 235, 249.05(95%UI, 21, 7211.16 to 251, 1070.1) new cancer cases in adolescents and young adults aged 10–24 years and 94856.02 (95%UI: 85970.2 to 102769.63) deaths worldwide. In addition, there were 13499, 913.04 (95%UI, 1252506.95 to 1442998.32) prevalent cases and 6, 918, 657.72 (95%UI: 6, 254, 353.93 to 7, 480202.6) DALYs. Over the past 30 years, the age-standardized incidence rate (ASIR) and the age-standardized prevalence rate (ASPR) of total cancer in adolescents and young adults aged 10–24 years have increased globally, with the most significant increases in regions with high SDI, such as North America and parts of Europe and Asia. The age-standardized death rate(ASDR) and age-standardized DALY rate of total cancer have decreased significantly globally in adolescents and young adults aged 10–24 years. The ASDR and age-standardized DALY rate of total cancer in adolescents and young adults aged 10–24 years were highest in countries with lower SDI, particularly in South America and Africa. Among all regions, Tokelau, Niue, and Afghanistan had the highest ASDR in 2021. Among all cancers, leukemia, brain cancers and malignant neoplasm of bone & articular cartilage were the most common causes of cancer death in adolescents and young adults aged 10–24 years in 2021.

**Conclusions:**

Globally, the total cancer burden of adolescents and young adults aged 10–24 years have increased significantly over the past 30 years. Differences in adolescent and young adult cancer burden were evident across regions with different SDI levels. Developing effective strategies to reduce the total cancer burden of adolescents and young adults was critical to promoting global equity and population health.

## Introduction

Cancer is the leading cause of death in all age groups worldwide (1). Reports indicated that cancers posed a non-negligible burden in people aged 10–24 years (2, 3). Although tremendous advances in tumor diagnosis and treatment have improved survival time in adolescents and young adults aged 10–24 years, this survival rate varies widely across countries and regions (4–6). The health problems of adolescents and young adults have not received enough attention, and a comprehensive understanding of the tumor burden in adolescents and young adults is lacking in many countries and regions.

Currently, the vast majority of adolescents and young adults aged 10–24 years, an important constituent group of the human population, live in low- and middle-income countries (5). Adolescents and young adult cancer patients are often overlooked when estimating the global cancer burden, despite the fact that they are a distinct subgroup with unique epidemiological characteristics. The comprehensive estimates of the global cancer burden among adolescents and young adults aged 10–24 years are still lacking.

The Global Burden of Disease (GBD) is the world’s largest descriptive epidemiological study focusing on the burden of various diseases and injuries and incorporating associated risk factors and relative harms (7). Therefore, this study used the most recent data from GBD 2021 to provide insights into the global cancer burden of adolescents and young adults aged 10–24 years. By utilizing the extensive data and methodology of GBD 2021, we assessed cancer-related incidence, prevalence, mortality, disability-adjusted life years (DALYs) and the corresponding age-standardized rates (ASRs) in adolescent and young adults aged 10–24 years at the global, regional and national levels, and identified trends and patterns to effectively inform evidence-based prevention and management strategies.

## Methods

### Data source

Global adolescent and young adult (10–24 years) cancer burden data were obtained from GBD 2021. GBD 2021 provided an updated comprehensive analysis of 328 diseases and their 87 risk factors in 204 countries and territories worldwide around the world (8). In this study, GBD 2021 was used to obtain adolescent and young adult cancer incidence, prevalence, death, and DALYs from 1990 to 2021. Subgroup analyses were performed according to gender, age, region, and country. This study was used by the Institute for Health Metrics and Evaluation for the maintenance of the global health data exchange (GHDx) query tool (http://ghdx.healthdata.org/gbd-results-tool) to get the data. The tool allowed users to access annual frequencies, age-standardized incidence rate (ASIR), age-standardized prevalence rate(ASPR), age-standardized death rate(ASDR), age-standardized DALYs rate, and other data by gender, age, region, and country (9). By utilizing adolescent and young adult tumor data from the GBD database, the study was able to analyze trends and patterns in adolescent and young adult total cancer burden across different demographic and geographic factors, providing valuable insights into the global epidemiology of adolescents and young adults with cancer in specific periods. The study was reported in line with the STROCSS criteria (10).

### Estimation of total cancer burden in adolescents and young adults aged 10–24 years

GBD 2021 classified all tumors into 34 groups. Descriptive analyses were used to assess the cancer burden of adolescents and young adults at global, regional, and national levels. All countries and regions were divided into 27 regions based on epidemiologic similarity and geographic proximity. The 27 regions were grouped using the sociodemographic index (SDI) based on total fertility, mean educational attainment, and per capita income, including low SDI (<0.466), low-moderate SDI (0.466-0.619), intermediate SDI (0.619-0.712), medium-high SDI (0.712-0.810) and high SDI (≧̸0.810) (11). In this study, a total of 34 groups of adolescent malignancies were included, of which 33 were classified malignancies and 1 was unclassified malignancies (other malignancies). More details about the burden estimates were given in **Supplementary Table 1**. We used disease maps to visualize the overall ASIR, ASPR, ASDR, and age-standardized DALYs rate per 100, 000 people at the national level. We also assessed differences in cancer burden among adolescents and young adults by gender and age. In addition, we used *Joinpoint* regression analyses to assess global trends of cancer burden in adolescents and young adults aged 10–24 years and conducted a decomposition analysis of total cancer burden (12).

### Measuring health inequities

Spearman correlation analysis was used to analyze the correlation between total cancer ASIR, ASPR, ASDR, age-standardized DALYs rate, and SDI level in adolescents and young adults aged 10–24 years. In addition, the ASDR of adolescent and young adult total cancer patients was analyzed for health inequality. According to World Health Organization (WHO) recommendations, the slope index (SII) and concentration index (CII) were used to measure absolute and relative health inequalities between countries (13). SII was calculated by regression of ASDR for countries ranked by income, defined as the midpoint of the range of cumulative categories for countries ranked by GDP per capita. The least square method was used to adjust the heteroscedasticity. Based on the cumulative relative distribution of GDP per capita and the corresponding national burden, the concentration index was calculated as twice the area between the 45° diagonal (equality line) and the Lorentz curve. If the Lorentz curve was above the equality line, the health burden was concentrated in low-income countries and was expressed as a negative concentration index. Relevant studies showed that an absolute CII of 0.2-0.3 represented a fairly high level of relative inequality (14).

### Projection analysis

The BAPC modelling was used to forecast cancer incidence, prevalence, DALYs, deaths and their ASRs among adolescents and young adults (10–24 years) from 2022 to 2050. The BAPC framework is a log-linear Poisson model that assumes multiplicative age, period and cohort effects, each following a Poisson distribution with an appropriate link function. In the BAPC model, we implemented a second-order random walk prior for the age, period, and cohort effects, consistent with recommendations in previous literature and the standard implementation in the BAPC R package. This choice of prior assumes that changes in these effects are smooth across adjacent intervals, which is crucial for stabilizing long-term projections and mitigating overfitting. We acknowledge that this specific prior specification, while standard, does inherently influence the smoothness of the estimated trends. Therefore, alternative prior specifications could potentially lead to variations in the final estimates and projections. Baseline estimates for 2021 served as the starting point; a +1% annual percentage change was explored as a pessimistic scenario and -1% as an optimistic scenario. All projections were implemented with the R package BAPC (version 0.0.36) (15).

Despite its strengths in capturing temporal dynamics and age-cohort interactions, the BAPC model also has limitations. It assumes that past trends will continue smoothly into the future, which may not hold in the presence of abrupt epidemiological transitions, major public health interventions, or substantial changes in healthcare accessibility. The model also does not explicitly incorporate external risk determinants, such as environmental exposures, behavioral shifts, or emerging therapeutic innovations, which could substantially alter cancer trajectories. Furthermore, uncertainties in input data-particularly in regions with incomplete cancer registration-may propagate through the model and influence the precision of long-term forecasts. Therefore, while the BAPC model provides valuable insights into directional trends, its outputs should be interpreted as probabilistic projections rather than deterministic predictions.

### Statistical analysis

We analyzed the total cancer burden in adolescents and young adults aged 10–24 years by age, gender, year, and region. The study provided incidence, prevalence, death, and DALYs estimates, each accompanied by a 95% uncertainty interval (UI), indicating the potential variability in the findings (16). These intervals were determined by identifying scores of 2.5 and 97.5 for all 1, 000 samples in the post-validation distribution. We calculated the annual percentage change (APC) and its 95% confidence interval (CI), as well as the average annual percentage change (AAPC) and its 95%CI. The world map was developed using the ggplot2 package (version 3.4.2). All statistical analysis software included R(version 4.3), Joinpoint (version 4.9.0.0), and Stata MP(version 14.0). P <0.05 was considered statistically significant.

## Results

### Global cancer burden in adolescents and young adults aged 10–24 years

From 1990 to 2021, the number of new cancer cases increased from 187, 467 (174, 955.6 to 197, 932.54) to 235, 249.05 (95% UI: 217, 211.16 to 251, 070.1) among adolescents and young adults aged 10–24 years. During this period, the number of deaths decreased globally from 105, 662.59 (95, 297.08 to 113, 611.51) to 94, 856.02 (95% UI: 85, 970.2 to 102, 769.63). Additionally, the prevalence of cancer grew from 891, 522.37 (845, 124.39 to 930, 115.75) to 1, 349, 913.04 (95% UI: 1, 252, 506.95 to 1, 442, 998.32) cases, while DALYs decreased from 7, 689, 222.5 (6, 935, 362.88 to 8, 261, 872.84) to 6, 918, 657.72 (95% UI: 6, 254, 353.93 to 7, 480, 202.6). Over the past 30 years, the ASIR and ASPR of total cancer among adolescents and young adults aged 10–24 years have increased globally, with average annual percentage changes (AAPC) and 95% confidence intervals (CI) of 0.06 (0.02 to 0.09) (P<0.001) and 0.76 (0.66 to 0.85) (P<0.001), respectively. However, the ASDR and DALY rate have significantly decreased, with AAPC values of -1.07 (-1.12 to -1.02) (P<0.001) and -1.06 (-1.11 to -1.01) (P<0.001), respectively. The results of epidemiological trends of total cancer in 204 countries and regions of the world for four different measures, which were shown in **Supplementary Tables 1-5**.

### Regional and national cancer burden in adolescents and young adults aged 10–24 years

**Figure 1:**
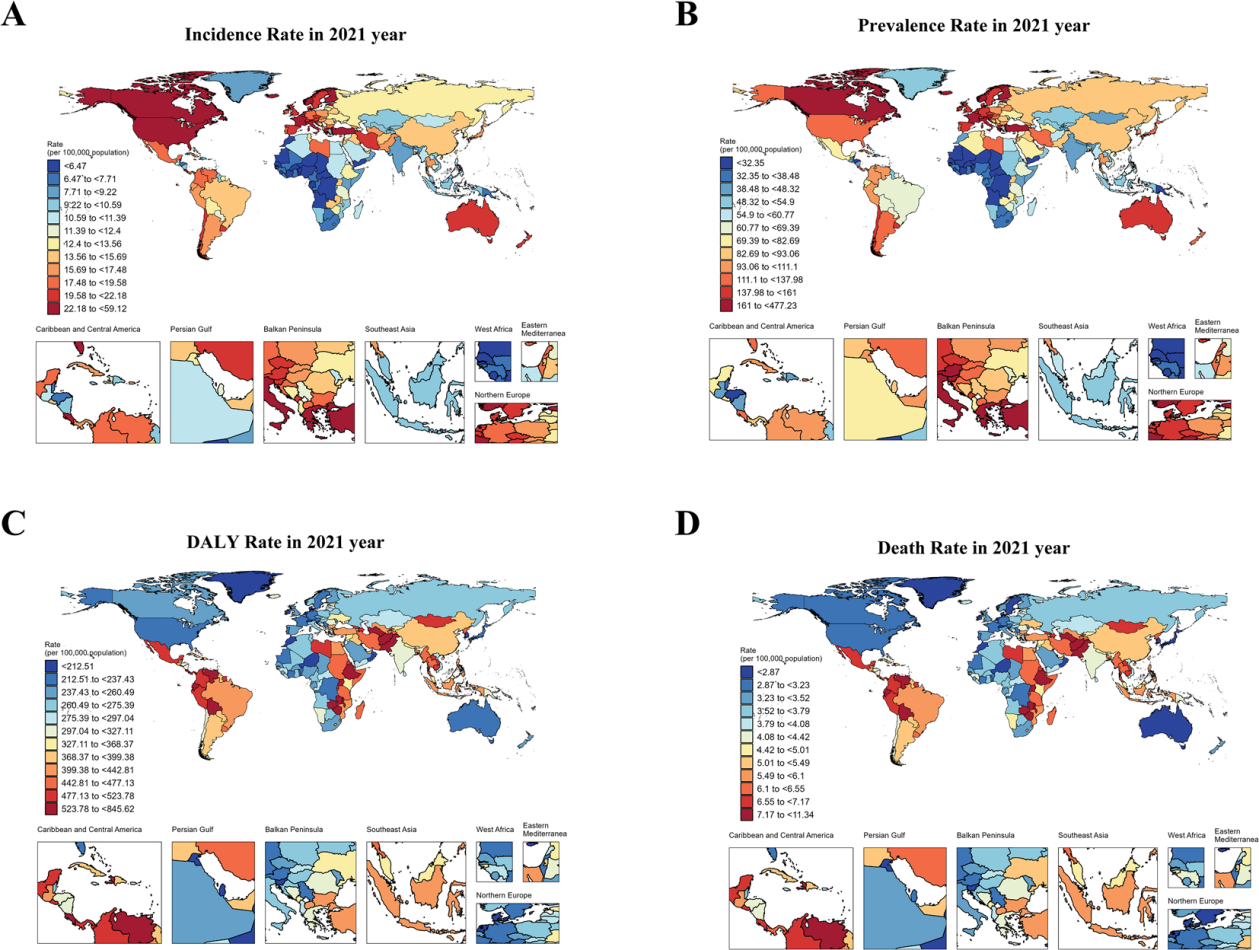
The ASIR, ASPR, ASDR and age-standardized DALY rate of cancers in adolescents and young adults aged 10–24 years in 2021. **(A)** showed ASIR of total cancer in adolescents and young adults aged 10–24 years in 2021. **(B)** showed ASPR of total cancer in adolescents and young adults aged 10–24 years in 2021. **(C)** showed age-standardized DALYs rate of total cancer in adolescents and young adults aged 10–24 years in 2021. **(D)** showed ASDR of total cancer in in adolescents and young adults aged 10–24 years in 2021.

**Figure 2:**
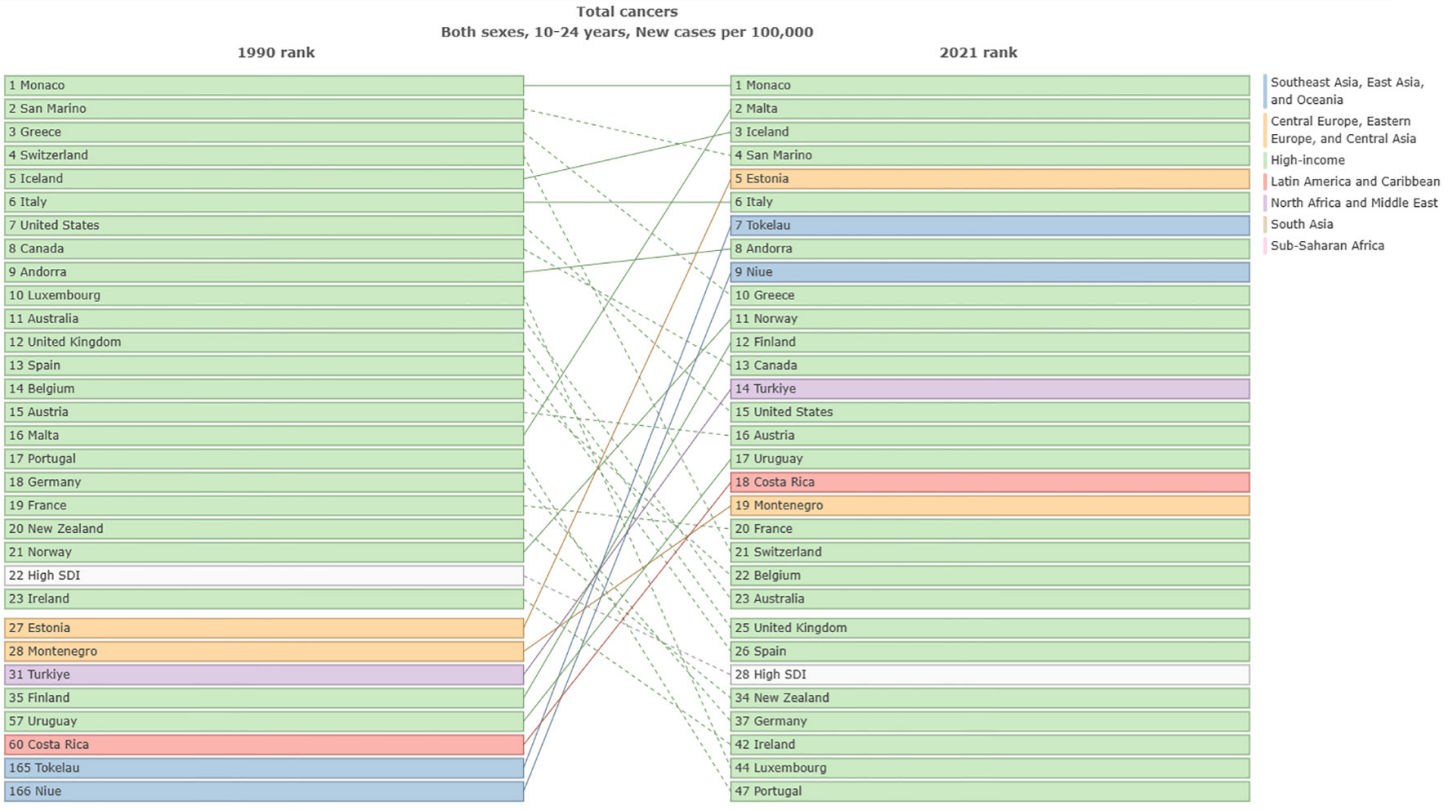
Comparison of ASIR of total cancers among adolescents and young adults aged 10–24 years in different countries from1990 to 2021.

In 1990, the highest ASIR of total cancer among adolescents and young adults aged 10–24 years was observed in North America, Australia, and parts of Europe, while ASDR of total cancer was primarily concentrated in regions such as Greenland, China, and Kazakhstan. In 2021, the highest ASIR of cancer in adolescents and young adults aged 10–24 years was located in North America, Europe, and Asia, and ASDR of total cancer was mainly concentrated in South America and Africa in 2021. Detailed results can be seen in **Figure 1**; **Supplementary figure 0**. In 1990, the top three countries with the highest ASIR of cancer in the world were Monaco [46.59, 95%UI (34.96 to 60.5)], San Marino [37.14, 95%UI (30.84 to 45.49)] and Greece [31.78, 95%UI (29.34 to 35)]. In 2021, the top three countries in the world were Monaco [61.99, 95%UI (47.25 to 83.75)], Malta [42.08, 95%UI (34.75 to 50.94)], and Iceland [36.73, 95%UI (31.83 to 42.15)] (**Figure 2**). In 1990, the top three countries with the highest ASPR of cancer in the world were Monaco [357.67, 95%UI (265.09 to 474.94)] and San Marino [279.7, 95%UI (230.94 to 347.28)] and Greece [279.7, 95%UI (230.94 to 347.28)]. In 2021, the top three countries were Monaco [501.04, 95%UI (379.78 to 691.03)], Malta [335.84, 95%UI (272.9 to 411.84)] and Iceland [295.16, 95%UI (252.13 to 342.52)] (**Supplementary Figure 1**). In 1990, the top three countries with the highest age-standardized DALYs rate in the world were Ethiopia [912.93, 95%UI (687.06 to 1143.93)], Turkiye[808.32, 95%UI(644.77 to 976.6)] and Afghanistan[776.68, 95%UI(492.56 to 1081.85)]. In 2021, it was Tokelau [855.56, 95%UI (646.93 to 1075.22)], Niue [760.5, 95%UI (620.92 to 905.59)] and Afghanistan [722.15, 95%UI (515.87 to 920.74)] (**Supplementary Figure 2**). In 1990, the top three countries with the highest ASDR in the world were Ethiopia [12.47, 95%UI(9.4 to 15.59)], Turkiye[11.09, 95%UI(8.84 to 13.4)] and Afghanistan[10.65, 95%UI(6.74 to 14.83), In 2021, it was Tokelau[11.43, 95%UI(8.7 to 14.28)], Niue[10.16, 95%UI(8.31 to 12.02)] and Afghanistan[9.95, 95%UI(7.04 to 12.69)] (**Supplementary Figure 3**). It can be seen that the cancer ASIR and ASPR of adolescents and young adults aged 10–24 years in high SDI areas were significantly higher than those in low SDI areas. However, the ASDR and age-standardized DALYs rate in low SDI areas were significantly higher than in high SDI areas. In addition, we compared the incidence, prevalence, DALYs and deaths in 26 regions from 1990 to 2021. Detailed results can be found in **Supplementary Figures 4-7**.

### Different cancers burdens in adolescents and young adults aged 10–24 years

**Figure 3:**
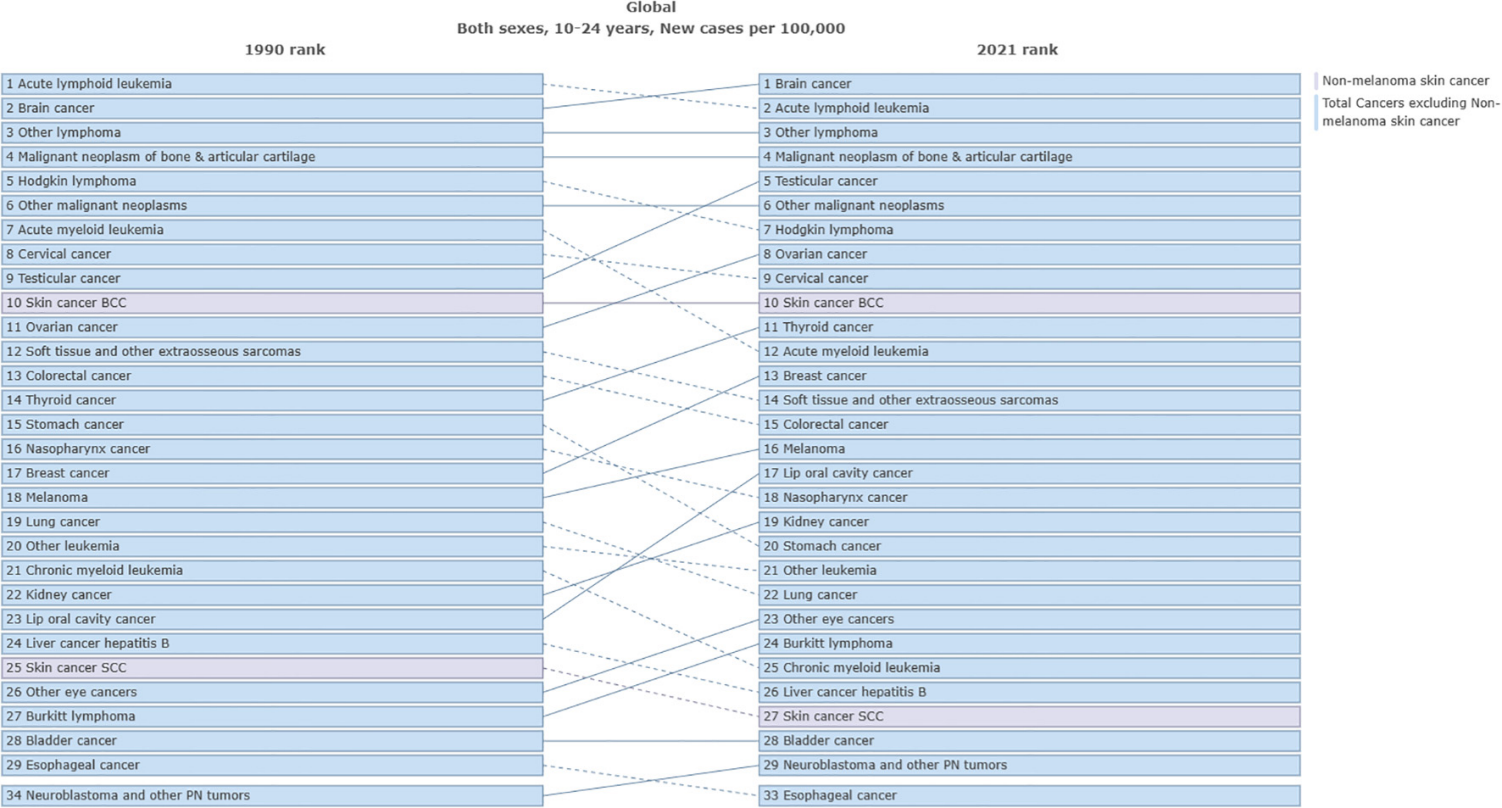
Comparison of the ASIR of different cancers among adolescents and young adults aged 10–24 years from 1990 to 2021.

**Figure 4:**
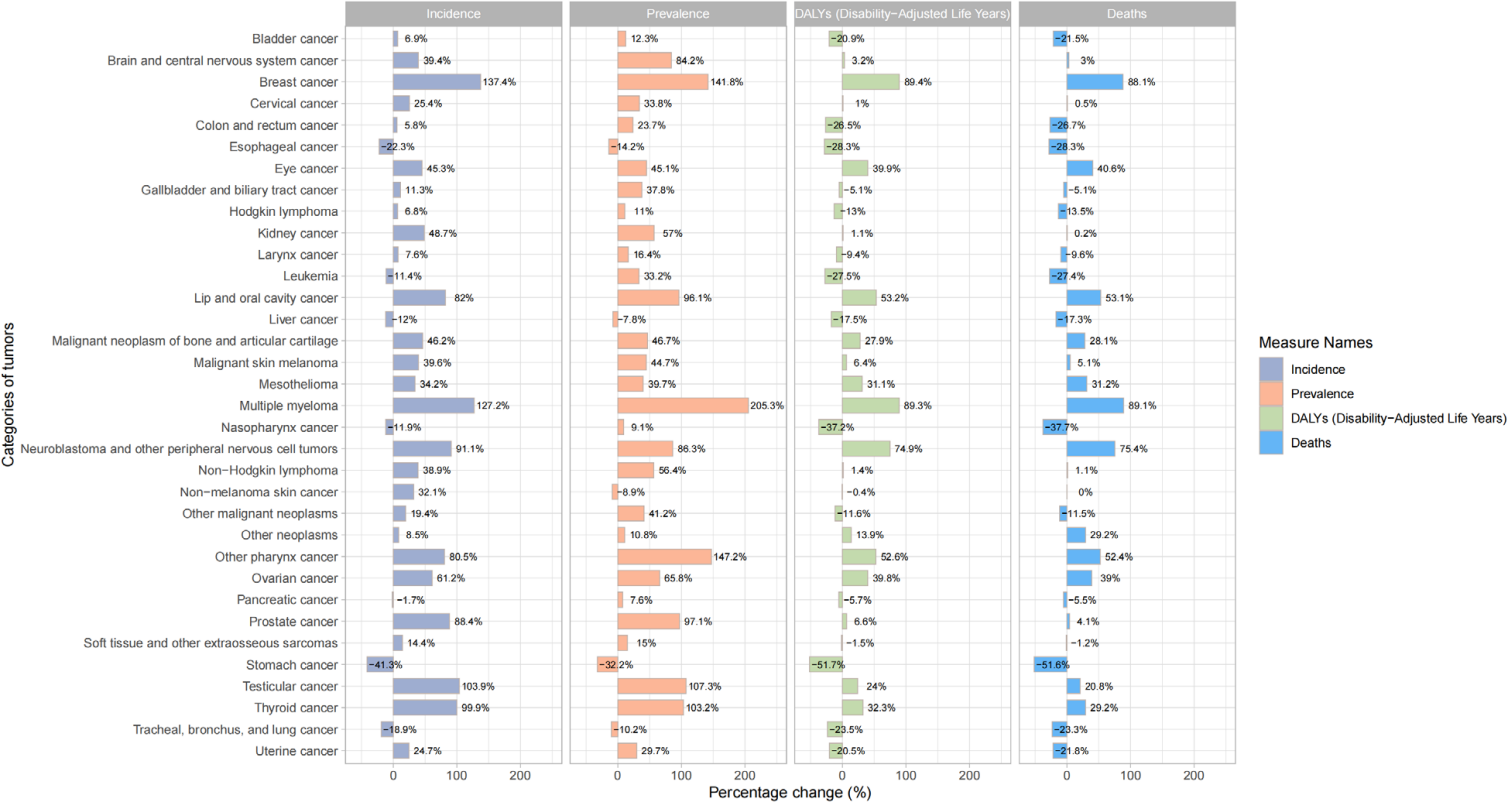
Percentage change in incidence, prevalence, DALYs and deaths of different cancers among adolescents and young adults aged 10–24 years from 1990 to 2021.

In terms of ASIR, the top three cancers in global cancer burden among adolescents and young adults aged 10–24 years in 1990 were acute lymphoid leukemia [1.52, 95% UI:1.21 to 1.8)], brain cancer [1.2, 95%UI: 0.99 to 1.38)] and other lymphoma [0.94, 95% UI: 0.86 to 1.03)]. The top three cancers in 2021 were brain cancer [1.37, 95% UI: 1.16 to 1.63)], acute lymphoid leukemia[1.12, 95% UI: 0.82 to 1.28)] and other lymphoma [1.03, 95% UI: 0.93 to 1.17)] (**Figure 3**). Cancer with the fastest increased incidence from 1990 to 2021 was breast cancer with an increase of 137.4%, followed by multiple myeloma with an increase of 127.2%, and testicular cancer with an increase of 103.9% (**Figure 4**). In terms of ASPR, the top three cancers in 1990 were other lymphoma [6.08, 95% UI:5.59 to 6.62)], malignant neoplasm of bone & articular cartilage]5.9, 95% UI:5.1 to 6.93)] and Hodgkin lymphoma [4.62, 95% UI:4.08 to 4.97)]. The top three cancers in 2021 were other lymphoma [7.59, 95% UI:6.88 to 8.51)], malignant neoplasm of bone & articular cartilage [7.09, 95% UI (6 to 8.52)] and brain cancer [6.84, 95% UI (5.84 to 8)] (**Supplementary Figure 8**). From 1990 to 2021, the top three cancers with the fastest growth in prevalence were multiple myeloma, other pharynx cancer, and breast cancer, with increases of 205.3%, 147.2%, and 141.8%, respectively (**Figure 4**). In terms of ASDR, the top three cancers in 1990 were acute lymphoid leukemia [101.16, 95% UI (78.74 to 121.76)], brain cancer [56.4, 95% UI (44.4 to 66.12)] and acute myeloid leukemia [44.63, 95% UI (29.33 to 60.98)]. In 2021, the top three cancers were acute lymphoid leukemia [57.7, 95% UI (41.19 to 66.42)], brain cancer [47.72, 95% UI (39.33 to 57.3)], and malignant neoplasm of bone & articular cartilage [41.65, 95%UI (35.33 to 51.16)] (**Supplementary Figure 9**). The top three fastest-growing cancers in age-standardized DALYs rate from 1990 to 2021 were breast cancer, multiple myeloma neuroblastoma, and other peripheral nervous cell tumors, with increases of 89.4%, 89.3%, and 74.9% respectively (**Figure 4**). In terms of ASDR, the top three cancers in 1990 were acute lymphoid leukemia [1.37, 95% UI (1.03 to 1.65)], brain cancer [0.77, 95% UI (0.6 to 0.9)], and acute myeloid leukemia [0.61, 95% UI (0.4 to 0.83)]. The top three cancers in 2021 were acute lymphoid leukemia [0.78, 95%UI (0.56 to 0.9)], brain cancer [0.65, 95%UI (0.53 to 0.77)], and malignant neoplasm of bone & articular cartilage [0.57, 95%UI (0.48 to 0.69)] (**Supplementary Figure 10**). The top three fastest-growing cancers in death from 1990 to 2021 were multiple myeloma, breast cancer and neuroblastoma and other peripheral nervous cell tumors, with increases of 89.1%, 88.1%, and 75.4%, respectively. Cancer with the most significant decrease from 1990 to 2021 was stomach cancer, with incidence, prevalence, DALYs, and death decreasing by 41.3%, 32.2%, 51.7%, and 51.6%, respectively (**Figure 4**). **Supplementary Table 5** presented a comprehensive analysis of the incidence, prevalence, DALYs and death numbers for 34 types of tumors from 1990 to 2021.

### Age subgroup analysis of cancer burden

**Figure 5:**
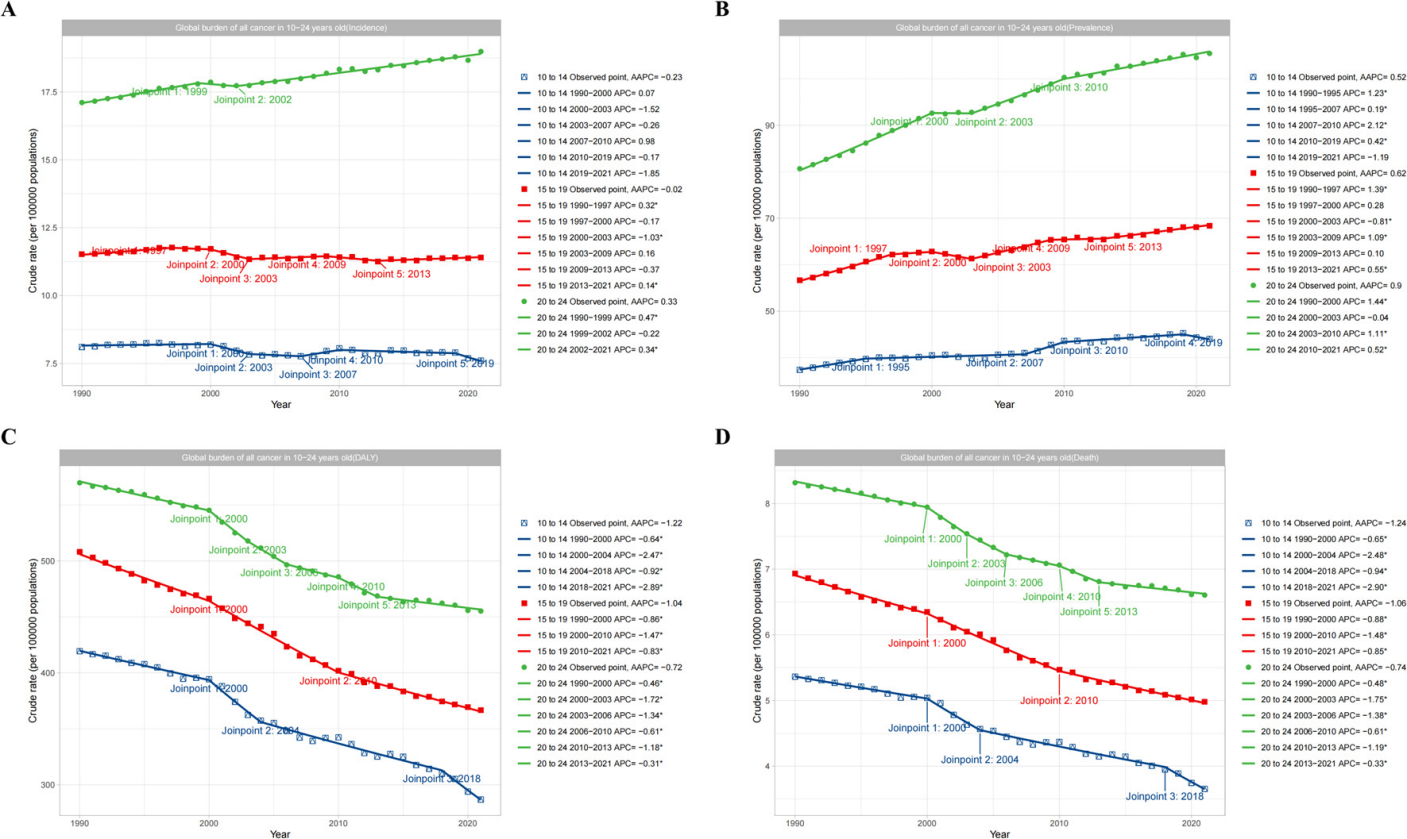
Global trend of total cancers burden according to age subgroups. **(A)** showed the incidence trend of total cancer in different age subgroups. **(B)** showed the prevalence trend of total cancer in different age subgroups. **(C)** showed the DALYs trend of total cancer in different age subgroups. **(D)** showed the deaths trend of total cancer in different age subgroups.

The subgroups of 10-14, 15-19, and 20–24 years were analyzed by *Joinpoint* regression analysis. The results showed that the total cancer burden of young adults aged 20–24 years was significantly higher than adolescents aged 15–19 years and 10–14 years. The total cancer incidence rate of adolescents and young adults aged 20–24 years from 1990 to 2021 showed a yearly increasing trend with an AAPC value of 0.33, while the total cancer incidence of adolescents aged 10–14 years and 15–19 years showed a yearly decreasing trend with AAPC values of -0.02 and -0.23, respectively (**Figure 5A**)(**Figure 5A** showed the incidence trend of total cancer in different age subgroups). In terms of prevalence rate, all subgroups showed an increasing trend from 1990 to 2021, with AAPC values of 0.52, 0.62, and 0.9, respectively (**Figure 5B**) (**Figure 5B** showed the prevalence trend of total cancer in different age subgroups). In terms of the DALY rate, all subgroups showed a decreasing trend from 1990 to 2021, with AAPC values of -1.22, -1.04, and -0.72, respectively (**Figure 5C**) (**Figure 5C** showed the DALYs trend of total cancer in different age subgroups). In terms of mortality rate, all subgroups displayed a decreasing trend from 1990 to 2021, with AAPC values of -1.24, -1.06, and -0.74, respectively (**Figure 5D**) (**Figure 5D** showed the deaths trend of total cancer in different age subgroups).

### Gender subgroup analysis of cancer burden

**Figure 6:**
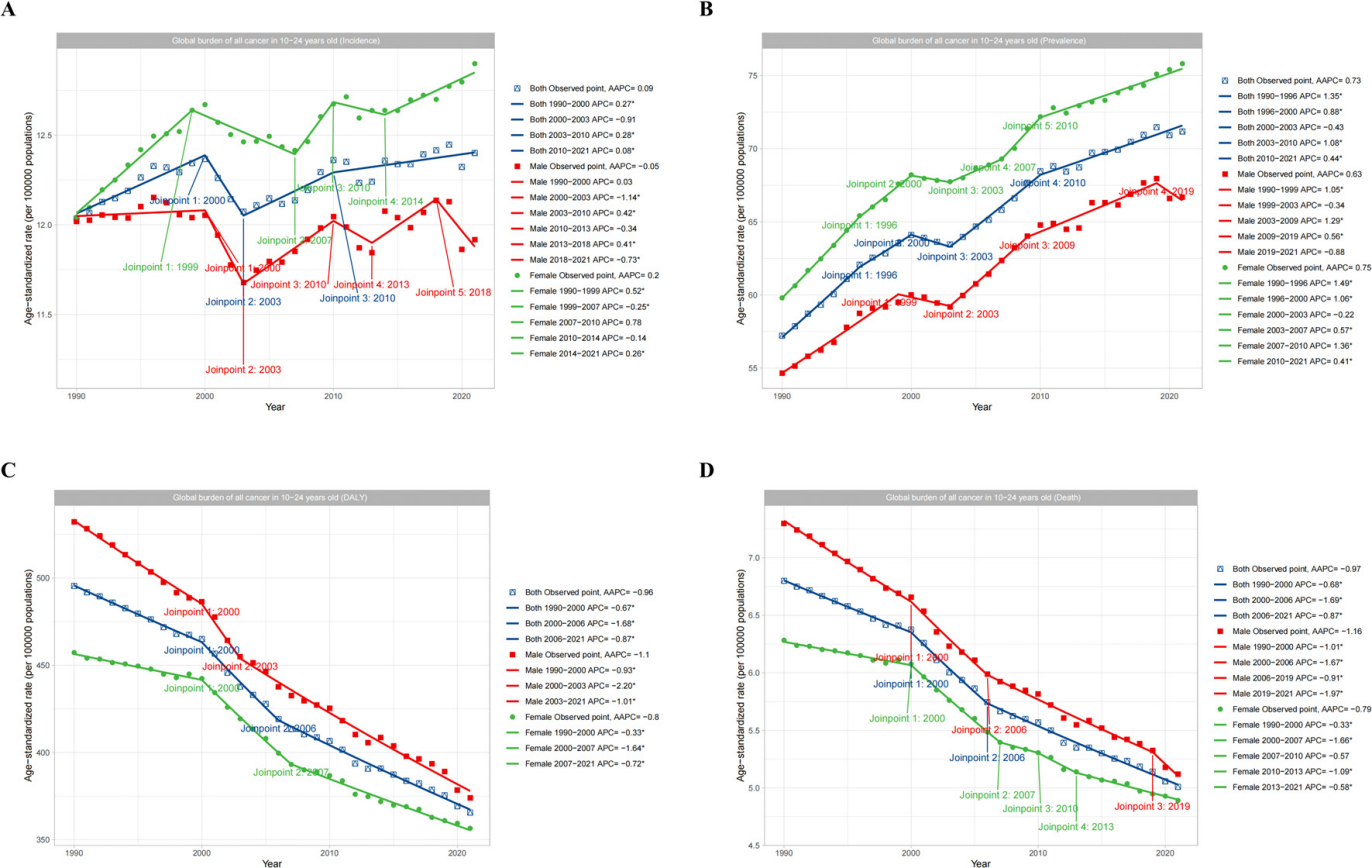
Global trends in total cancers burden by gender subgroups. **(A)** showed the incidence trend of total cancer based on gender. **(B)** showed the prevalence trend of total cancer based on gender. **(C)** showed the DALYs trend of total cancer based on gender. **(D)** showed the deaths trend of total cancer based on gender.

The subgroup analysis of adolescents and young adults aged 10–24 years by gender (Male, Female, and Both) showed that in the term of ASIR, females aged 10–24 years showed an increasing trend from 1990 to 2021, with an AAPC of 0.2, while males showed a decreasing trend, with an AAPC of -0.05. However, the overall trend was increasing, with an AAPC of 0.09 (**Figure 6A**). Similarly, in the term of ASPR, the prevalence of females was significantly higher than males, and both genders showed an increasing trend year by year, with AAPC values of 0.75 and 0.63 for females and males, respectively (**Figure 6B**). However, in terms of ASDR, there was a significant decrease from 1990 to 2021 for both males and females, with AAPC values of -1.1 and -0.8, respectively (**Figure 6C**). In terms of ASMR, there was a significant decrease from 1990 to 2021, with AAPC values of -1.16 and -0.79 for males and females, respectively (**Figure 6D**).

### Health inequity analysis

**Figure 7:**
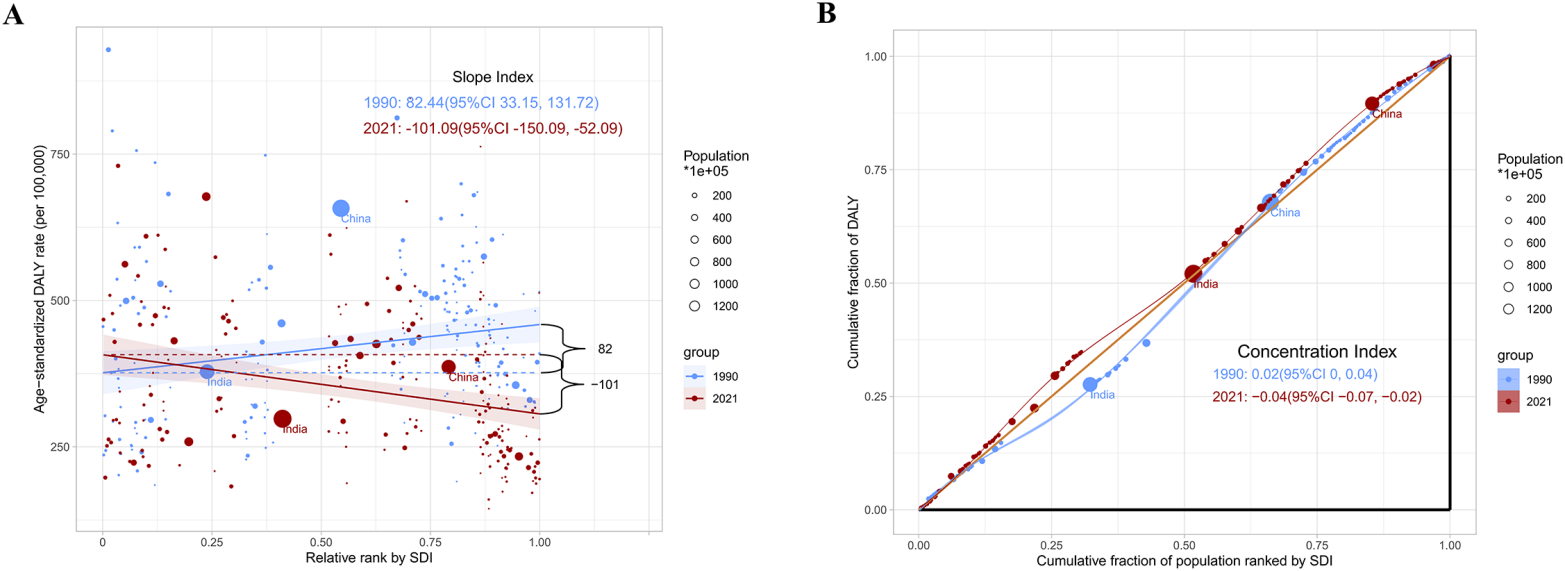
Health inequity analysis. **(A)** showed the absolute health inequities of total cancer burden; **(B)** showed the relative health inequities of total cancer burden.

**Figure 8:**
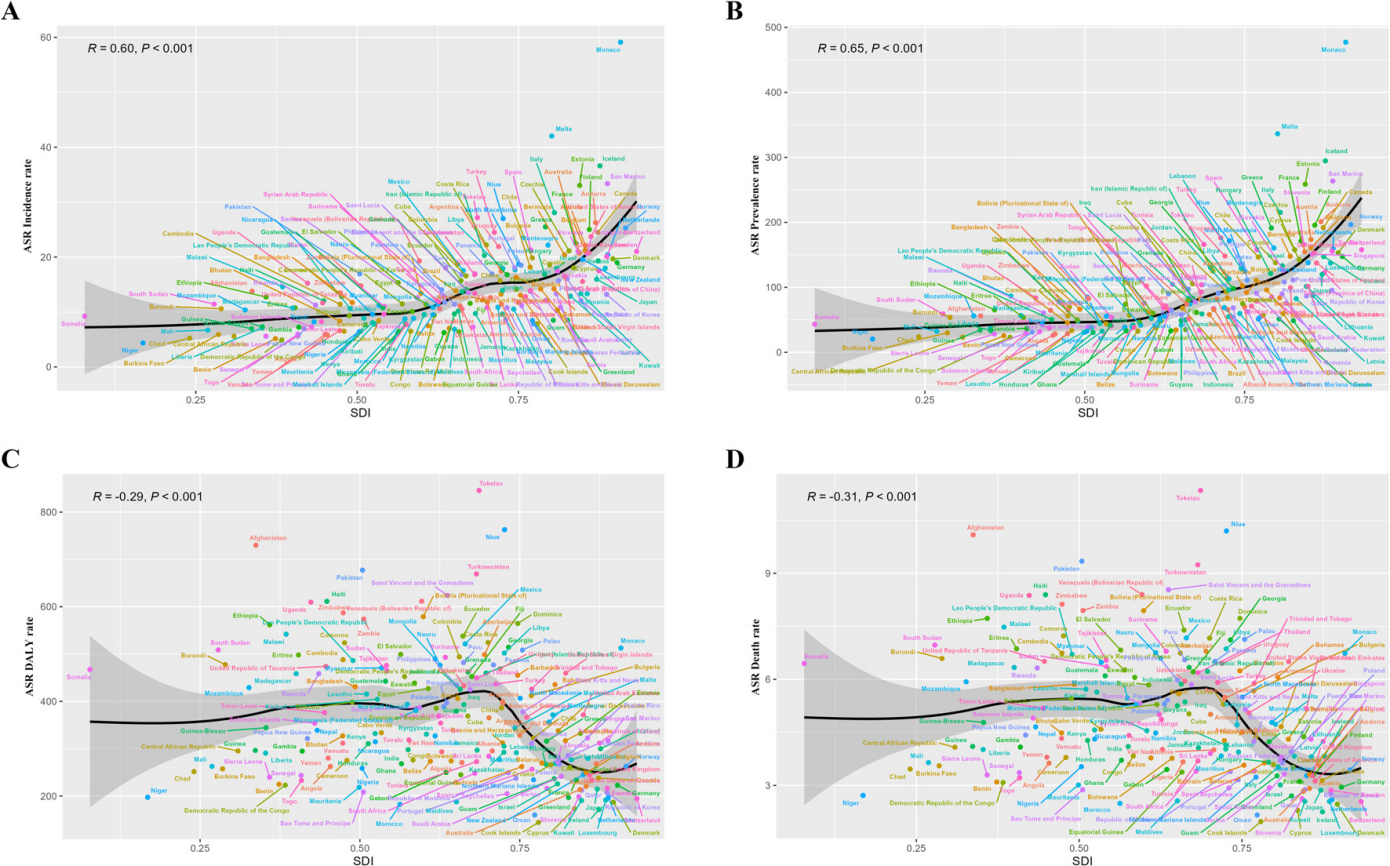
Correlation analysis of ASIR, ASPR, ASDR and age-standardized DALYs rate of total cancer with SDI. **(A)** showed a significant positive correlation between ASIR and SDI (R = 0.60, P<0.001); **(B)** showed a significant positive correlation between ASPR and SDI (R = 0.65, P<0.001); **(C)** showed a significant negative correlation between age-standardized DALYs rate and SDI (R=-0.29, P<0.001); **(D)** showed a significant negative association between ASDR and SDI (R=-0.31, P<0.001).

We performed a health inequity analysis of the total cancer burden in adolescents and young adults aged 10–24 years. The results showed that in terms of age-standardized DALYs rate, SII in 1990 was 82.44(95%CI 33.15 to 131.72), and CII was 0.02(95%CI 0 to 0.04). In 2021, the SII was -101.99 (95% CI-150.09 to 52.09) and the CII was -0.04(95% CI-0.07 to -0.02) (**Figure 7**) (**Figure 7** showed the absolute and relative health inequities of total cancer burden). We analyzed the correlation between ASIR, ASPR, ASDR, age-standardized DALYs rate, and SDI levels, and the results displayed that ASIR and ASPR were positively correlated with SDI (R-0.60, R = 0.65). However, ASDR and age-standardized DALYs rate were negatively correlated with SDI (R=-0.31, R=-0.29) (**Figure 8**)(**Figure 8** showed correlation analysis of ASIR, ASPR, ASDR and age-standardized DALYs rate of total cancer with SDI). It can be seen that the new cancer patients were mainly concentrated in affluent areas. However, the deaths were concentrated in the poorer areas. In addition, we analyzed the correlation between the ASIR, ASPR, ASDR, and age-standardized DALYs rate of the three most lethal cancers globally in 2021 and the SDI. The results showed that for leukemia, the ASIR and ASPR were significantly positively correlated with the SDI(R = 0.43, R = 0.72) (**Supplementary Figure 11**). For brain cancer, the ASIR, ASPR, age-standardized DALYs rate, and ASDR were all significantly positively correlated with the SDI(R = 0.75, R = 0.81, R = 0.48, R = 0.47) (**Supplementary Figure 12**). In malignant neoplasm of bone & articular cartilage, the ASIR, ASPR, age-standardized DALYs rate, and ASDR were significantly negatively correlated with the SDI(R= -0.19, R=-0.18, R= -0.62, R= -0.62) (**Supplementary Figure 13**).

### Decomposing analysis

Through the decomposition analysis of the epidemiological trend of cancer burden in adolescents and young adults aged 10–24 years, the results showed that among the death indicator, the change in death due to aging change was -215.87 (-0.2%), the proportion of the population was 20, 185.56 (19.1%), and the proportion of epidemiological change accounted for -30776.25 (-29.13%). In the DALY indicator, the change in DALY due to aging change was -12212.57(-0.16%), the percentage of the population was 1470406.15(19.12%) and the percentage of epidemiological change was -2228758.35(-28.99%). Among the prevalence indicators, the change in prevalence due to aging change was -3503.53 (-0.39%), the proportion of the population was 219928.31 (24.67%), and the proportion of epidemiological change was 241965.87 (27.14%). Among the incidence indicators, the incidence change due to aging change was -624.28 (-0.33%), the proportion of the population was 41857.81 (22.33%), and the proportion of epidemiological change was 6548.51 (3.49%). Other detailed results can be seen in **Supplementary Figure 14**; **Supplementary Table 6**. In addition, we also analyzed gender subgroup analysis of DALYs in 27 different regions. Detailed results were available in **Supplementary Figure 15**.

### Projections of adolescents and young adults cancer burden to 2050

Using a BAPC model, we predicted cancer incidence, prevalence, death, DALYs, and their corresponding ASRs in adolescents and young adults aged 10–24 years over the next three decades. This projection extended over the next three decades and provided a forward-looking perspective on the potential epidemiological landscape of cancer in this demographic. The results showed that for total cancer incidence, it was 235237.88 (232037.13 to 238438.64) in 2021 and 224903.4 (1318.86 to 473206.11) in 2050. For total cancer prevalence, it was 1349959 in 2021 (1342191.59 to 1357726.41) and 1480707.64 (0 to 3235718.98) in 2050. For total cancer DALYs, it was 6918664.25 (6900872.1 to 6936456.4) in 2021 and 4421074.52 (0 to 9507681.2) in 2050. For total cancer deaths, it was 94864.89 (92853.65 to 96876.13) in 2021 and 62889.41 (653.42 to 129953.09) in 2050. For total cancer ASIR, it was 12.4 (12.23 to 12.57) in 2021 and 11.06 (0.06 to 24.48) in 2050. For total cancer ASPR, it was 71.18 (70.77 to 71.59) in 2021 and 76.46 (0 to 167.58) in 2050. For the total cancer age-standardized DALYs rate, it was 365.6 (364.65 to 366.54) in 2021 and 230.76 (0 to 498.2) in 2050. For total cancer ASDR, it was 5.01 (4.9 to 5.12) in 2021 and 3.27(0.03 to 6.78) in 2050. Detailed results can be found in **Supplementary Figure 16**; **Supplementary Table 7**. The model projects a continued global rise in both incidence and mortality, with particularly steep increases anticipated in low- and middle-SDI regions. Worldwide, the incidence of adolescents and young adults cancer is expected to rise by approximately 4.6% compared with 2021, reaching 235249 cases by 2050, while cancer-related deaths are projected to increase by 50.8%, reaching 94856. Marked regional disparities are evident. High-SDI regions are expected to experience only modest increases, reflecting plateauing trends driven by effective screening and treatment programs. In contrast, low- and middle-SDI regions are likely to bear the greatest future burden, with disproportionately higher growth rates in both incidence and mortality. The projected DALY patterns parallel these trends, with the largest increases concentrated in areas with limited healthcare infrastructure and incomplete cancer registry coverage. These projections should, however, be interpreted with caution. The BAPC model assumes stable demographic and healthcare conditions over time. Unanticipated public health interventions—such as expanded HPV vaccination programs, introduction of novel therapeutics, or improvements in cancer registration could substantially alter future trajectories. Thus, while our projections provide valuable directional insight into the rising burden of adolescents and young adults cancer, they should be viewed as indicative rather than definitive forecasts.

## Discussion

Some previous studies revealed the epidemiological characteristics of the global cancer burden, but they only focused on the cancer burden at the regional, national level or specific age groups, and there were still gaps in understanding the global cancer burden in adolescents and young adults aged 10–24 years (17–20). Studying the global cancer burden in adolescents and young adults aged 10–24 years following the COVID-19 pandemic was critical for a comprehensive understanding of global cancer epidemiology in the age group.

To our knowledge, this was the first up-to-date and most comprehensive systematic analysis to assess the cancer burden in adolescents and young adults aged 10–24 years over the past three decades at global, regional, and national levels. We found that in 2021, there were 235249.05 new cancer cases in adolescents and young adults worldwide, 1349913.04 prevalent cases, 6918657.72 DALYs, and 94856.02 deaths. According to our study, the global incidence and prevalence of cancer in adolescents and young adults has increased significantly over the past three decades, although death and DALYs decreased. Our study also found that the prevalence of cancer in adolescents and young adults would continue to increase over the next three decades. In addition, we revealed that the prevalence and incidence of total cancer were significantly higher at ages 20–24 years than those ages 15–19 years and 10–14 years, indicating that the likelihood of developing cancer increases significantly with age. Our analysis further revealed a pronounced gender disparity in cancer prevalence and incidence rates, with females exhibiting significantly higher figures compared with males, underscoring the substantial differences in cancer burden between the genders. Adolescents and young adults face significant health challenges, and effective strategies 2 reduce the burden of cancer in adolescents and young adults aged 10–24 years.

Although adolescent and young adult cancer patients received better treatment and care, they were still likely to experience tumor recurrence and other secondary diseases (21). Compared with patients without cancer, they would experience more potential threats such as treatment pressure, psychological pressure, and physical function damage (22–25). In addition, the life expectancy of those with cancers was generally significantly lower than normal people (26, 27). Our findings were the first study to identify the global cancer burden in adolescents and young adults aged 10–24 years, providing a reliable reference for establishing effective cancer control systems in different places and reducing the cancer burden.

The increasing global cancer burden in adolescents and young adults aged 10–24 years was alarming. Our study found that the cancer burden in adolescents and young adults varied widely across regions and countries. The ASIR and ASIR of cancers in adolescents and young adults with moderate or high SDI were more pronounced than in the regions and countries with low SDI. However, the ASDR and age-standardized DALYs rate decreased significantly with increasing SDI. In economically well-off countries and regions, advanced medical screening tools made it easier to detect diseases, resulting in a significant increase in prevalence (28, 29). At the same time, advanced treatments may also lead to better treatment and prognosis in adolescents and young adults (30). Our study also showed that adolescents and young adults had lower cancer incidence in regions and countries with low SDI, which should be attributed to the fact that cancer incidence was often underestimated due to potential misdiagnosis and underdiagnosis and weak cancer surveillance systems in settings with limited medical care (31). The ASDR and age-standardized DALYs rate were significantly higher in adolescents and young adults in low SDI areas. Inadequate medical care in these areas contributed to misdiagnosis, delayed diagnosis, underdiagnosis, and poor treatment outcomes (32, 33). Given that many of the world’s young populations live in areas with average or poor economic status, regions at different levels of development need to collaborate to reduce healthcare disparities. In addition, the incidence and prevalence rate of total cancer among adolescents and young adults differed significantly among different regions, indicating that apart from SDI, other factors, such as genetic and environmental factors, may also play an important role in the cancer burden among adolescents and young adults aged 10–24 years (34, 35).

Leukemia, brain cancer, and malignant neoplasm of bone & articular cartilage were the most important causes of death and DALYs in adolescents and young adults aged 10–24 years in 2021. Acute lymphoblastic leukemia was the most common type of leukemia and the leading cause of death and DALYs in adolescents and young adults. A variety of factors, including genetic risk and environmental exposures, could help explain why the incidence of acute lymphoblastic leukemia in adolescents and young adults continues to rise (36). Survival rates for patients with leukemia improved dramatically with continued advances in treatment (37). However, therapeutic remission rates of leukemia in adolescents and young adults were significantly worse than in children (38). In addition, high SDI regions had a higher incidence and prevalence of leukemia in adolescents and young adults aged 10–24 years. However, there was no significant correlation between ASDR, age-standardized DALYs rate, and SDI.

For brain cancer and malignant neoplasm of bone & articular cartilage, we found that the ASIR and ASPR of adolescents and young adults were higher in the high SDI region. The main reasons for the increased incidence of brain cancer and malignant neoplasm of bone & articular cartilage may be genetic factors, environmental influences, and the improvement of diagnostic levels (39, 40). However, countries with higher SDI had the highest ASDR and age-standardized DALYs rate for adolescents and young adults with brain cancer. A previous GBD study found an increased incidence of brain cancer in the highest ASIR and ASDR in high-income regions (Europe and America), while the lowest was observed in the African Region at all ages (41), which was consistent with our study. Studies revealed that Caucasians had a higher risk of brain cancer (42). The results suggested that these facts cannot be analyzed by SDI alone and may be related to genetic differences, environmental factors, and race. The burden of bone malignancy is on the rise globally (43). Our study also found that bone malignancies became one of the most important causes of death and DALYs for adolescents and young adults in 2021. However, we found that bone malignancies in low SDI regions had higher ASIR, ASPR, ASDR, and age-standardized DALYs rate.

Our findings suggested that stomach cancer was the cancer with the most significant decline in ASIR in adolescents and young adults aged 10–24 years over the past three decades. Studies showed that stomach cancer was associated with dietary habits and helicobacter pylori infection (44). We hypothesized that the large decline in stomach cancer was most likely due to the widespread use of antibiotics and the popularity of good dietary habits. We also found that the tumor with the fastest-growing ASIR and ASDR was breast cancer. Breast cancer was most common in women of reproductive age, and 674199.41 women died of breast cancer in 2021 (45). Our findings showed that there was a significant trend towards the younger age of onset of breast cancer. In addition, we found that the cancer with the fastest-growing ASPR and ASMR was multiple myeloma. Multiple myeloma is a rare malignant tumor that is most common in middle-aged and older adults (46). Data suggested that multiple myeloma presented a significant upward trend globally (47). Our results revealed that there was also a significant trend towards younger age of multiple myeloma, which may be due to a combination of lifestyle habits, environment, and genetics. Multiple myeloma may become the most significant threat to adolescents and young adults in the future. Therefore, it was necessary to take timely measures to control this trend.

The quality and availability of the data included in the GBD varies by country, geographic location, and disease etiology. This difference would inevitably affect the accuracy of the research results. Some economically disadvantaged areas may underreport adolescent and young adult cancer patients due to poor diagnostic techniques. Underreporting remains a critical concern, especially in low- and middle-SDI regions where cancer registries are incomplete or absent and where death certification may lack accuracy. This underreporting likely leads to underestimation of both incidence and mortality, suggesting that the true disparities in global adolescents and young adults cancer burden may be even more pronounced than our results indicate. Moreover, underreporting may distort temporal patterns, producing artificial dips or delays in observed trends.Beyond statistical patterns, structural and contextual factors play a critical role in shaping disparities in adolescents and young adults cancer burden. Limited access to timely diagnosis and treatment in resource-constrained settings contributes to worse survival outcomes. Organized cancer screening programs, which are prevalent in high-SDI regions, remain underdeveloped in many low- and middle-SDI regions, resulting in late-stage detection. Health education and awareness campaigns are also unevenly distributed, affecting symptom recognition and care-seeking behavior.Furthermore, environmental and behavioral risk factors-including tobacco use, alcohol consumption, dietary practices, and exposure to pollution-vary across economic contexts and further contribute to observed disparities.

Our research had some obvious limitations.Firstly, The projections of BAPC model are based on the assumption that past trends in age, period, and cohort effects will continue into the future. As such, the model cannot account for unforeseen factors such as novel preventive measures. Secondly, tumors were influenced by racial or genetic factors. The GBD study was unable to specifically analyze the impact of these factors on the cancer burden in adolescents and young adults. In addition, GBD 2021 incorporated data from the aftermath of the COVID-19 pandemic. The strain on healthcare systems and the scarcity of medications during the pandemic could lead to an overestimation of COVID-19-related fatalities, potentially skewing the accuracy of cancer incidence and mortality data for adolescents and young adults (48). Finally, only the GBD database was analyzed in this study. Combining analysis with other databases would better complement the shortcomings of a single database.

We conducted a comprehensive and systematic assessment of the global cancer burden in adolescents and young adults using the latest available GBD data. Our current study found that the global adolescent and young adult cancer burden has been rising over the past three decades. Countries and regions with different SDI levels show markedly different burdens. In low SDI countries, the cancer burden among adolescents and young adults was significantly higher. For low-SDI regions, we suggest strengthening cancer registry systems, implementing cost-effective screening approaches (e.g., VIA for cervical cancer), and developing culturally appropriate awareness campaigns. For middle-SDI regions, we recommend improving referral pathways, expanding access to essential medicines, and scaling up early-detection programs. For high-SDI regions, we emphasize continued innovation in personalized oncology and survivorship care, alongside cross-border knowledge transfer. We also highlight the potential of global partnerships (e.g., WHO’s Global Initiative for Childhood Cancer) to be adapted for adolescents and young adults. These findings underscore the need for proactive measures to address the challenges of the global adolescent and young adult cancer burden. We hope this study will inform government policymakers to plan and implement prevention and control strategies to significantly improve the cancer burden in adolescents and young adults globally.

## Data Availability

The original contributions presented in the study are included in the article/**Supplementary Material**. Further inquiries can be directed to the corresponding author.
