# Supplementary material for: Global burden of cancer in adolescents and young adults aged 10–24 years: a trend analysis
Source: Front Oncol. 2025 Nov 3;15:1663523. doi: 10.3389/fonc.2025.1663523 (PMC12620213; doi:10.3389/fonc.2025.1663523)
Supplement: Supplementary file 1 [file DataSheet1.pdf]

Supplementary materials

Title: Global burden of cancer in adolescents and young adults aged 10-24 years: a trend analysis

Supplementary figure 0. The ASIR, ASPR, ASDR and age-standardized DALY rate of cancers in adolescents and young adults aged 10-24 years in 1990.

Supplementary figure 1. Comparison of ASPR of total cancer in adolescents and young adults aged 10-24 years from1990 to 2021 in different countries.

Supplementary figure 2. Comparison of age-standardized DALYs rate of total cancer cancer in adolescents and young adults aged 10-24 years from 1990 to 2021 in different countries.

Supplementary figure 3. Comparison of ASDR of total cancer cancer in adolescents and young adults aged 10-24 years from 1990 to 2021 in different countries.

Supplementary figure 4. Comparison of cancer incidence number and rate of 26 regions from 1990 to 2021.

Supplementary figure 5. Comparison of cancer prevalence number and rate of 26 regions from 1990 to 2021.

Supplementary figure 6. Comparison of cancer DALYs number and rate of 26 regions from 1990 to 2021.

Supplementary figure 7. Comparison of cancer deaths number and rate of 26 regions from 1990 to 2021.

Supplementary figure 8. Comparison of the ASPR of 34 types of cancers in the 10-24 years age group from1990 to 2021.

Supplementary figure 9. Comparison of the age-standardized DALYs rate of 34 types of cancers in the 10-24 years age group from 1990 to 2021.

Supplementary figure 10. Comparison of the ASDR of 34 types of cancers in the 10-24 years age group from 1990 to 2021.

Supplementary figure 11. Correlation analysis between ASIR, ASPR, ASDR and

age-standardized DALYs rate of leukemia and SDI level.

Supplementary figure 12. Correlation analysis between ASIR, ASPR, ASDR and age-standardized DALYs rate of brain cancer and SDI levels.

Supplementary figure 13. Correlation analysis between ASIR, ASPR, ASDR and age-standardized DALYs rate of malignant neoplasm of bone & articular cartilage and SDI levels.

Supplementary figure 14**.** Decomposition analysis of incidence, prevalence, DALYs and deaths of cancers burdens in adolescents and young adults aged 10-24 years.

Supplementary figure 15. Gender subgroup decomposition analysis of total cancer DALYs in the 26 regions from 1990 to 2021.

Supplementary figure 16. BAPC analysis of total cancers burdens in adolescents and young adults aged 10-24 years.

Supplementary Table 1: AAPC Values of Cancer Disease Burden Among Adolescents Aged 10-24 in the World and 204 Countries/Regions (Incidence).

Supplementary Table 2: AAPC Values of Cancer Disease Burden Among Adolescents Aged 10-24 in the World and 204 Countries/Regions (Prevalence).

Supplementary Table 3: AAPC Values of Cancer Disease Burden Among Adolescents Aged 10-24 in the World and 204 Countries/Regions (DALY).

Supplementary Table 4: AAPC Values of Cancer Disease Burden Among Adolescents Aged 10-24 in the World and 204 Countries/Regions (Death).

Supplementary Table 5: Distribution of Different Tumors Across Various Measures in 1990 and 2021(Number).

Supplementary Table 6: Results of Decompositional Analysis for the 10-24 Age Group.

Supplementary figure 0. The ASIR, ASPR, ASDR and age-standardized DALY rate of cancers in adolescents and young adults aged 10-24 years in 1990

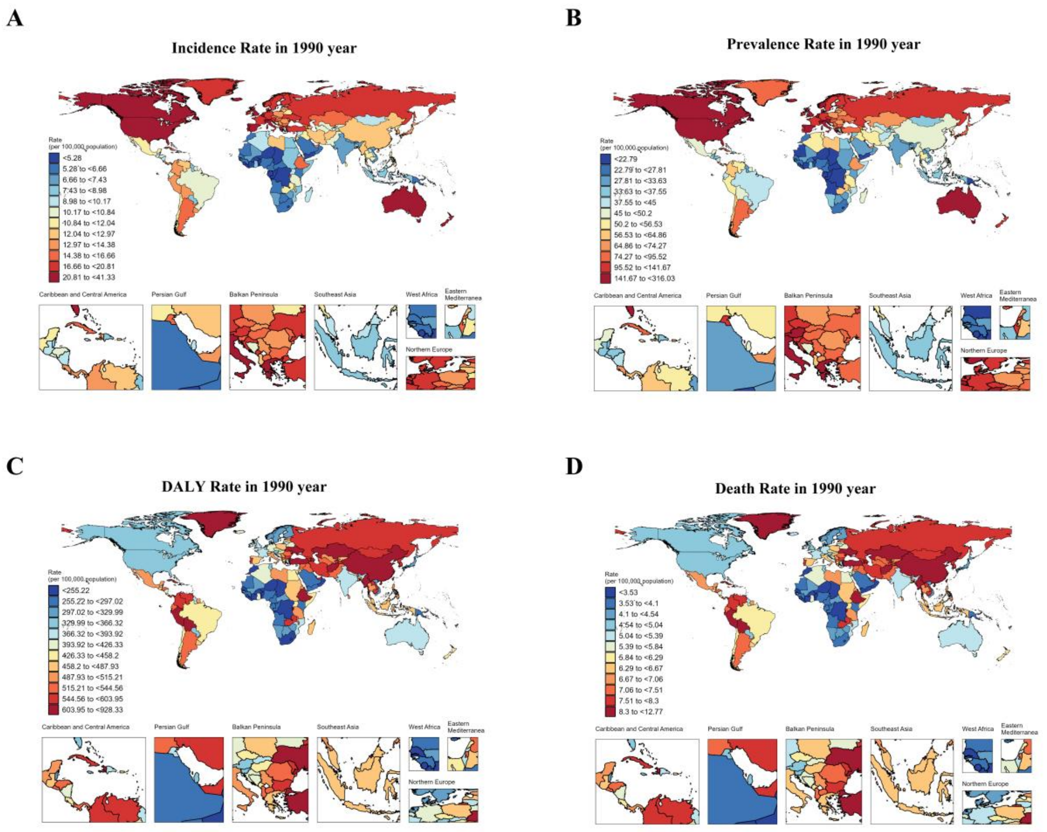

Notes: Supplementary figure 0A showed ASIR of total cancer in adolescents and young adults aged 10-24 years in 1990. Supplementary figure 0B showed ASPR of total cancer in adolescents and young adults aged 10-24 years in 1990. Supplementary figure 0C showed age-standardized DALYs rate of total cancer in adolescents and young adults aged 10-24 years in 1990. Supplementary figure 0D showed ASDR of total cancer in in adolescents and young adults aged 10-24 years in 1990.

Supplementary figure 1. Comparison of ASPR of total cancer in the 10-24 years age group from1990 to 2021 in different countries.

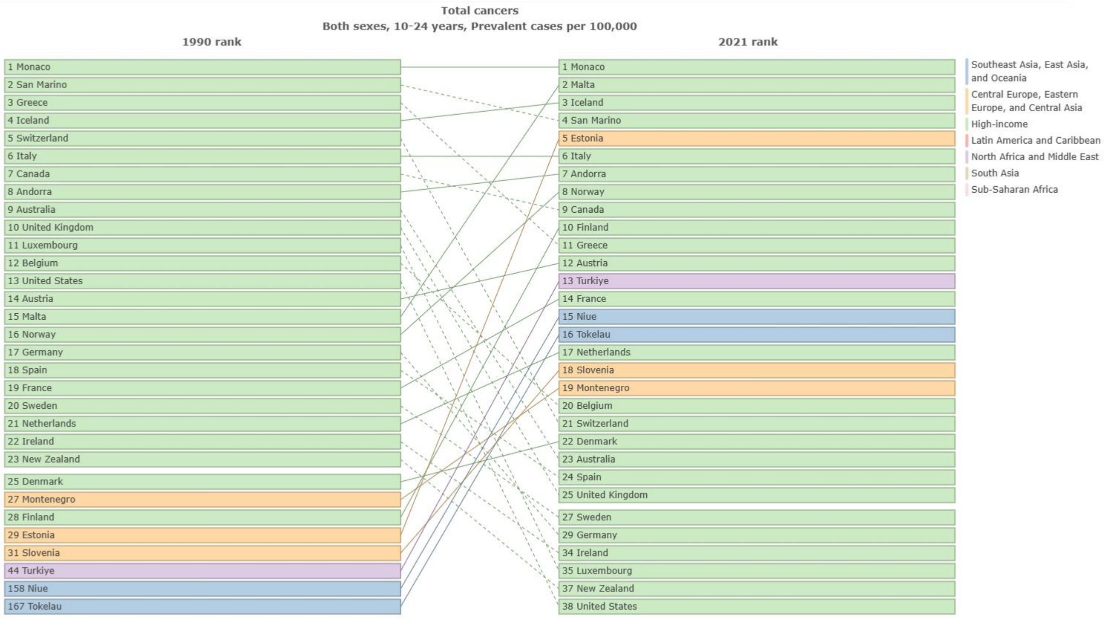

Abbreviation: ASPR, age-standardized prevalence rate.

Supplementary figure 2. Comparison of age-standardized DALYs rate of total cancer in the 10-24 years age group from 1990 to 2021 in different countries.

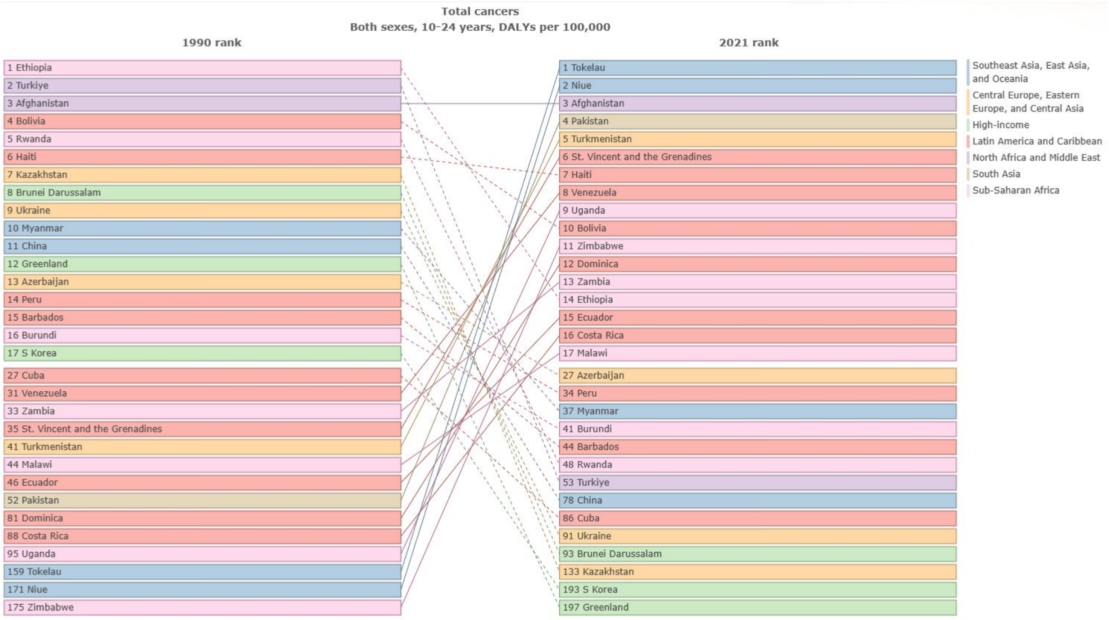

Abbreviation: DALYs, disability-adjusted life years.

Supplementary figure 3. Comparison of ASDR of total cancer in the 10-24 years age group from 1990 to 2021 in different countries.

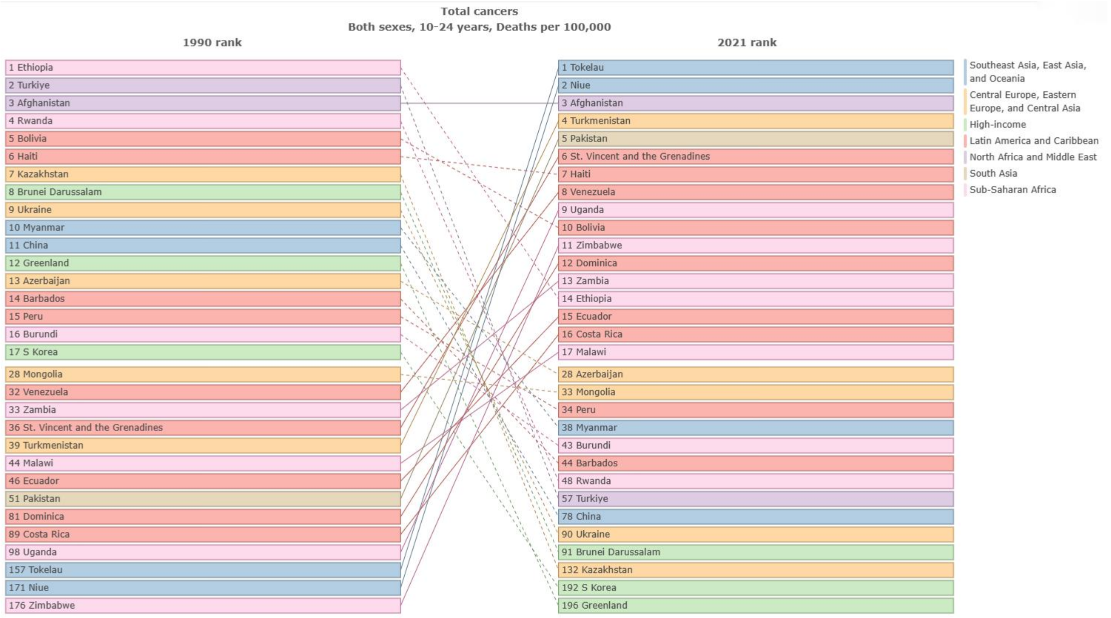

Abbreviations: ASDR, age-standardized death rate.

Supplementary figure 4. Comparison of cancer incidence number and rate of 26 regions from 1990 to 2021.

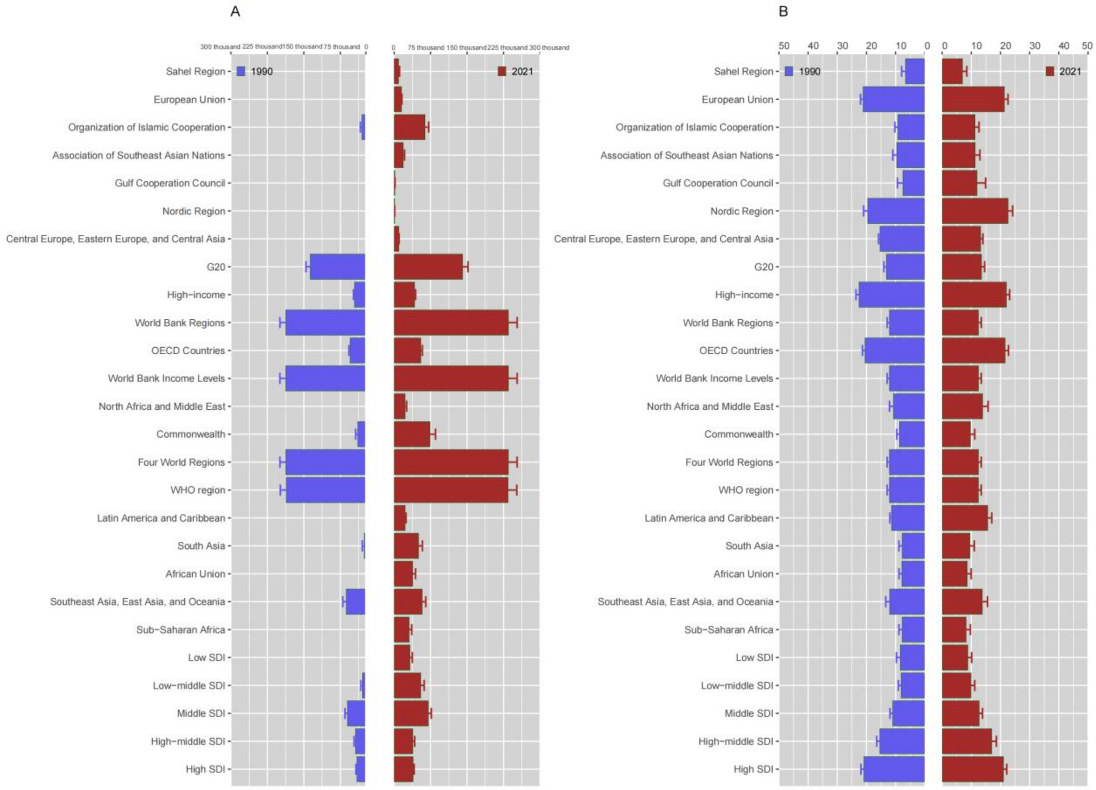

Notes: Supplementary Figure 4A represents a comparison of the total cancer incidence number changes in 26 global regions between 1990 and 2021. Supplementary Figure 4B represents a comparison of the total cancer ASIR changes in 26 global regions between 1990 and 2021.

Abbreviations: ASIR, age-standardized incidence rate.

Supplementary figure 5. Comparison of cancer prevalence number and rate of 26 regions from 1990 to 2021.

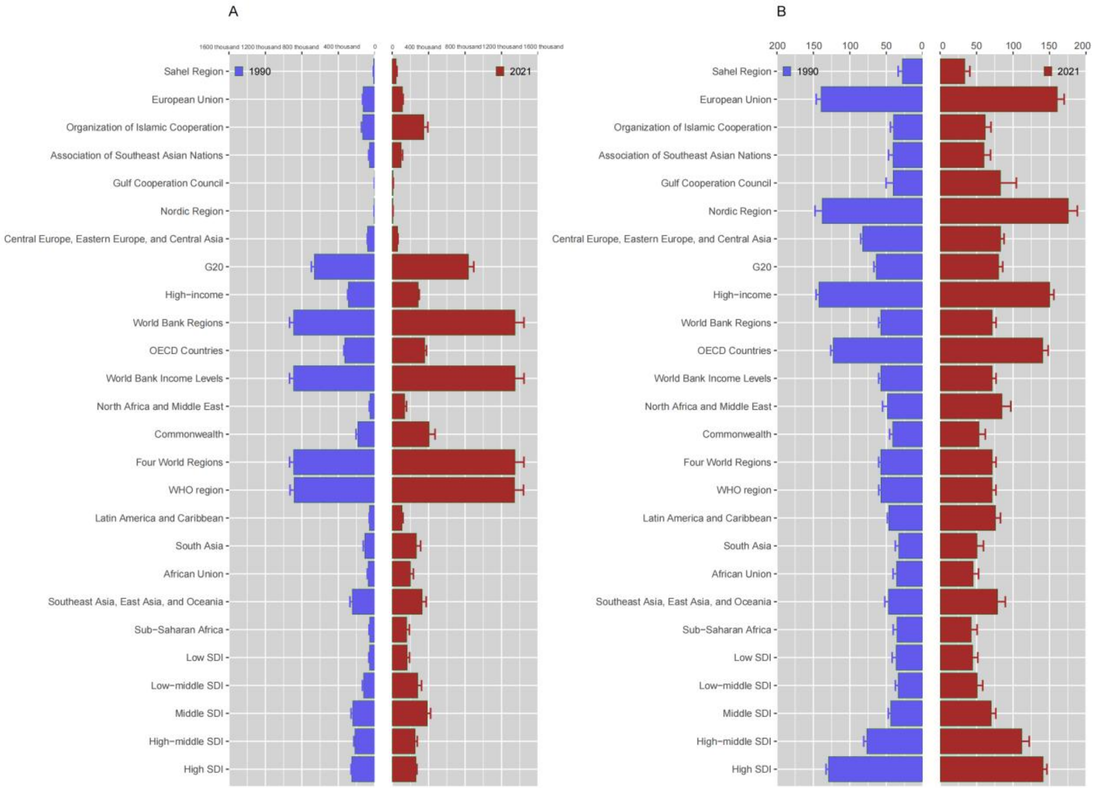

Notes: Supplementary Figure 5A represents a comparison of the total cancer prevalence number changes in 26 global regions between 1990 and 2021. Supplementary Figure 5B represents a comparison of the total cancer ASPR changes in 26 global regions between 1990 and 2021.

Abbreviations: ASPR, age-standardized prevalence rate.

Supplementary figure 6. Comparison of cancer DALYs number and rate of 26 regions from 1990 to 2021.

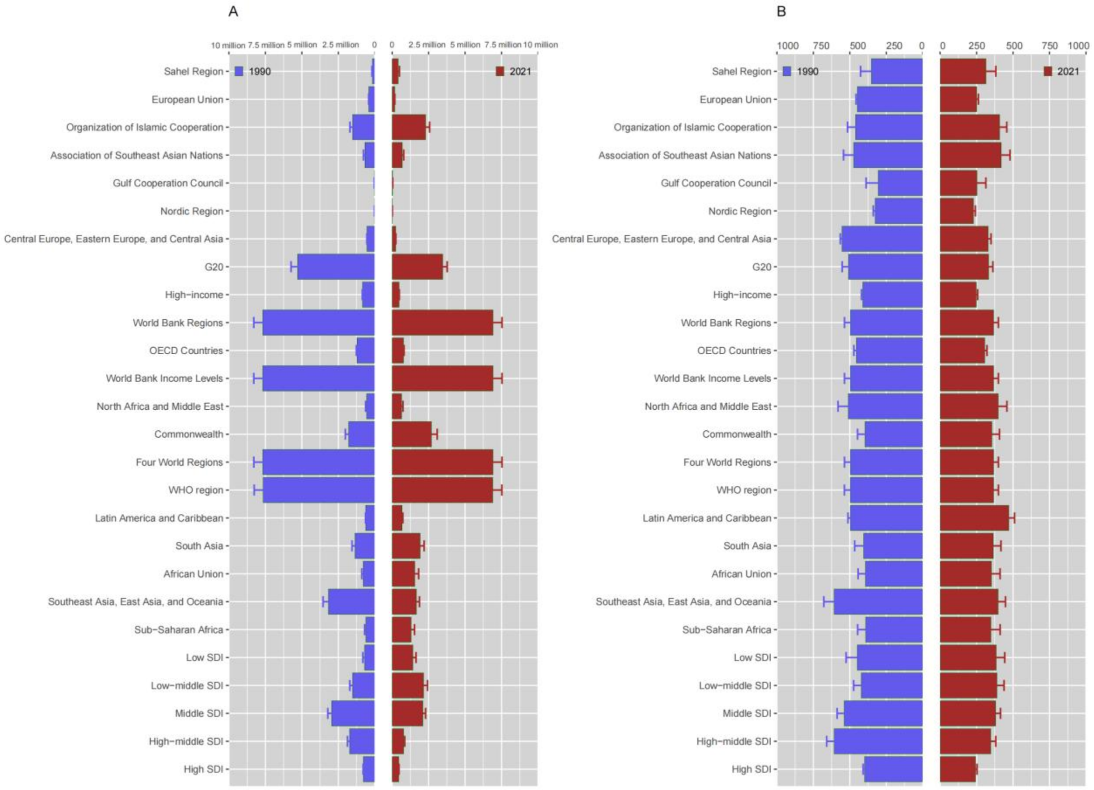

Notes: Supplementary Figure 6A represents a comparison of the total cancer DALY number changes in 26 global regions between 1990 and 2021. Supplementary Figure 6B represents a comparison of the total cancer ASR of DALY changes in 26 global regions between 1990 and 2021.

Abbreviations: ASR, age-standardized rate; DALY, disability-adjusted life-years.

Supplementary figure 7. Comparison of cancer deaths number and rate of 26 regions from 1990 to 2021.

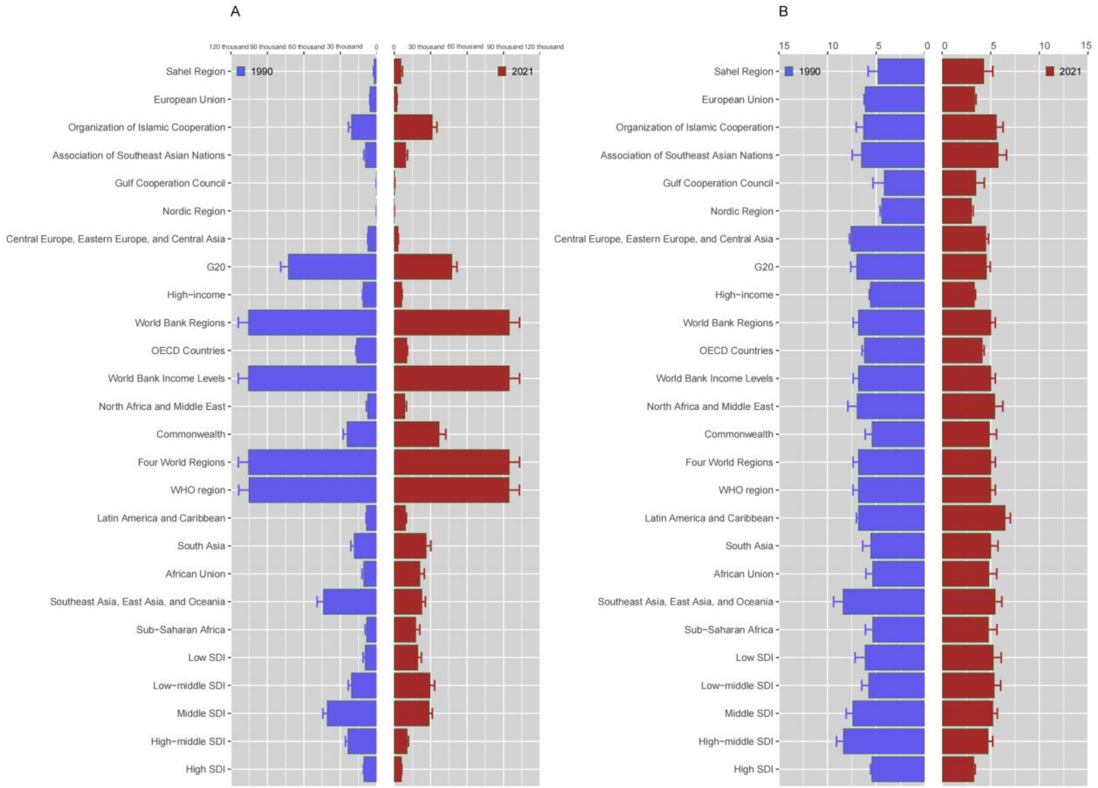

Notes: Supplementary Figure 7A represents a comparison of the total cancer death number changes in 26 global regions between 1990 and 2021. Supplementary Figure 7B represents a comparison of the total cancer ASDR changes in 26 global regions between 1990 and 2021.

Abbreviations: ASDR, age-standardized death rate.

Supplementary figure 8. Comparison of the ASPR of 34 types of cancers in the 10-24 years age group from1990 to 2021.

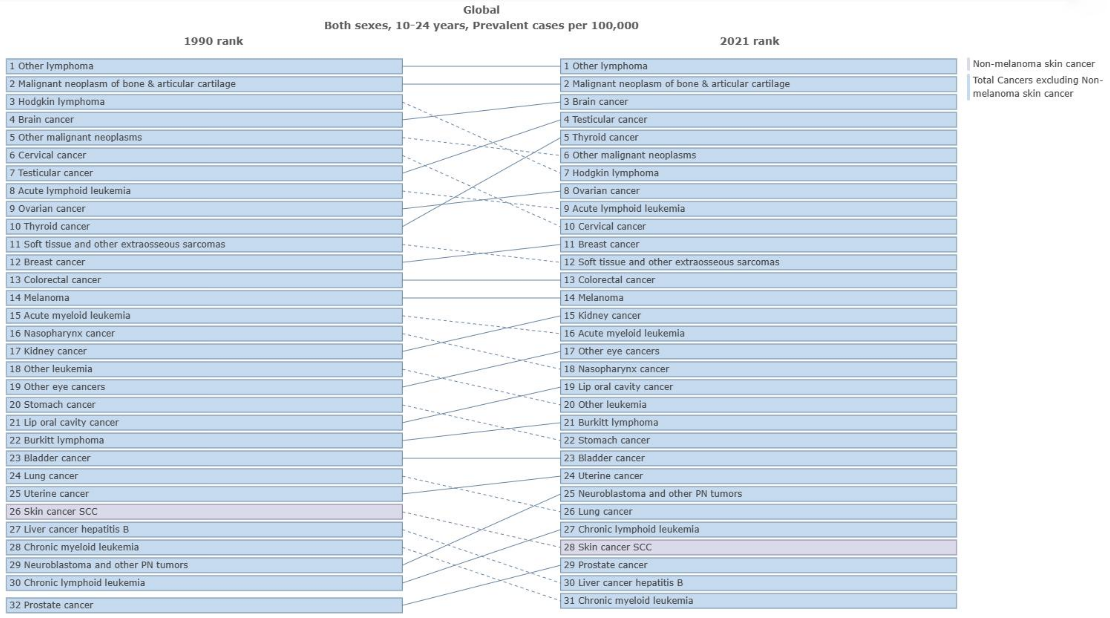

Abbreviations: ASPR, age-standardized prevalence rate.

Supplementary figure 9. Comparison of the age-standardized DALYs rate of 34 types of cancers in the 10-24 years age group from 1990 to 2021.

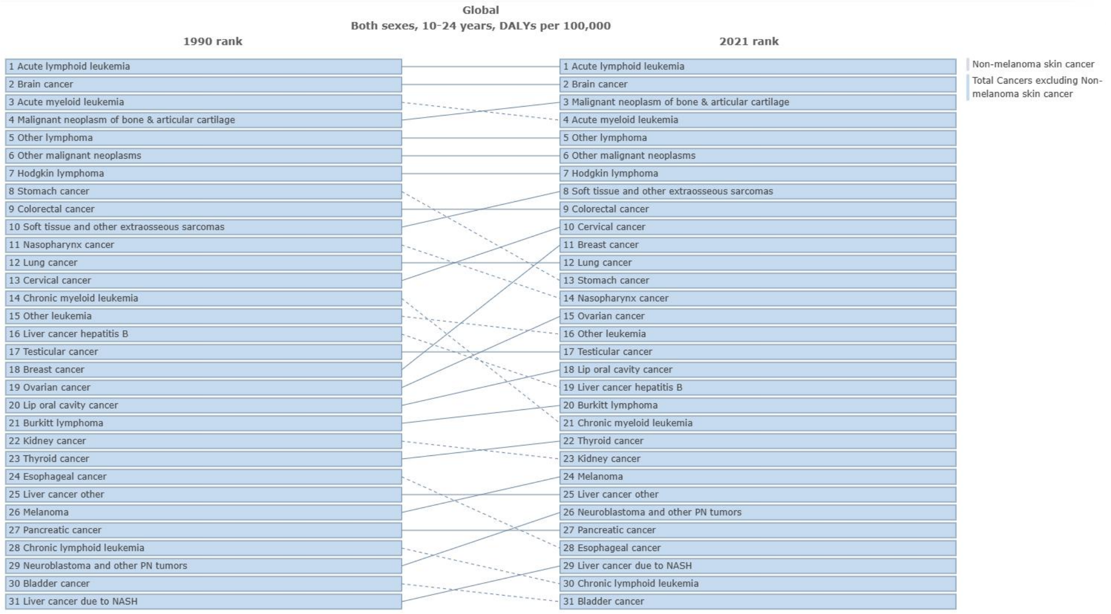

Abbreviation: DALYs, disability-adjusted life years.

Supplementary figure 10. Comparison of the ASDR of 34 types of cancers in the 10-24 years age group from 1990 to 2021.

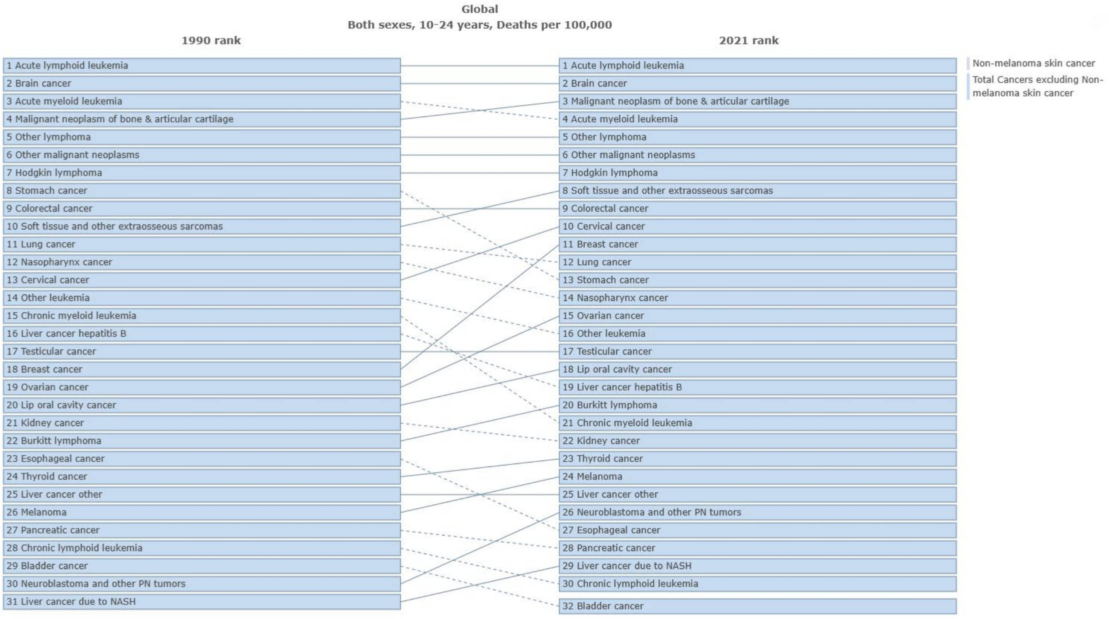

Abbreviations: ASDR, age-standardized death rate.

Supplementary figure 11. Correlation analysis between ASIR, ASPR, ASDR and age-standardized DALYs rate of leukemia and SDI level.

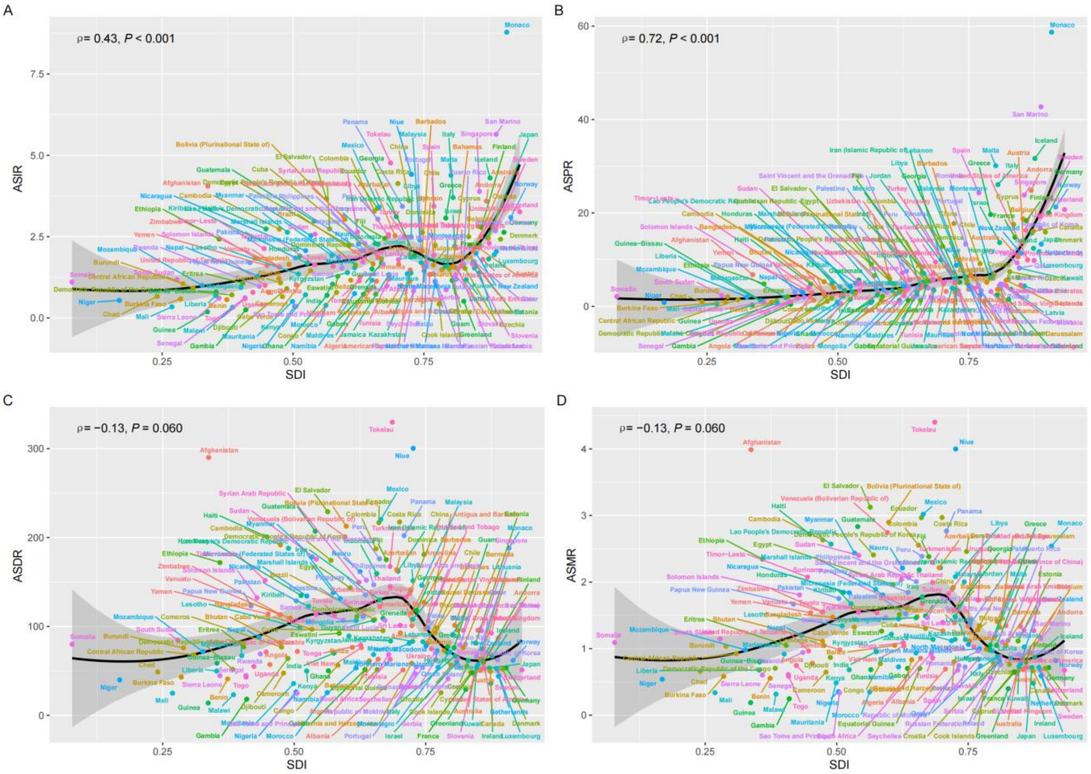

Notes: The ASIR and ASPR of leukemia were significantly positively correlated with the SDI (Supplementary Figures 5A and 5B). However, there was no significant correlation between the ASR of DALY and ASDR with the SDI.

Abbreviations: ASIR, age-standardized incidence rate; ASPR, age-standardized prevalence rate; ASDR, age-standardized death rate; DALYs, disability-adjusted life years; SDI, Socio-Demographic Index.

Supplementary figure 12. Correlation analysis between ASIR, ASPR, ASDR and age-standardized DALYs rate of brain cancer and SDI levels.

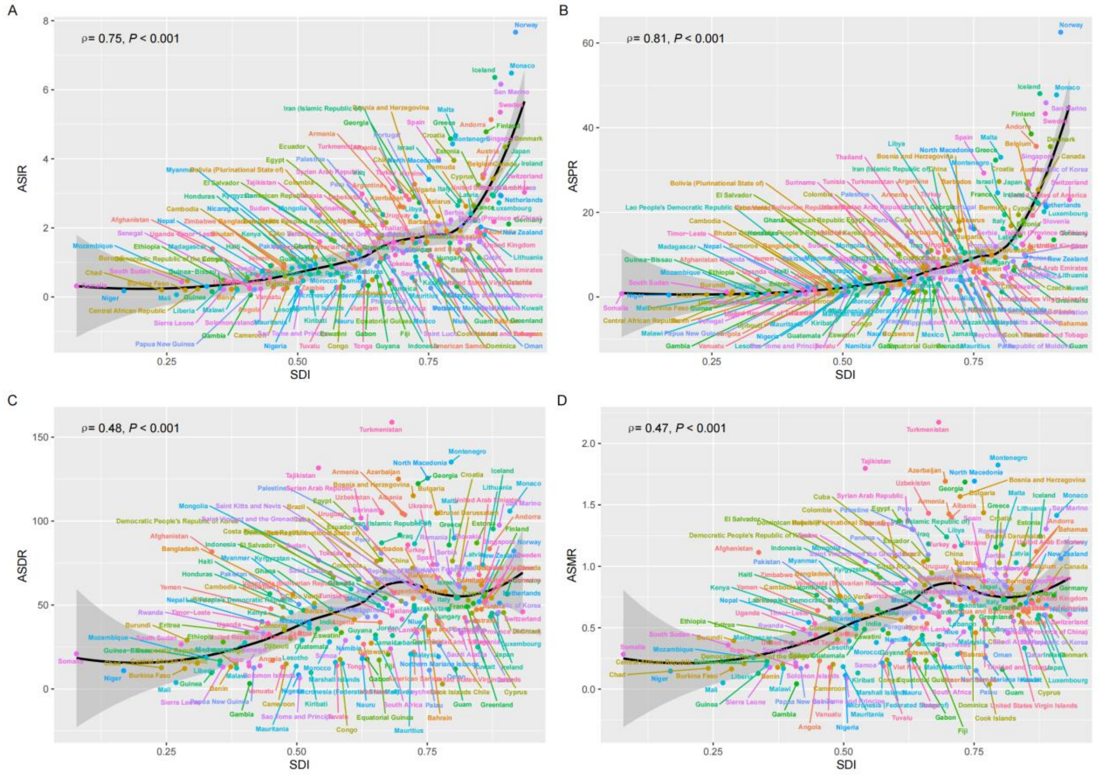

Notes: The ASIR, ASPR, ASR of DALY, and ASDR all showed significant positive correlations with the SDI, with corresponding values of R = 0.75 (P<0.001), R = 0.81 (P<0.001), R = 0.48 (P<0.001), and R = 0.47 (P<0.001), respectively.

Abbreviations: ASIR, age-standardized incidence rate; ASPR, age-standardized prevalence rate; ASDR, age-standardized death rate; DALYs, disability-adjusted life years; SDI, Socio-Demographic Index.

Supplementary figure 13. Correlation analysis between ASIR, ASPR, ASDR and age-standardized DALYs rate of malignant neoplasm of bone & articular cartilage and SDI levels.

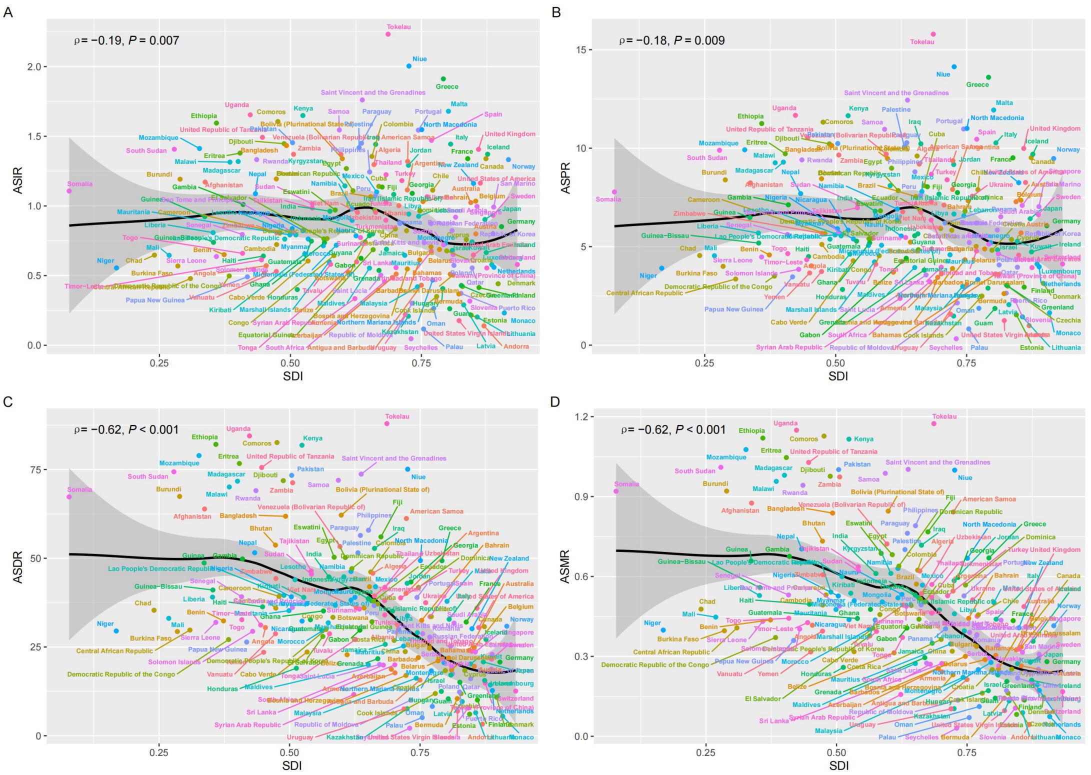

Notes: The ASIR, ASPR, ASR of DALY, and ASDR all showed significant negative correlations with the SDI, with corresponding values of R = -0.19 (P = 0.007), R = -0.18 (P = 0.009), R = -0.62 (P <0.001), and R = -0.62 (P <0.001), respectively.

Abbreviations: ASIR, age-standardized incidence rate; ASPR, age-standardized prevalence rate; ASDR, age-standardized death rate; DALYs, disability-adjusted life years; SDI, Socio-Demographic Index.

Supplementary figure 14**.** Decomposition analysis of incidence, prevalence, DALYs and deaths of cancers burdens in adolescents and young adults aged 10-24 years.

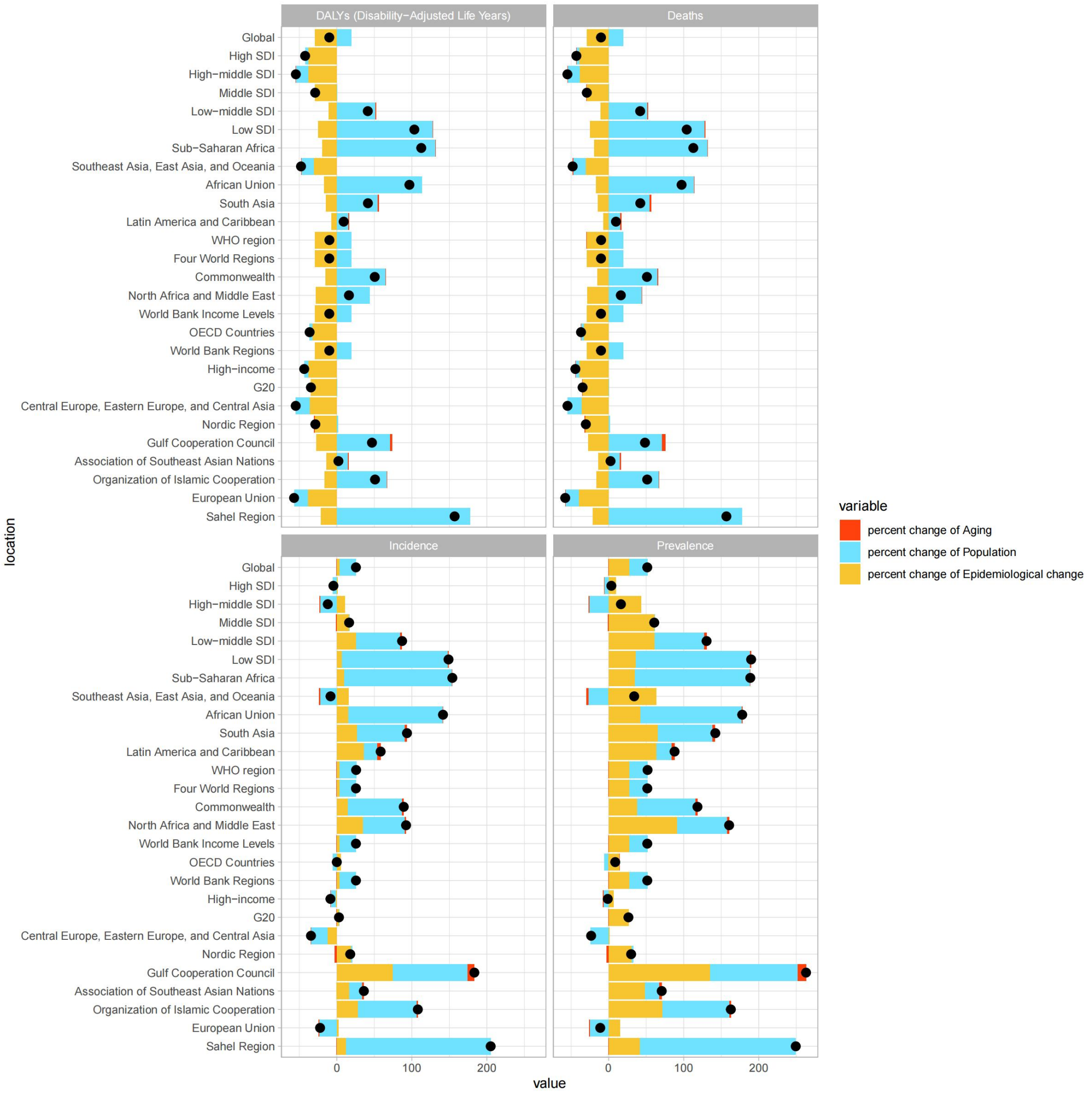

Supplementary figure 15. Gender subgroup decomposition analysis of total cancer DALYs in the 26 regions from 1990 to 2021.

A

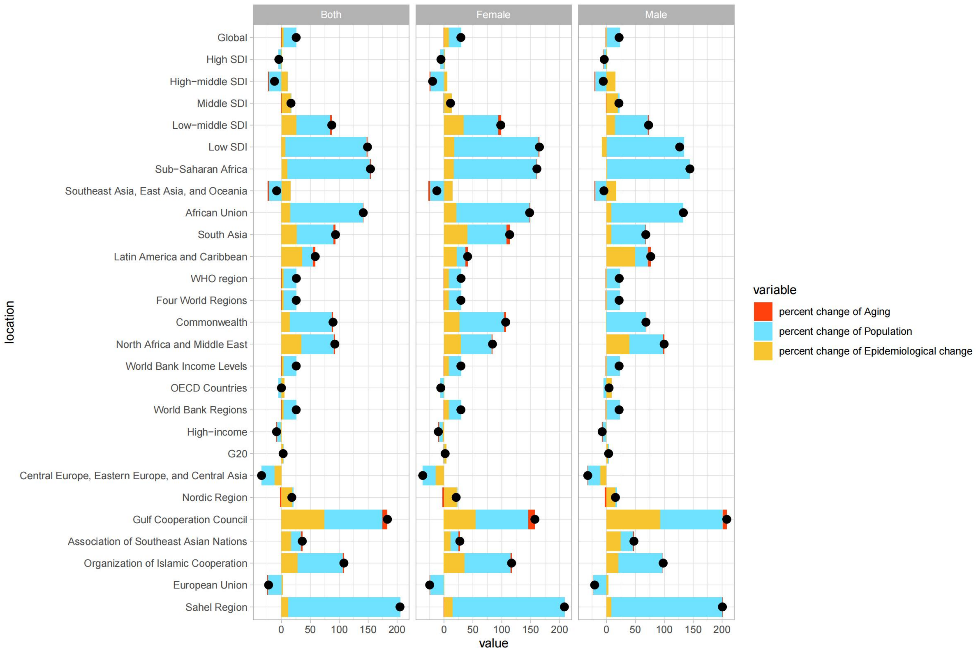

B

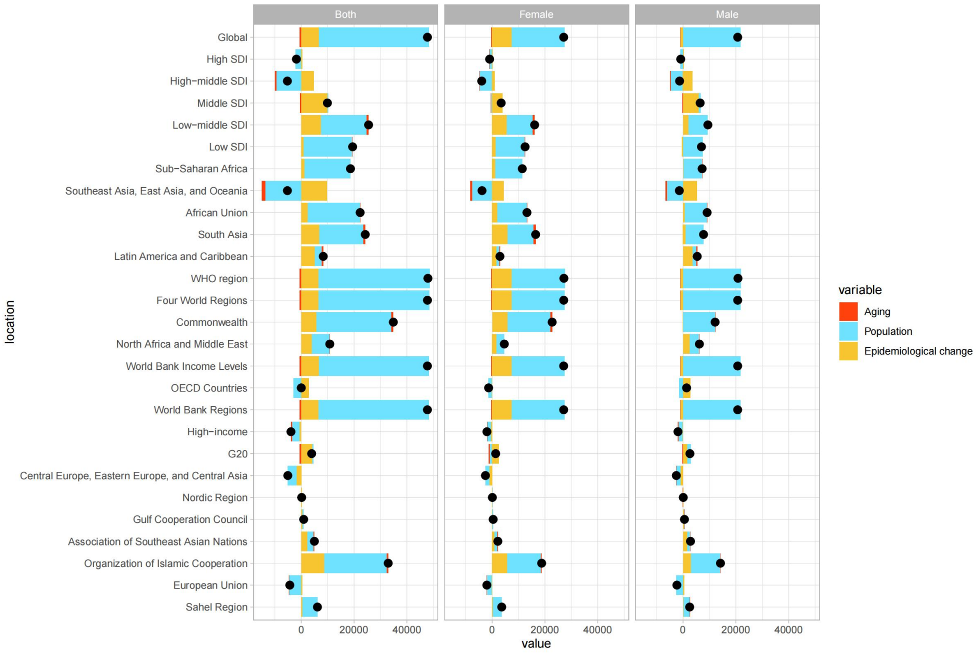

Notes: Supplementary figure 14A represented the gender subgroup decomposition analysis(percentage); Supplementary figure 14B represented the gender subgroup decomposition analysis (Value).

Abbreviations: DALYs, disability-adjusted life years.

Supplementary figure 16. BAPC analysis of total cancers burdens in adolescents and young adults aged 10-24 years.

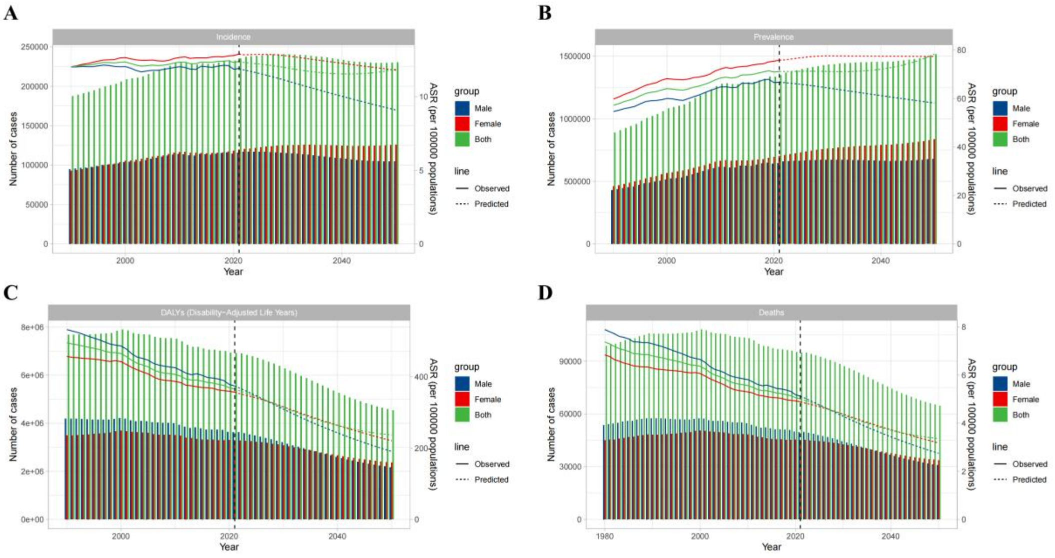

Notes: Figure 10A showed the BAPC prediction results of total cancer incidence; Figure 10B showed the BAPC prediction result of the total cancer prevalence; Figure 10C showed the BAPC prediction results of total cancer DALYs. figure 10D showed BAPC predictions for total cancer deaths.

Supplementary Table 1: AAPC Values of Cancer Disease Burden Among Adolescents Aged 10-24 in the World and 204 Countries/Regions (Incidence)

Measure	Age	Location	Sex	Cause	1990 year Rate (95%UI)	2021 year Rate (95%UI)	AAPC(95%CI)	P Value
Incidence	Age-standardized	Global	Both	Total cancers	12.04(11.12 to 12.81)	12.4(11.35 to 13.35)	0.06(0.02 to 0.09)	0.003
Incidence	Age-standardized	Afghanistan	Both	Total cancers	12.97(7.92 to 18.21)	13.79(9.5 to 17.89)	0.23(0.08 to 0.38)	0.003
Incidence	Age-standardized	Albania	Both	Total cancers	12.6(10.62 to 14.58)	12.62(9.82 to 16)	0.68(0.09 to 1.28)	0.023
Incidence	Age-standardized	Algeria	Both	Total cancers	9.83(7.79 to 12.31)	11.12(8.68 to 13.96)	0.57(0.46 to 0.68)	<0.001
Incidence	Age-standardized	American Samoa	Both	Total cancers	7.58(5.99 to 9.56)	11.44(8.48 to 15.13)	1.46(1.32 to 1.59)	<0.001
Incidence	Age-standardized	Andorra	Both	Total cancers	24.94(18.11 to 34.58)	26.26(18.83 to 35.17)	0.16(-0.07 to 0.39)	0.163
Incidence	Age-standardized	Angola	Both	Total cancers	5.33(3.87 to 7.13)	5.86(4 to 8.11)	0.38(0.03 to 0.72)	0.033
Incidence	Age-standardized	Antigua and Barbuda	Both	Total cancers	9.44(8.46 to 10.54)	11.73(10.48 to 13.12)	0.84(0.17 to 1.51)	0.014
Incidence	Age-standardized	Argentina	Both	Total cancers	15.2(13.71 to 16.79)	17.45(15.48 to 19.75)	0.25(-0.16 to 0.67)	0.229
Incidence	Age-standardized	Armenia	Both	Total cancers	11.87(10.57 to 13.28)	11.77(9.72 to 14.06)	0.35(-0.5 to 1.19)	0.423
Incidence	Age-standardized	Australia	Both	Total cancers	24.41(22.11 to 26.84)	20.76(18.35 to 23.4)	-0.28(-0.67 to 0.11)	0.158
Incidence	Age-standardized	Austria	Both	Total cancers	20.81(18.62 to 23.25)	22.11(19.27 to 25.56)	-0.04(-0.35 to 0.27)	0.781
Incidence	Age-standardized	Azerbaijan	Both	Total cancers	12.47(10.38 to 15.22)	12.22(9.27 to 16.22)	-0.38(-1.02 to 0.26)	0.244
Incidence	Age-standardized	Bahamas	Both	Total cancers	11.68(10.41 to 13.16)	14.22(11.28 to 17.67)	0.76(0.3 to 1.22)	0.001
Incidence	Age-standardized	Bahrain	Both	Total cancers	11.22(9.46 to 13.16)	13.07(10.63 to 16.03)	0.83(-0.32 to 2)	0.157
Incidence	Age-standardized	Bangladesh	Both	Total cancers	9.04(6.98 to 11.49)	11.72(8.59 to 15.77)	0.98(0.78 to 1.18)	<0.001
Incidence	Age-standardized	Barbados	Both	Total cancers	16.93(15.34 to 18.72)	16.31(12.61 to 21.44)	0.3(-0.1 to 0.71)	0.144
Incidence	Age-standardized	Belarus	Both	Total cancers	11.99(10.8 to 13.29)	14.2(11.42 to 17.29)	0.67(0.4 to 0.93)	<0.001
Incidence	Age-standardized	Belgium	Both	Total cancers	22.45(20.17 to 25.24)	21.2(18.39 to 24.46)	-0.36(-0.89 to 0.18)	0.194
Incidence	Age-standardized	Belize	Both	Total cancers	7.64(6.67 to 8.74)	7.48(6.62 to 8.42)	0.2(-0.29 to 0.7)	0.42
Incidence	Age-standardized	Benin	Both	Total cancers	4.75(3.49 to 6.5)	5.16(3.53 to 7.29)	0.17(0.06 to 0.28)	0.003
Incidence	Age-standardized	Bermuda	Both	Total cancers	15.73(13.76 to 18.07)	20.42(16.76 to 25.41)	0.92(0.62 to 1.22)	<0.001
Incidence	Age-standardized	Bhutan	Both	Total cancers	6.45(3.81 to 8.92)	8.35(5.45 to 12.1)	0.89(0.52 to 1.26)	<0.001

Incidence	Age-standardized	Bolivia (Plurinational State of)	Both	Total cancers	13.39(10.83 to 16.21)	13.4(9.54 to 18.89)	-0.02(-0.08 to 0.04)	0.495
Incidence	Age-standardized	Bosnia and Herzegovina	Both	Total cancers	10.17(8.8 to 11.71)	13.24(10.66 to 16.21)	1.2(0.79 to 1.61)	<0.001
Incidence	Age-standardized	Botswana	Both	Total cancers	6.56(4.68 to 8.57)	7.04(5.02 to 9.3)	0.4(-0.16 to 0.95)	0.16
Incidence	Age-standardized	Brazil	Both	Total cancers	10.39(9.64 to 11.15)	13.83(12.74 to 14.88)	0.99(0.81 to 1.17)	<0.001
Incidence	Age-standardized	Brunei Darussalam	Both	Total cancers	14.88(11.95 to 18.19)	10.33(8.65 to 12.34)	-0.98(-1.38 to -0.57)	<0.001
Incidence	Age-standardized	Bulgaria	Both	Total cancers	16.29(14.38 to 18.29)	18.16(14.79 to 21.82)	0.59(0.05 to 1.13)	0.031
Incidence	Age-standardized	Burkina Faso	Both	Total cancers	5.08(3.59 to 7.35)	5.9(3.93 to 8.07)	0.74(0.5 to 0.98)	<0.001
Incidence	Age-standardized	Burundi	Both	Total cancers	12.38(9.47 to 15.98)	10.59(7.8 to 14.08)	-0.64(-0.82 to -0.46)	<0.001
Incidence	Age-standardized	Cabo Verde	Both	Total cancers	5.8(4.35 to 7.63)	8.43(6.31 to 10.84)	1.19(1.03 to 1.36)	<0.001
Incidence	Age-standardized	Cambodia	Both	Total cancers	10.38(7.89 to 13.42)	11.41(7.81 to 16.47)	0.21(0.04 to 0.37)	0.014
Incidence	Age-standardized	Cameroon	Both	Total cancers	5.57(4.18 to 7.5)	6.52(4.21 to 9.19)	0.47(0.26 to 0.68)	<0.001
Incidence	Age-standardized	Canada	Both	Total cancers	25.35(22.37 to 29)	24.5(21.35 to 28.12)	-0.11(-0.33 to 0.1)	0.294
Incidence	Age-standardized	Central African Republic	Both	Total cancers	5.69(4.4 to 7.2)	5.59(4.04 to 7.36)	-0.1(-0.29 to 0.08)	0.272
Incidence	Age-standardized	Chad	Both	Total cancers	3.9(2.71 to 5.45)	5.24(3.67 to 7.39)	1.13(0.96 to 1.29)	<0.001
Incidence	Age-standardized	Chile	Both	Total cancers	11.72(10.6 to 12.92)	19.85(16.6 to 24.02)	2.12(1.82 to 2.42)	<0.001
Incidence	Age-standardized	China	Both	Total cancers	12.58(10.81 to 14.25)	15.69(13.04 to 18.49)	0.58(0.44 to 0.72)	<0.001
Incidence	Age-standardized	Colombia	Both	Total cancers	13.95(12.79 to 15.13)	18.71(15.85 to 21.97)	0.99(0.7 to 1.28)	<0.001
Incidence	Age-standardized	Comoros	Both	Total cancers	11.21(4.47 to 15.98)	12.78(9.18 to 17.2)	0.04(-1.4 to 1.5)	0.956
Incidence	Age-standardized	Congo	Both	Total cancers	5.19(3.79 to 6.86)	6.51(4.83 to 8.32)	0.76(0.54 to 0.97)	<0.001
Incidence	Age-standardized	Cook Islands	Both	Total cancers	5.58(4.12 to 7.5)	7.87(5.46 to 11.59)	1.18(0.96 to 1.39)	<0.001
Incidence	Age-standardized	Costa Rica	Both	Total cancers	13.84(12.57 to 15.1)	22.2(19.39 to 25.46)	1.5(1.24 to 1.75)	<0.001
Incidence	Age-standardized	Coted'Ivoire	Both	Total cancers	4.27(3.16 to 5.8)	5.52(3.72 to 7.82)	1.06(0.95 to 1.18)	<0.001
Incidence	Age-standardized	Croatia	Both	Total cancers	17.15(15.04 to 19.73)	18.58(15.71 to 22.16)	0.32(-0.04 to 0.68)	0.076

Incidence	Age-standardized	Cuba	Both	Total cancers	16.58(15.09 to 18.26)	16.57(14.1 to 19.07)	-0.05(-0.43 to 0.33)	0.795
Incidence	Age-standardized	Cyprus	Both	Total cancers	12.05(9.65 to 14.91)	18.05(13.77 to 23.38)	1.82(1.3 to 2.35)	<0.001
Incidence	Age-standardized	Czechia	Both	Total cancers	16.66(14.98 to 18.75)	21.13(17.32 to 25.52)	0.54(-0.29 to 1.38)	0.202
Incidence	Age-standardized	Democratic People's Republic of Korea	Both	Total cancers	11.1(7.74 to 15.69)	11.48(8.71 to 15.43)	0.12(0.05 to 0.19)	0.001
Incidence	Age-standardized	Democratic Republic of the Congo	Both	Total cancers	4.66(3.47 to 6.1)	4.91(3.55 to 6.44)	0.28(0.12 to 0.43)	<0.001
Incidence	Age-standardized	Denmark	Both	Total cancers	17.75(15.65 to 20.16)	19.68(17.13 to 22.31)	0.1(-0.38 to 0.59)	0.673
Incidence	Age-standardized	Djibouti	Both	Total cancers	7.47(5.15 to 10.23)	9.34(5.86 to 13.91)	0.95(0.45 to 1.46)	<0.001
Incidence	Age-standardized	Dominica	Both	Total cancers	10.41(8.74 to 12.46)	14.12(10.82 to 17.79)	1.18(0.97 to 1.39)	<0.001
Incidence	Age-standardized	Dominican Republic	Both	Total cancers	7.82(6.6 to 9.24)	9.41(7.58 to 11.57)	0.7(0.23 to 1.18)	0.004
Incidence	Age-standardized	Ecuador	Both	Total cancers	10.53(9.46 to 11.61)	15.71(12.84 to 18.82)	1.91(1.51 to 2.32)	<0.001
Incidence	Age-standardized	Egypt	Both	Total cancers	8.11(6.77 to 10.01)	11.39(9.12 to 14.07)	1.31(1.12 to 1.5)	<0.001
Incidence	Age-standardized	El Salvador	Both	Total cancers	10.78(9.38 to 12.42)	15.34(12.04 to 19.53)	1.41(1.23 to 1.59)	<0.001
Incidence	Age-standardized	Equatorial Guinea	Both	Total cancers	5.16(3.81 to 6.93)	6.4(3.58 to 11.35)	0.58(0.36 to 0.79)	<0.001
Incidence	Age-standardized	Eritrea	Both	Total cancers	9.34(7.17 to 11.97)	10.77(7.7 to 14.22)	0.42(0.32 to 0.53)	<0.001
Incidence	Age-standardized	Estonia	Both	Total cancers	19.24(16.53 to 23.03)	33.05(24.76 to 44.67)	1.24(0.4 to 2.08)	0.004
Incidence	Age-standardized	Eswatini	Both	Total cancers	7.07(5.54 to 8.79)	10.09(6.23 to 14.24)	1.43(1.02 to 1.84)	<0.001
Incidence	Age-standardized	Ethiopia	Both	Total cancers	16.28(12.37 to 20.32)	12.72(9.81 to 15.88)	-1.02(-1.22 to -0.82)	<0.001
Incidence	Age-standardized	Fiji	Both	Total cancers	10.84(8.62 to 13.43)	11.58(8.45 to 15.5)	0.33(0.1 to 0.57)	0.006
Incidence	Age-standardized	Finland	Both	Total cancers	16.92(14.92 to 18.95)	25.03(21.83 to 28.59)	1.57(1.13 to 2.01)	<0.001
Incidence	Age-standardized	France	Both	Total cancers	20.79(18.84 to 22.85)	22.54(20.06 to 25.62)	0.26(0.09 to 0.43)	0.003
Incidence	Age-standardized	Gabon	Both	Total cancers	5.17(4 to 6.45)	6.27(4.01 to 8.9)	0.54(0.35 to 0.74)	<0.001
Incidence	Age-standardized	Gambia	Both	Total cancers	5.27(3.57 to 7.36)	7.23(4.66 to 10.21)	0.94(0.42 to 1.47)	<0.001
Incidence	Age-standardized	Georgia	Both	Total cancers	15.52(12.9 to 18.17)	16.79(14.06 to 19.8)	-0.09(-0.9 to 0.72)	0.825
Incidence	Age-standardized	Germany	Both	Total cancers	19.86(17.84 to 21.99)	19(16.64 to 21.74)	-0.14(-0.4 to 0.13)	0.306

Incidence	Age-standardized	Ghana	Both	Total cancers	7.97(5.26 to 11)	6.47(4.55 to 8.91)	-0.97(-1.54 to -0.4)	0.001
Incidence	Age-standardized	Greece	Both	Total cancers	30.82(27.53 to 34.83)	25.51(22.31 to 29.09)	-0.82(-1.1 to -0.53)	<0.001
Incidence	Age-standardized	Greenland	Both	Total cancers	17.39(12.91 to 25.07)	8.5(6.06 to 11.36)	-2.41(-2.6 to -2.23)	<0.001
Incidence	Age-standardized	Grenada	Both	Total cancers	14.38(12.43 to 16.67)	14.09(11.8 to 16.63)	0.19(-0.11 to 0.5)	0.204
Incidence	Age-standardized	Guam	Both	Total cancers	10.54(8.46 to 13.36)	8.5(6.66 to 10.94)	-1.03(-2.75 to 0.73)	0.25
Incidence	Age-standardized	Guatemala	Both	Total cancers	9.4(8.62 to 10.31)	10.75(9.28 to 12.39)	0.54(0.18 to 0.9)	0.003
Incidence	Age-standardized	Guinea	Both	Total cancers	5.91(4.21 to 8.19)	6.98(4.91 to 10.12)	0.59(0.4 to 0.79)	<0.001
Incidence	Age-standardized	Guinea-Bissau	Both	Total cancers	7.22(5.12 to 9.83)	7.31(4.99 to 10.68)	0.09(0 to 0.17)	0.045
Incidence	Age-standardized	Guyana	Both	Total cancers	7.61(5.9 to 9.09)	9.66(7.34 to 12.56)	1.27(0.87 to 1.66)	<0.001
Incidence	Age-standardized	Haiti	Both	Total cancers	13.1(8.94 to 17.49)	12.04(7.9 to 16.77)	0.03(-0.15 to 0.21)	0.728
Incidence	Age-standardized	Honduras	Both	Total cancers	10.21(8.44 to 12.35)	7.67(4.97 to 11.24)	-1.09(-1.3 to -0.88)	<0.001
Incidence	Age-standardized	Hungary	Both	Total cancers	14.95(13.39 to 16.8)	16.83(14.01 to 20.24)	0.55(0.4 to 0.69)	<0.001
Incidence	Age-standardized	Iceland	Both	Total cancers	30.21(25.21 to 36.5)	36.61(29.1 to 46.35)	0.58(0.09 to 1.07)	0.02
Incidence	Age-standardized	India	Both	Total cancers	7.3(6.14 to 8.42)	7.84(6.71 to 9.24)	0.19(0.04 to 0.35)	0.015
Incidence	Age-standardized	Indonesia	Both	Total cancers	8.95(7.22 to 10.91)	10.19(8.01 to 13.17)	0.39(0.31 to 0.46)	<0.001
Incidence	Age-standardized	Iran (Islamic Republic of)	Both	Total cancers	12.14(9.6 to 14.02)	20.3(15.98 to 24.18)	1.95(1.66 to 2.24)	<0.001
Incidence	Age-standardized	Iraq	Both	Total cancers	11.29(8.82 to 14.2)	13.72(10.29 to 18.35)	0.66(0.29 to 1.03)	<0.001
Incidence	Age-standardized	Ireland	Both	Total cancers	20.56(18.25 to 23.22)	19.3(16.49 to 22.4)	0(-0.42 to 0.42)	0.992
Incidence	Age-standardized	Israel	Both	Total cancers	15.97(14.31 to 18)	17.91(15.52 to 20.77)	0.59(0.2 to 0.98)	0.003
Incidence	Age-standardized	Italy	Both	Total cancers	28.48(25.49 to 32.06)	28.09(25.07 to 31.39)	-0.24(-0.62 to 0.14)	0.221
Incidence	Age-standardized	Jamaica	Both	Total cancers	8.98(7.83 to 10.21)	9.73(7.58 to 12.62)	-0.29(-0.83 to 0.25)	0.284
Incidence	Age-standardized	Japan	Both	Total cancers	13.89(12.92 to 15)	15.96(14.61 to 17.36)	0.27(0.14 to 0.41)	<0.001
Incidence	Age-standardized	Jordan	Both	Total cancers	11.29(9.22 to 13.69)	13.93(10.67 to 18.01)	0.6(0.39 to 0.8)	<0.001
Incidence	Age-standardized	Kazakhstan	Both	Total cancers	15.6(14.28 to 16.93)	9.64(8.2 to 11.31)	-1.48(-1.75 to -1.21)	<0.001
Incidence	Age-standardized	Kenya	Both	Total cancers	6.42(4.95 to 7.94)	8.05(6.2 to 10.48)	0.88(0.58 to 1.19)	<0.001

Incidence	Age-standardized	Kiribati	Both	Total cancers	7.07(5.38 to 8.98)	7.82(5.52 to 10.69)	0.31(0.28 to 0.35)	<0.001
Incidence	Age-standardized	Kuwait	Both	Total cancers	14.64(12.8 to 16.69)	13.33(10.81 to 16.36)	-0.38(-0.93 to 0.18)	0.177
Incidence	Age-standardized	Kyrgyzstan	Both	Total cancers	10.51(9.03 to 12.11)	9.54(7.82 to 11.55)	0.08(-0.24 to 0.4)	0.607
Incidence	Age-standardized	Lao People's Democratic Republic	Both	Total cancers	9.88(6.81 to 13.41)	10.63(7.21 to 14.9)	0.2(0.17 to 0.24)	<0.001
Incidence	Age-standardized	Latvia	Both	Total cancers	13.05(11.83 to 14.41)	12.89(11.2 to 14.84)	0.32(-0.35 to 1)	0.348
Incidence	Age-standardized	Lebanon	Both	Total cancers	12.44(9.67 to 16.11)	17.67(13.53 to 22.77)	1.52(1.2 to 1.85)	<0.001
Incidence	Age-standardized	Lesotho	Both	Total cancers	4.97(3.67 to 6.36)	8.87(6.35 to 11.85)	2.5(1.91 to 3.09)	<0.001
Incidence	Age-standardized	Liberia	Both	Total cancers	5.02(3.5 to 6.98)	7.09(4.77 to 9.92)	1.41(1.02 to 1.8)	<0.001
Incidence	Age-standardized	Libya	Both	Total cancers	12.13(9.65 to 15.02)	17.93(13.21 to 23.67)	2.09(1.74 to 2.45)	<0.001
Incidence	Age-standardized	Lithuania	Both	Total cancers	12.93(11.67 to 14.27)	13.32(11.5 to 15.13)	0.49(-0.19 to 1.17)	0.16
Incidence	Age-standardized	Luxembourg	Both	Total cancers	23.94(21.27 to 26.99)	17.97(15.22 to 21.1)	-1.71(-2.28 to -1.12)	<0.001
Incidence	Age-standardized	Madagascar	Both	Total cancers	9.9(7.77 to 12.47)	10.73(7.54 to 14.17)	0.25(0.16 to 0.34)	<0.001
Incidence	Age-standardized	Malawi	Both	Total cancers	12.04(8.8 to 16.43)	14.6(9.33 to 21.21)	0.72(0.63 to 0.82)	<0.001
Incidence	Age-standardized	Malaysia	Both	Total cancers	9.54(7.69 to 11.47)	10.52(8.6 to 12.46)	0.17(-0.21 to 0.56)	0.38
Incidence	Age-standardized	Maldives	Both	Total cancers	6.95(5.19 to 9.18)	6.86(5.51 to 8.55)	0.19(-0.05 to 0.43)	0.115
Incidence	Age-standardized	Mali	Both	Total cancers	6.69(4.9 to 8.89)	6.7(4.77 to 8.86)	0.1(0 to 0.19)	0.049
Incidence	Age-standardized	Malta	Both	Total cancers	22.39(18.61 to 26.83)	42.04(31.77 to 55.86)	1.03(0.08 to 1.99)	0.034
Incidence	Age-standardized	Marshall Islands	Both	Total cancers	6.85(5.43 to 8.7)	8.74(6.33 to 11.86)	0.85(0.56 to 1.15)	<0.001
Incidence	Age-standardized	Mauritania	Both	Total cancers	5.38(3.85 to 7.36)	5.71(4.03 to 8.07)	0.17(0.01 to 0.32)	0.036
Incidence	Age-standardized	Mauritius	Both	Total cancers	7.92(7.29 to 8.57)	10.86(9.59 to 11.95)	0.84(0.36 to 1.33)	0.001
Incidence	Age-standardized	Mexico	Both	Total cancers	11.82(11.22 to 12.48)	17.53(16.15 to 18.96)	1.44(1.32 to 1.56)	<0.001
Incidence	Age-standardized	Micronesia (Federated States of)	Both	Total cancers	7.43(5.83 to 9.46)	8.86(6.15 to 12.29)	0.53(0.43 to 0.62)	<0.001
Incidence	Age-standardized	Monaco	Both	Total cancers	41.33(29.73 to 56.31)	59.12(41.63 to 85.02)	1(0.79 to 1.21)	<0.001
Incidence	Age-standardized	Mongolia	Both	Total cancers	9.97(8.4 to 12.03)	10.85(9.34 to 12.54)	0.12(-0.69 to 0.93)	0.78

Incidence	Age-standardized	Montenegro	Both	Total cancers	18.7(15.47 to 22.43)	22.18(17.28 to 28.58)	0.99(0.68 to 1.31)	<0.001
Incidence	Age-standardized	Morocco	Both	Total cancers	5.29(4.06 to 6.68)	6.14(4.64 to 8.91)	0.41(0.32 to 0.5)	<0.001
Incidence	Age-standardized	Mozambique	Both	Total cancers	8.06(5.9 to 11.06)	10.39(6.85 to 14.78)	0.95(0.76 to 1.15)	<0.001
Incidence	Age-standardized	Myanmar	Both	Total cancers	12.41(8.45 to 16.77)	11.18(8.22 to 15.19)	-0.6(-0.93 to -0.26)	<0.001
Incidence	Age-standardized	Namibia	Both	Total cancers	7.32(5.6 to 9.15)	9.51(6.57 to 14.15)	0.92(0.77 to 1.06)	<0.001
Incidence	Age-standardized	Nauru	Both	Total cancers	8.93(6.78 to 11.32)	11.39(8.01 to 15.42)	0.73(0.65 to 0.82)	<0.001
Incidence	Age-standardized	Nepal	Both	Total cancers	7.27(5.26 to 9.63)	8.22(5.73 to 11.8)	0.49(0.36 to 0.62)	<0.001
Incidence	Age-standardized	Netherlands	Both	Total cancers	18.96(17.1 to 21.05)	20.43(17.83 to 23.31)	0.31(0.09 to 0.52)	0.006
Incidence	Age-standardized	New Zealand	Both	Total cancers	20.83(18.97 to 23.1)	19.58(17.38 to 22.09)	0.12(-0.34 to 0.59)	0.598
Incidence	Age-standardized	Nicaragua	Both	Total cancers	9.38(8.06 to 11.01)	9.59(7.82 to 11.63)	-0.23(-0.79 to 0.34)	0.433
Incidence	Age-standardized	Niger	Both	Total cancers	4.61(3.13 to 6.69)	4.37(2.88 to 6.64)	-0.1(-0.24 to 0.05)	0.202
Incidence	Age-standardized	Nigeria	Both	Total cancers	5.86(3.77 to 8.3)	6.43(3.54 to 10.01)	0.29(0.12 to 0.45)	0.001
Incidence	Age-standardized	Niue	Both	Total cancers	7.09(5.14 to 9.89)	27.02(20.04 to 38.12)	4.39(3.14 to 5.66)	<0.001
Incidence	Age-standardized	North Macedonia	Both	Total cancers	14.41(12.51 to 16.72)	18.68(14.23 to 23.73)	1.15(0.87 to 1.44)	<0.001
Incidence	Age-standardized	Northern Mariana Islands	Both	Total cancers	6.87(5.05 to 9.33)	8.47(6.43 to 11.05)	0.37(-0.36 to 1.1)	0.308
Incidence	Age-standardized	Norway	Both	Total cancers	20.43(18.23 to 23.12)	25.28(22.71 to 27.85)	0.43(-0.17 to 1.03)	0.163
Incidence	Age-standardized	Oman	Both	Total cancers	5.92(4.29 to 7.94)	7.85(5.98 to 10.17)	1.09(-0.55 to 2.76)	0.193
Incidence	Age-standardized	Pakistan	Both	Total cancers	10.6(8.34 to 12.78)	16.95(13.38 to 21.21)	1.4(1.25 to 1.55)	<0.001
Incidence	Age-standardized	Palau	Both	Total cancers	12.45(8.08 to 18.54)	18.51(13.42 to 24.4)	1.15(1 to 1.31)	<0.001
Incidence	Age-standardized	Palestine	Both	Total cancers	11.79(8.86 to 15.38)	14.05(11.51 to 17.66)	0.73(0.08 to 1.38)	0.027
Incidence	Age-standardized	Panama	Both	Total cancers	13.68(12.36 to 15.26)	16.94(14.14 to 20.07)	0.89(0.67 to 1.11)	<0.001
Incidence	Age-standardized	Papua New Guinea	Both	Total cancers	5.84(2.37 to 8.71)	6.98(4.16 to 9.91)	0.43(0.26 to 0.59)	<0.001
Incidence	Age-standardized	Paraguay	Both	Total cancers	8.36(7.01 to 10.06)	11.76(9.17 to 15.23)	1.18(0.97 to 1.38)	<0.001
Incidence	Age-standardized	Peru	Both	Total cancers	13.07(11.15 to 15.36)	16.8(12.94 to 21.52)	1.18(0.91 to 1.45)	<0.001
Incidence	Age-standardized	Philippines	Both	Total cancers	11.01(9.55 to 12.28)	11.38(9.72 to 13.24)	0.12(0.01 to 0.23)	0.04

Incidence	Age-standardized	Poland	Both	Total cancers	13.73(12.93 to 14.68)	15.7(13.95 to 17.66)	0.51(0.31 to 0.72)	<0.001
Incidence	Age-standardized	Portugal	Both	Total cancers	21.84(19.58 to 24.32)	18.05(15.48 to 20.95)	-0.67(-1.21 to -0.11)	0.018
Incidence	Age-standardized	Puerto Rico	Both	Total cancers	12.5(11.24 to 13.86)	16.07(13.13 to 19.49)	0.92(0.47 to 1.37)	<0.001
Incidence	Age-standardized	Qatar	Both	Total cancers	9.31(7.25 to 11.59)	12.09(9.2 to 15.8)	0.8(-0.04 to 1.65)	0.063
Incidence	Age-standardized	Republic of Korea	Both	Total cancers	13.55(10.71 to 15.45)	13.2(10.43 to 15.83)	-0.18(-0.67 to 0.3)	0.458
Incidence	Age-standardized	Republic of Moldova	Both	Total cancers	15.46(14.16 to 16.93)	10.8(9.22 to 12.7)	-0.81(-1.26 to -0.36)	<0.001
Incidence	Age-standardized	Romania	Both	Total cancers	13.31(12.1 to 14.53)	13.72(11.71 to 16.05)	-0.06(-0.44 to 0.33)	0.774
Incidence	Age-standardized	Russian Federation	Both	Total cancers	17.98(17.3 to 18.66)	13.31(12.44 to 14.06)	-1.17(-1.51 to -0.82)	<0.001
Incidence	Age-standardized	Rwanda	Both	Total cancers	14.61(11.76 to 17.95)	11.26(8.05 to 15.41)	-1.21(-1.68 to -0.74)	<0.001
Incidence	Age-standardized	Saint Kitts and Nevis	Both	Total cancers	8.98(8.13 to 9.94)	11.16(9.22 to 13.66)	0.33(-0.18 to 0.85)	0.206
Incidence	Age-standardized	Saint Lucia	Both	Total cancers	10.44(9.49 to 11.41)	13.56(11.18 to 16.48)	0.61(0.29 to 0.93)	<0.001
Incidence	Age-standardized	Saint Vincent and the Grenadines	Both	Total cancers	13.85(12.16 to 15.7)	18.01(15.43 to 21.06)	0.8(0.61 to 0.99)	<0.001
Incidence	Age-standardized	Samoa	Both	Total cancers	9.83(6.98 to 13.48)	12.6(8.9 to 18.1)	0.83(0.72 to 0.94)	<0.001
Incidence	Age-standardized	San Marino	Both	Total cancers	36.32(28.74 to 46.47)	33.37(23.33 to 45.85)	0.3(-0.02 to 0.62)	0.067
Incidence	Age-standardized	Sao Tome and Principe	Both	Total cancers	4.41(2.21 to 7.39)	6.47(4.12 to 10.28)	1.42(0.57 to 2.27)	0.001
Incidence	Age-standardized	Saudi Arabia	Both	Total cancers	6.38(4.66 to 8.45)	10.89(8.16 to 14.4)	1.95(1.65 to 2.25)	<0.001
Incidence	Age-standardized	Senegal	Both	Total cancers	5.42(3.98 to 7.36)	5.96(4.27 to 8.23)	0.52(0.35 to 0.68)	<0.001
Incidence	Age-standardized	Serbia	Both	Total cancers	14.37(11.99 to 17.13)	12.13(9.6 to 15.84)	-0.44(-0.72 to -0.16)	0.002
Incidence	Age-standardized	Seychelles	Both	Total cancers	12.24(10.37 to 14.5)	8.54(6.86 to 10.71)	0.61(0.15 to 1.08)	0.011
Incidence	Age-standardized	Sierra Leone	Both	Total cancers	4.01(2.65 to 6.07)	5.6(3.74 to 8.1)	1.22(0.81 to 1.62)	<0.001
Incidence	Age-standardized	Singapore	Both	Total cancers	10.64(9.75 to 11.58)	20.66(18.21 to 23.47)	1.81(1.15 to 2.48)	<0.001
Incidence	Age-standardized	Slovakia	Both	Total cancers	13.22(11.39 to 15.35)	17.45(13.73 to 22.12)	0.93(0.3 to 1.57)	0.004
Incidence	Age-standardized	Slovenia	Both	Total cancers	16.62(14.86 to 18.59)	21.03(17.36 to 25.87)	1.18(0.78 to 1.58)	<0.001
Incidence	Age-standardized	Solomon Islands	Both	Total cancers	5.28(2.37 to 7.98)	7.3(4.96 to 10.08)	1.08(0.86 to 1.31)	<0.001
Incidence	Age-standardized	Somalia	Both	Total cancers	8.67(6.18 to 11.57)	9.22(6.58 to 12.32)	0.38(0.15 to 0.6)	0.001

Incidence	Age-standardized	South Africa	Both	Total cancers	6.66(5.78 to 7.57)	7.27(6.19 to 8.44)	0.17(-0.35 to 0.7)	0.514
Incidence	Age-standardized	South Sudan	Both	Total cancers	7.49(5.14 to 10.48)	11.44(7.81 to 15.83)	1.06(0.55 to 1.56)	<0.001
Incidence	Age-standardized	Spain	Both	Total cancers	23.27(21.14 to 25.72)	21.27(18.63 to 24.26)	-0.49(-0.73 to -0.26)	<0.001
Incidence	Age-standardized	Sri Lanka	Both	Total cancers	11.43(9.44 to 13.5)	10.82(7.83 to 14.67)	-0.67(-1.22 to -0.11)	0.018
Incidence	Age-standardized	Sudan	Both	Total cancers	8.6(5.86 to 11.62)	11(7.29 to 15.98)	0.86(0.77 to 0.94)	<0.001
Incidence	Age-standardized	Suriname	Both	Total cancers	10.76(6.73 to 12.85)	12.81(10.4 to 15.65)	0.26(-0.37 to 0.91)	0.419
Incidence	Age-standardized	Sweden	Both	Total cancers	19.28(17.1 to 21.82)	20.08(17.51 to 22.8)	0.61(0.23 to 0.99)	0.001
Incidence	Age-standardized	Switzerland	Both	Total cancers	28.44(25.32 to 31.99)	21.02(18.07 to 24.74)	-0.93(-1.22 to -0.64)	<0.001
Incidence	Age-standardized	Syrian Arab Republic	Both	Total cancers	10(7.85 to 12.51)	12.06(9.04 to 15.77)	0.51(0.16 to 0.85)	0.004
Incidence	Age-standardized	Taiwan (Province of China)	Both	Total cancers	14.53(13.5 to 15.79)	17.48(15.42 to 19.57)	0.36(-0.2 to 0.93)	0.204
Incidence	Age-standardized	Tajikistan	Both	Total cancers	10.67(8.97 to 12.53)	9.59(7.55 to 12.04)	-0.79(-1.49 to -0.08)	0.029
Incidence	Age-standardized	Thailand	Both	Total cancers	11.63(9.64 to 13.76)	16.95(13.12 to 21.79)	1.21(0.86 to 1.56)	<0.001
Incidence	Age-standardized	Timor-Leste	Both	Total cancers	6.88(4.82 to 9.38)	8.26(6 to 11.11)	0.77(-0.11 to 1.65)	0.085
Incidence	Age-standardized	Togo	Both	Total cancers	4.91(3.57 to 6.62)	5.69(3.78 to 8.23)	0.55(0.4 to 0.69)	<0.001
Incidence	Age-standardized	Tokelau	Both	Total cancers	7.1(5.1 to 9.39)	27.16(19.74 to 37.36)	4.84(3.34 to 6.37)	<0.001
Incidence	Age-standardized	Tonga	Both	Total cancers	7.73(5.71 to 10.73)	11.87(7.98 to 18.27)	1.42(1.13 to 1.7)	<0.001
Incidence	Age-standardized	Trinidad and Tobago	Both	Total cancers	11.04(10.07 to 12.01)	13.88(10.94 to 17.45)	1.02(0.46 to 1.58)	<0.001
Incidence	Age-standardized	Tunisia	Both	Total cancers	9.29(7.67 to 11.31)	13.41(9.88 to 18.32)	1.16(1.07 to 1.24)	<0.001
Incidence	Age-standardized	Turkey	Both	Total cancers	18.03(14.4 to 22.06)	24.49(18.85 to 31.83)	1.24(0.99 to 1.5)	<0.001
Incidence	Age-standardized	Turkmenistan	Both	Total cancers	10.83(10.03 to 11.72)	16.81(13.4 to 21.25)	1.36(1.19 to 1.52)	<0.001
Incidence	Age-standardized	Tuvalu	Both	Total cancers	6.85(5.31 to 8.6)	7.71(5.71 to 10.19)	0.35(0.32 to 0.39)	<0.001
Incidence	Age-standardized	Uganda	Both	Total cancers	9.65(6.69 to 13.42)	15.24(10.4 to 20.58)	1.2(0.76 to 1.63)	<0.001
Incidence	Age-standardized	Ukraine	Both	Total cancers	17.41(15.59 to 19.38)	12.83(9.83 to 16.16)	-1.36(-1.51 to -1.2)	<0.001
Incidence	Age-standardized	United Arab Emirates	Both	Total cancers	13.79(10.53 to 17.61)	15.57(12.07 to 19.73)	0.42(-1.1 to 1.97)	0.59
Incidence	Age-standardized	United Kingdom	Both	Total cancers	23.44(22.55 to 24.41)	20.79(19.76 to 21.84)	-0.36(-0.68 to -0.03)	0.032

Incidence	Age-standardized	United Republic of Tanzania	Both	Total cancers	10.65(8.21 to 13.78)	11.66(8.12 to 15.6)	0.22(0.13 to 0.31)	<0.001
Incidence	Age-standardized	United States Virgin Islands	Both	Total cancers	12.19(9.85 to 14.91)	16.46(11.9 to 21.64)	1.68(1.31 to 2.04)	<0.001
Incidence	Age-standardized	United States of America	Both	Total cancers	26.96(25.03 to 29.04)	23.72(22.01 to 25.54)	-0.46(-0.61 to -0.3)	<0.001
Incidence	Age-standardized	Uruguay	Both	Total cancers	13.99(12.63 to 15.61)	21.91(18.92 to 25.5)	1.24(1.02 to 1.45)	<0.001
Incidence	Age-standardized	Uzbekistan	Both	Total cancers	10.27(9.11 to 11.64)	10.56(8.79 to 12.51)	0.31(0 to 0.62)	0.052
Incidence	Age-standardized	Vanuatu	Both	Total cancers	5.05(3.41 to 7.24)	6.62(4.72 to 8.74)	0.92(0.43 to 1.42)	<0.001
Incidence	Age-standardized	Venezuela (Bolivarian Republic of)	Both	Total cancers	12.31(11.39 to 13.3)	19.31(14.95 to 24.37)	2.01(1.74 to 2.28)	<0.001
Incidence	Age-standardized	Viet Nam	Both	Total cancers	7.09(5.27 to 9.28)	10.58(7.94 to 13.78)	1.36(1.26 to 1.45)	<0.001
Incidence	Age-standardized	Yemen	Both	Total cancers	4.53(2.3 to 7.15)	5.94(3.23 to 9.35)	0.89(0.57 to 1.21)	<0.001
Incidence	Age-standardized	Zambia	Both	Total cancers	11.64(9.33 to 14.45)	14.76(8.93 to 24.1)	0.65(0.28 to 1.03)	0.001
Incidence	Age-standardized	Zimbabwe	Both	Total cancers	7.01(5.15 to 9.1)	13.47(10.15 to 17.8)	2.71(1.78 to 3.65)	<0.001

Supplementary Table 2: AAPC Values of Cancer Disease Burden Among Adolescents Aged 10-24 in the World and 204 Countries/Regions (Prevalence)

Measure	Age	Location	Sex	Cause	1990 year Rate (95%UI)	2021 year Rate (95%UI)	AAPC(95%CI)	P Value
Prevalence	Age-standardized	Global	Both	Total cancers	57.22(53.81 to 60.13)	71.18(65.68 to 76.34)	0.76(0.66 to 0.85)	<0.001
Prevalence	Age-standardized	Afghanistan	Both	Total cancers	43.68(26 to 62.62)	56.06(37.8 to 74.69)	0.85(0.71 to 0.99)	<0.001
Prevalence	Age-standardized	Albania	Both	Total cancers	59.28(48.21 to 71.16)	81.7(62.07 to 107.59)	1.89(1.26 to 2.51)	<0.001
Prevalence	Age-standardized	Algeria	Both	Total cancers	51.41(40.05 to 65.61)	74.16(56.97 to 95.71)	1.33(1.23 to 1.43)	<0.001
Prevalence	Age-standardized	American Samoa	Both	Total cancers	36.78(28.29 to 48.01)	65.64(46.66 to 90.26)	1.98(1.83 to 2.13)	<0.001
Prevalence	Age-standardized	Andorra	Both	Total cancers	176.18(125.22 to 247.88)	201.13(141.97 to 270.49)	0.42(0.18 to 0.67)	0.001
Prevalence	Age-standardized	Angola	Both	Total cancers	21.29(15.04 to 29.52)	28.22(18.27 to 39.89)	0.94(0.59 to 1.29)	<0.001
Prevalence	Age-standardized	Antigua and Barbuda	Both	Total cancers	50.56(45.15 to 57.11)	70.2(61.62 to 79.85)	1.17(0.53 to 1.82)	<0.001
Prevalence	Age-standardized	Argentina	Both	Total cancers	82.3(71.91 to 94.06)	115.21(98.82 to 133.99)	0.88(0.47 to 1.3)	<0.001
Prevalence	Age-standardized	Armenia	Both	Total cancers	52.11(44.34 to 60.6)	65.83(53.1 to 81.04)	1.26(0.32 to 2.22)	0.009
Prevalence	Age-standardized	Australia	Both	Total cancers	173.05(154.6 to 193.43)	154.7(135.62 to 176.95)	-0.01(-0.5 to 0.47)	0.955
Prevalence	Age-standardized	Austria	Both	Total cancers	148.66(131.64 to 167.18)	173.64(149.81 to 203.4)	0.26(-0.05 to 0.58)	0.101
Prevalence	Age-standardized	Azerbaijan	Both	Total cancers	48.2(38.07 to 62.04)	58.21(41.56 to 82.36)	0.36(-0.34 to 1.06)	0.314
Prevalence	Age-standardized	Bahamas	Both	Total cancers	63.72(55.7 to 73.4)	83.78(65.66 to 104.16)	1.07(0.64 to 1.5)	<0.001
Prevalence	Age-standardized	Bahrain	Both	Total cancers	61.97(51.39 to 74.02)	92.21(73.67 to 114.48)	1.75(0.44 to 3.07)	0.009
Prevalence	Age-standardized	Bangladesh	Both	Total cancers	37.2(28.73 to 48.49)	66.49(47.69 to 92.33)	2.03(1.83 to 2.23)	<0.001
Prevalence	Age-standardized	Barbados	Both	Total cancers	93.84(84.3 to 104.42)	102.18(79.1 to 133.97)	0.72(0.29 to 1.15)	0.001
Prevalence	Age-standardized	Belarus	Both	Total cancers	64.97(56.92 to 74.01)	96.01(75.96 to 119.87)	1.47(1.19 to 1.75)	<0.001
Prevalence	Age-standardized	Belgium	Both	Total cancers	155.56(137.19 to 177.85)	161(138.61 to 187.86)	0(-0.51 to 0.52)	0.986

Prevalence	Age-standardized	Belize	Both	Total cancers	37.55(32.44 to 42.85)	38.48(33.73 to 43.56)	0.1(-0.47 to 0.68)	0.728
Prevalence	Age-standardized	Benin	Both	Total cancers	20.83(14.67 to 30.26)	25.42(16.86 to 37.02)	0.53(0.41 to 0.64)	<0.001
Prevalence	Age-standardized	Bermuda	Both	Total cancers	95.52(82.86 to 110.77)	147.82(120.26 to 185.13)	1.53(1.24 to 1.82)	<0.001
Prevalence	Age-standardized	Bhutan	Both	Total cancers	26.41(15.61 to 37.33)	45.29(29.24 to 67.81)	1.85(1.46 to 2.24)	<0.001
Prevalence	Age-standardized	Bolivia (Plurinational State of)	Both	Total cancers	49.5(39.03 to 61.57)	60.77(42.26 to 86.62)	0.64(0.58 to 0.71)	<0.001
Prevalence	Age-standardized	Bosnia and Herzegovina	Both	Total cancers	48.93(41.12 to 57.64)	83.81(66.32 to 104.69)	2.2(1.76 to 2.65)	<0.001
Prevalence	Age-standardized	Botswana	Both	Total cancers	27.91(19.12 to 37.9)	34.39(23.51 to 46.18)	0.86(0.25 to 1.48)	0.006
Prevalence	Age-standardized	Brazil	Both	Total cancers	41.04(38.63 to 43.5)	68.13(63.26 to 73.11)	1.64(1.41 to 1.87)	<0.001
Prevalence	Age-standardized	Brunei Darussalam	Both	Total cancers	69.44(55.53 to 85.87)	59.6(49.39 to 71.51)	-0.32(-0.7 to 0.05)	0.089
Prevalence	Age-standardized	Bulgaria	Both	Total cancers	81.16(68.2 to 96.43)	110.92(83.31 to 140.9)	1.13(0.65 to 1.62)	<0.001
Prevalence	Age-standardized	Burkina Faso	Both	Total cancers	23.53(15.87 to 34.36)	30.08(19.82 to 42.98)	1.08(0.84 to 1.32)	<0.001
Prevalence	Age-standardized	Burundi	Both	Total cancers	58.51(42.82 to 77.46)	53.85(39.12 to 73.81)	-0.39(-0.56 to -0.22)	<0.001
Prevalence	Age-standardized	Cabo Verde	Both	Total cancers	24.45(17.61 to 34.14)	43.89(30.53 to 58.55)	1.96(1.78 to 2.15)	<0.001
Prevalence	Age-standardized	Cambodia	Both	Total cancers	37.83(29.12 to 49.54)	56.01(37.35 to 82.38)	1.21(1.06 to 1.36)	<0.001
Prevalence	Age-standardized	Cameroon	Both	Total cancers	26(18.54 to 36.82)	33.53(20.79 to 48.22)	0.83(0.69 to 0.96)	<0.001
Prevalence	Age-standardized	Canada	Both	Total cancers	189.68(165.56 to 219.83)	191.83(165.87 to 222.51)	0.05(-0.18 to 0.27)	0.689
Prevalence	Age-standardized	Central African Republic	Both	Total cancers	22.38(16.65 to 30.1)	23.32(16.09 to 31.74)	0.07(-0.13 to 0.27)	0.481
Prevalence	Age-standardized	Chad	Both	Total cancers	16.64(11.3 to 24.39)	23.93(15.84 to 35.87)	1.37(1.28 to 1.46)	<0.001
Prevalence	Age-standardized	Chile	Both	Total cancers	61.13(49.99 to 72.53)	146.57(118.81 to 183.09)	3.35(3 to 3.7)	<0.001

Prevalence	Age-standardized	China	Both	Total cancers	47.3(40.96 to 53.52)	93.06(77.77 to 109.85)	2.35(2.18 to 2.52)	<0.001
Prevalence	Age-standardized	Colombia	Both	Total cancers	58.89(53.79 to 64.75)	100.59(81.3 to 124.56)	1.79(1.5 to 2.09)	<0.001
Prevalence	Age-standardized	Comoros	Both	Total cancers	55.66(22.23 to 81.81)	69.39(47.67 to 96.56)	0.28(-1.23 to 1.82)	0.718
Prevalence	Age-standardized	Congo	Both	Total cancers	21.49(15.24 to 29.33)	32.66(23.4 to 43.36)	1.39(1.17 to 1.61)	<0.001
Prevalence	Age-standardized	Cook Islands	Both	Total cancers	32.09(22.2 to 45.65)	56.78(37.65 to 87.14)	1.87(1.49 to 2.26)	<0.001
Prevalence	Age-standardized	Costa Rica	Both	Total cancers	68.62(61.19 to 77.06)	125.07(102.57 to 152.15)	1.87(1.6 to 2.14)	<0.001
Prevalence	Age-standardized	Coted'Ivoire	Both	Total cancers	20.56(14.66 to 28.89)	30.62(19.98 to 44.84)	1.43(1.18 to 1.68)	<0.001
Prevalence	Age-standardized	Croatia	Both	Total cancers	113.08(97.46 to 131.91)	137.98(115.78 to 166.83)	0.78(0.39 to 1.17)	<0.001
Prevalence	Age-standardized	Cuba	Both	Total cancers	91.92(82.08 to 103.26)	105.45(88.66 to 123.84)	0.36(-0.02 to 0.74)	0.063
Prevalence	Age-standardized	Cyprus	Both	Total cancers	78.35(61.96 to 101.01)	142.48(107.19 to 187.26)	2.46(1.91 to 3.02)	<0.001
Prevalence	Age-standardized	Czechia	Both	Total cancers	100.23(86.36 to 116.26)	157.84(126.65 to 196.29)	1.05(0.02 to 2.09)	0.046
Prevalence	Age-standardized	Democratic People's Republic of Korea	Both	Total cancers	46.6(32.54 to 66.18)	57.01(42.82 to 77.6)	0.63(0.43 to 0.82)	<0.001
Prevalence	Age-standardized	Democratic Republic of the Congo	Both	Total cancers	19.91(14.06 to 27.6)	23.17(15.7 to 31.72)	0.59(0.43 to 0.76)	<0.001
Prevalence	Age-standardized	Denmark	Both	Total cancers	122.59(105.76 to 141.67)	153.17(131.96 to 174.92)	0.51(0.01 to 1.02)	0.044
Prevalence	Age-standardized	Djibouti	Both	Total cancers	36.44(24.25 to 51.57)	50.03(30.29 to 76.68)	1.36(0.78 to 1.95)	<0.001
Prevalence	Age-standardized	Dominica	Both	Total cancers	50.2(40.95 to 61.82)	74.98(57.03 to 96.04)	1.46(1.31 to 1.61)	<0.001
Prevalence	Age-standardized	Dominican Republic	Both	Total cancers	35.24(28.89 to 42.46)	48.32(38.48 to 60.32)	1.09(0.67 to 1.51)	<0.001

Prevalence	Age-standardized	Ecuador	Both	Total cancers	44.65(39.26 to 50.69)	79.22(62.97 to 99.49)	2.48(2.07 to 2.88)	<0.001
Prevalence	Age-standardized	Egypt	Both	Total cancers	34.99(28.68 to 43.51)	60.24(47.04 to 76.29)	1.79(1.61 to 1.97)	<0.001
Prevalence	Age-standardized	El Salvador	Both	Total cancers	39.23(34.07 to 45.31)	74.22(57 to 95.58)	2.36(1.98 to 2.74)	<0.001
Prevalence	Age-standardized	Equatorial Guinea	Both	Total cancers	21.22(15.3 to 29.6)	35.12(19.05 to 64.3)	1.62(1.36 to 1.87)	<0.001
Prevalence	Age-standardized	Eritrea	Both	Total cancers	42.88(31.24 to 56.32)	53.84(36.71 to 73.91)	0.66(0.55 to 0.78)	<0.001
Prevalence	Age-standardized	Estonia	Both	Total cancers	118.28(97.25 to 149.19)	258.77(186.39 to 360.41)	1.95(1.03 to 2.87)	<0.001
Prevalence	Age-standardized	Eswatini	Both	Total cancers	30.87(23.31 to 40.41)	46.49(27.21 to 67.51)	1.51(1.21 to 1.81)	<0.001
Prevalence	Age-standardized	Ethiopia	Both	Total cancers	68.19(53.12 to 83.84)	65.38(49.42 to 84.45)	-0.32(-0.53 to -0.11)	0.003
Prevalence	Age-standardized	Fiji	Both	Total cancers	50.44(39.25 to 63.79)	61.36(44.39 to 83.7)	0.63(0.41 to 0.85)	<0.001
Prevalence	Age-standardized	Finland	Both	Total cancers	116.38(101.26 to 132.96)	195.65(168.57 to 226.27)	2.02(1.57 to 2.48)	<0.001
Prevalence	Age-standardized	France	Both	Total cancers	141.67(126.2 to 159.48)	171.25(150.48 to 196.4)	0.65(0.47 to 0.83)	<0.001
Prevalence	Age-standardized	Gabon	Both	Total cancers	22.65(16.73 to 28.89)	33.1(20.76 to 47.8)	1.14(0.89 to 1.39)	<0.001
Prevalence	Age-standardized	Gambia	Both	Total cancers	24.44(15.58 to 35.07)	36.83(22.3 to 54.21)	1.3(0.77 to 1.82)	<0.001
Prevalence	Age-standardized	Georgia	Both	Total cancers	81.84(64.93 to 101.47)	98.06(80.29 to 118.94)	0.15(-0.71 to 1.03)	0.731
Prevalence	Age-standardized	Germany	Both	Total cancers	139.97(123.97 to 158.59)	147.49(127.55 to 170.63)	0.26(-0.02 to 0.54)	0.069
Prevalence	Age-standardized	Ghana	Both	Total cancers	34.63(23.02 to 49.83)	33(22.02 to 46.88)	-0.45(-0.98 to 0.09)	0.105
Prevalence	Age-standardized	Greece	Both	Total cancers	223.84(197.39 to 255.69)	191.25(166.01 to 219.5)	-0.67(-0.97 to -0.38)	<0.001
Prevalence	Age-standardized	Greenland	Both	Total cancers	87.38(62.81 to 127.77)	48.72(33.52 to 67.63)	-2.03(-2.26 to -1.8)	<0.001

Prevalence	Age-standardized	Grenada	Both	Total cancers	72.65(61.36 to 85.52)	83.03(68.15 to 100.63)	0.49(0.28 to 0.69)	<0.001
Prevalence	Age-standardized	Guam	Both	Total cancers	60.21(45.93 to 81)	51.42(38.76 to 69.05)	-0.91(-2.64 to 0.86)	0.312
Prevalence	Age-standardized	Guatemala	Both	Total cancers	34.22(31.13 to 38.3)	41.33(35.45 to 47.99)	0.74(0.34 to 1.14)	<0.001
Prevalence	Age-standardized	Guinea	Both	Total cancers	26.08(17.87 to 37.67)	34.2(23.13 to 51.4)	1(0.93 to 1.08)	<0.001
Prevalence	Age-standardized	Guinea-Bissau	Both	Total cancers	29.72(20.34 to 42.13)	33.45(21.24 to 51.21)	0.44(0.34 to 0.54)	<0.001
Prevalence	Age-standardized	Guyana	Both	Total cancers	33.63(25.38 to 41.38)	48.84(36.38 to 64.46)	1.53(1.11 to 1.95)	<0.001
Prevalence	Age-standardized	Haiti	Both	Total cancers	48.51(33.9 to 64.76)	50.43(32.61 to 69.79)	0.44(0.25 to 0.64)	<0.001
Prevalence	Age-standardized	Honduras	Both	Total cancers	34.53(27.87 to 42.81)	30.48(18.39 to 47.12)	-0.57(-0.79 to -0.34)	<0.001
Prevalence	Age-standardized	Hungary	Both	Total cancers	85.19(69.18 to 100.68)	121.35(98.14 to 150.04)	1.23(0.95 to 1.51)	<0.001
Prevalence	Age-standardized	Iceland	Both	Total cancers	227.16(187.35 to 279.9)	294.66(229.59 to 380.17)	0.83(0.32 to 1.34)	0.001
Prevalence	Age-standardized	India	Both	Total cancers	31.2(26.06 to 36.41)	41.97(35.57 to 50.14)	0.96(0.78 to 1.13)	<0.001
Prevalence	Age-standardized	Indonesia	Both	Total cancers	35.81(28.75 to 45.52)	49.89(38.85 to 66.14)	1.1(1 to 1.2)	<0.001
Prevalence	Age-standardized	Iran (Islamic Republic of)	Both	Total cancers	52.51(43 to 60.85)	130.08(101.95 to 155.3)	3.25(2.89 to 3.61)	<0.001
Prevalence	Age-standardized	Iraq	Both	Total cancers	51.66(39.47 to 65.76)	82.99(61.25 to 113.27)	1.81(1.5 to 2.13)	<0.001
Prevalence	Age-standardized	Ireland	Both	Total cancers	138.98(121.31 to 159.46)	147.39(124.9 to 172.6)	0.53(0.11 to 0.96)	0.014
Prevalence	Age-standardized	Israel	Both	Total cancers	101.6(89.52 to 116.38)	133.29(114.46 to 156.85)	1.07(0.66 to 1.47)	<0.001
Prevalence	Age-standardized	Italy	Both	Total cancers	194.41(171.01 to 222.68)	215.29(189.26 to 243.98)	0.16(-0.25 to 0.57)	0.448
Prevalence	Age-standardized	Jamaica	Both	Total cancers	50.5(43.14 to 58.2)	59.04(45.69 to 77.45)	0.04(-0.47 to 0.56)	0.861
Prevalence	Age-standardized	Japan	Both	Total cancers	90.51(82.77 to 99.14)	119.8(109.22 to 130.61)	0.87(0.55 to 1.19)	<0.001
Prevalence	Age-standardized	Jordan	Both	Total cancers	56.76(45.06 to 70.81)	96.2(71.94 to 128.67)	1.72(1.53 to 1.9)	<0.001

Prevalence	Age-standardized	Kazakhstan	Both	Total cancers	72.64(65.86 to 80.57)	54.9(45.55 to 65.91)	-0.94(-1.1 to -0.78)	<0.001
Prevalence	Age-standardized	Kenya	Both	Total cancers	32.77(24.96 to 41.09)	44.4(33.91 to 59.33)	1.05(0.83 to 1.27)	<0.001
Prevalence	Age-standardized	Kiribati	Both	Total cancers	25.97(19.61 to 34.1)	32.49(22.68 to 45.59)	0.69(0.63 to 0.74)	<0.001
Prevalence	Age-standardized	Kuwait	Both	Total cancers	97.79(83.93 to 113.59)	101.4(81.49 to 125.99)	0.12(-0.51 to 0.77)	0.694
Prevalence	Age-standardized	Kyrgyzstan	Both	Total cancers	47.51(39.18 to 56.76)	51.9(41.34 to 65.03)	0.73(0.38 to 1.08)	<0.001
Prevalence	Age-standardized	Lao People's Democratic Republic	Both	Total cancers	32.86(22.64 to 45.98)	48.33(31.88 to 71.09)	1.25(1.2 to 1.3)	<0.001
Prevalence	Age-standardized	Latvia	Both	Total cancers	67.43(58.86 to 77.05)	82.54(69.28 to 96.77)	0.92(0.14 to 1.7)	0.02
Prevalence	Age-standardized	Lebanon	Both	Total cancers	69.32(53.01 to 90.3)	128.77(97.54 to 168.64)	2.29(1.78 to 2.81)	<0.001
Prevalence	Age-standardized	Lesotho	Both	Total cancers	20.43(14.51 to 27.31)	38.26(26.41 to 52.89)	2.75(2.44 to 3.06)	<0.001
Prevalence	Age-standardized	Liberia	Both	Total cancers	22.36(15.26 to 32.47)	36.67(23.75 to 54.04)	1.95(1.54 to 2.36)	<0.001
Prevalence	Age-standardized	Libya	Both	Total cancers	61.4(48.09 to 77.72)	112.96(80.95 to 150.92)	2.3(1.39 to 3.21)	<0.001
Prevalence	Age-standardized	Lithuania	Both	Total cancers	72.64(64.19 to 81.71)	87.4(74.16 to 101.69)	1(0.26 to 1.74)	0.008
Prevalence	Age-standardized	Luxembourg	Both	Total cancers	158.85(139.44 to 181.63)	137.19(114.95 to 162.13)	-1.27(-1.88 to -0.67)	<0.001
Prevalence	Age-standardized	Madagascar	Both	Total cancers	48(35.76 to 62.62)	56.57(38.29 to 77.54)	0.52(0.42 to 0.61)	<0.001
Prevalence	Age-standardized	Malawi	Both	Total cancers	64.68(45.61 to 91.38)	86.61(53 to 130.67)	1.1(1 to 1.21)	<0.001
Prevalence	Age-standardized	Malaysia	Both	Total cancers	40.3(32.72 to 48.46)	59.69(48.51 to 71.97)	1.1(0.87 to 1.32)	<0.001
Prevalence	Age-standardized	Maldives	Both	Total cancers	28.81(21.8 to 40.52)	42(33.76 to 52.61)	1.51(1.26 to 1.76)	<0.001
Prevalence	Age-standardized	Mali	Both	Total cancers	29.63(20.82 to 41.95)	32.35(22.22 to 45.41)	0.38(0.28 to 0.48)	<0.001
Prevalence	Age-standardized	Malta	Both	Total cancers	158.09(128.88 to 195.43)	336.32(247.5 to 454.66)	1.39(0.39 to 2.4)	0.006
Prevalence	Age-standardized	Marshall Islands	Both	Total cancers	24.28(18.82 to 31.44)	39.69(27.85 to 56.46)	1.52(1.39 to 1.65)	<0.001
Prevalence	Age-standardized	Mauritania	Both	Total cancers	22.79(16.44 to 31.27)	30.67(20.79 to 44.83)	0.94(0.74 to 1.13)	<0.001
Prevalence	Age-standardized	Mauritius	Both	Total cancers	40.56(36.9 to 44.43)	68.97(60.01 to 77.16)	1.54(1.03 to 2.06)	<0.001

Prevalence	Age-standardized	Mexico	Both	Total cancers	45.45(43.62 to 47.49)	74.41(67.16 to 81.47)	1.75(1.69 to 1.8)	<0.001
Prevalence	Age-standardized	Micronesia (Federated States of)	Both	Total cancers	27.81(20.81 to 36.93)	41.92(27.63 to 59.96)	1.27(1.17 to 1.36)	<0.001
Prevalence	Age-standardized	Monaco	Both	Total cancers	316.03(221.87 to 440.67)	477.23(329.74 to 700.95)	1.19(0.98 to 1.4)	<0.001
Prevalence	Age-standardized	Mongolia	Both	Total cancers	34.03(28.17 to 41.24)	47.89(40.56 to 56.46)	1.01(0.23 to 1.8)	0.011
Prevalence	Age-standardized	Montenegro	Both	Total cancers	119.62(97.54 to 145.2)	160.58(121.26 to 210.8)	1.5(1.18 to 1.81)	<0.001
Prevalence	Age-standardized	Morocco	Both	Total cancers	26.53(19.87 to 34.17)	38.66(28.63 to 55.94)	1.13(1.05 to 1.22)	<0.001
Prevalence	Age-standardized	Mozambique	Both	Total cancers	40.46(28.29 to 57.31)	56.09(35.94 to 81.67)	1.25(1.16 to 1.35)	<0.001
Prevalence	Age-standardized	Myanmar	Both	Total cancers	46.75(32.52 to 64.67)	55.5(40.11 to 76)	0.36(0.06 to 0.66)	0.02
Prevalence	Age-standardized	Namibia	Both	Total cancers	34.02(24.91 to 45.08)	52.58(34.51 to 81.27)	1.48(1.39 to 1.57)	<0.001
Prevalence	Age-standardized	Nauru	Both	Total cancers	36(26.17 to 48.16)	55.4(37.54 to 78.26)	1.34(1.24 to 1.44)	<0.001
Prevalence	Age-standardized	Nepal	Both	Total cancers	29.69(20.85 to 40.92)	43.33(29.62 to 63.82)	1.35(1.2 to 1.51)	<0.001
Prevalence	Age-standardized	Netherlands	Both	Total cancers	134.08(119.64 to 149.76)	159.9(138.02 to 183.51)	0.64(0.42 to 0.87)	<0.001
Prevalence	Age-standardized	New Zealand	Both	Total cancers	132.13(118.26 to 147.85)	137.95(120.46 to 159.08)	0.43(-0.07 to 0.94)	0.092
Prevalence	Age-standardized	Nicaragua	Both	Total cancers	37.63(31.39 to 45.35)	44.42(36.09 to 55.09)	0.2(-0.45 to 0.85)	0.554
Prevalence	Age-standardized	Niger	Both	Total cancers	19.92(13.07 to 30.41)	20.53(12.99 to 32.26)	0.17(0.02 to 0.32)	0.029
Prevalence	Age-standardized	Nigeria	Both	Total cancers	25.88(16.73 to 36.75)	32.44(18.09 to 50.9)	0.74(0.55 to 0.94)	<0.001
Prevalence	Age-standardized	Niue	Both	Total cancers	33.6(23.28 to 48.83)	171.61(119.22 to 259.37)	5.71(4.99 to 6.44)	<0.001
Prevalence	Age-standardized	North Macedonia	Both	Total cancers	73.25(60.69 to 87.22)	124.7(91.24 to 162.1)	2.09(1.79 to 2.39)	<0.001
Prevalence	Age-standardized	Northern Mariana Islands	Both	Total cancers	34.86(24.2 to 49.79)	51.23(37.32 to 70.77)	0.79(-0.14 to 1.72)	0.094
Prevalence	Age-standardized	Norway	Both	Total cancers	148.25(130.51 to 169.18)	196.47(175.52 to 219.1)	0.67(0.05 to 1.3)	0.034
Prevalence	Age-standardized	Oman	Both	Total cancers	29.43(20.95 to 40.03)	53.59(39.66 to 70.88)	2(0.04 to 4)	0.046

Prevalence	Age-standardized	Pakistan	Both	Total cancers	45(34.95 to 55.58)	85(64.64 to 109.59)	2.11(1.93 to 2.3)	<0.001
Prevalence	Age-standardized	Palau	Both	Total cancers	65.91(40.93 to 102.02)	111.1(79.09 to 148.68)	1.51(1.31 to 1.71)	<0.001
Prevalence	Age-standardized	Palestine	Both	Total cancers	57.79(42.62 to 76.05)	89.91(72.11 to 115.16)	1.64(0.95 to 2.33)	<0.001
Prevalence	Age-standardized	Panama	Both	Total cancers	64.86(56.94 to 73.98)	90.97(73.47 to 112.68)	1.18(0.77 to 1.59)	<0.001
Prevalence	Age-standardized	Papua New Guinea	Both	Total cancers	22.56(9.34 to 35.09)	31.15(17.98 to 45.73)	0.81(0.63 to 1)	<0.001
Prevalence	Age-standardized	Paraguay	Both	Total cancers	40.65(33.24 to 50.05)	64.6(49.08 to 85.28)	1.75(1.38 to 2.12)	<0.001
Prevalence	Age-standardized	Peru	Both	Total cancers	53.6(44.86 to 64.45)	97.39(72.46 to 126.43)	2.43(2.17 to 2.7)	<0.001
Prevalence	Age-standardized	Philippines	Both	Total cancers	48.67(42.24 to 55.4)	57.67(48.14 to 68.95)	0.51(0.41 to 0.61)	<0.001
Prevalence	Age-standardized	Poland	Both	Total cancers	71.8(63.71 to 79.45)	108.34(93.63 to 125.47)	1.4(1.16 to 1.63)	<0.001
Prevalence	Age-standardized	Portugal	Both	Total cancers	135.04(118.2 to 153.67)	135.4(114.48 to 160.66)	0.07(-0.48 to 0.63)	0.795
Prevalence	Age-standardized	Puerto Rico	Both	Total cancers	68.49(60.68 to 77.69)	111.24(88.54 to 137.03)	1.26(0.85 to 1.67)	<0.001
Prevalence	Age-standardized	Qatar	Both	Total cancers	50.02(38.84 to 63.31)	86.65(65.39 to 114.25)	1.73(0.8 to 2.67)	<0.001
Prevalence	Age-standardized	Republic of Korea	Both	Total cancers	61.52(50.13 to 70.91)	95.56(75.13 to 115.32)	1.5(0.95 to 2.05)	<0.001
Prevalence	Age-standardized	Republic of Moldova	Both	Total cancers	81.01(71.34 to 91.47)	68.43(57.46 to 81.98)	-0.28(-0.79 to 0.24)	0.297
Prevalence	Age-standardized	Romania	Both	Total cancers	68.22(59.2 to 77.7)	91.04(76.35 to 108.47)	0.86(0.57 to 1.16)	<0.001
Prevalence	Age-standardized	Russian Federation	Both	Total cancers	107.86(103.55 to 112.25)	89.77(83.72 to 95.11)	-0.96(-1.33 to -0.59)	<0.001
Prevalence	Age-standardized	Rwanda	Both	Total cancers	68.3(53.35 to 85.42)	60.66(41.8 to 85.1)	-0.59(-1.15 to -0.02)	0.044
Prevalence	Age-standardized	Saint Kitts and Nevis	Both	Total cancers	43.09(38.35 to 48.33)	62.41(51.01 to 77.15)	0.88(0.42 to 1.33)	<0.001
Prevalence	Age-standardized	Saint Lucia	Both	Total cancers	51.09(45.52 to 56.62)	77.65(62.82 to 96.03)	1(0.63 to 1.38)	<0.001
Prevalence	Age-standardized	Saint Vincent and the Grenadines	Both	Total cancers	74.27(64.02 to 86.27)	104.66(88.1 to 123.68)	1.03(0.83 to 1.23)	<0.001
Prevalence	Age-standardized	Samoa	Both	Total cancers	48.73(33.58 to 68.43)	75.55(51.14 to 112.12)	1.47(1.39 to 1.55)	<0.001

Prevalence	Age-standardized	San Marino	Both	Total cancers	274.11(213.62 to 354.62)	263.65(181.96 to 364.09)	0.45(0.12 to 0.79)	0.008
Prevalence	Age-standardized	Sao Tome and Principe	Both	Total cancers	23.24(10.95 to 40.01)	39.14(24.07 to 64.45)	1.88(0.99 to 2.79)	<0.001
Prevalence	Age-standardized	Saudi Arabia	Both	Total cancers	33.22(23.5 to 44.79)	75.35(55.44 to 100.27)	2.92(2.55 to 3.29)	<0.001
Prevalence	Age-standardized	Senegal	Both	Total cancers	24.62(16.96 to 36.21)	30.05(20.32 to 44.21)	0.82(0.65 to 0.99)	<0.001
Prevalence	Age-standardized	Serbia	Both	Total cancers	79.64(63.72 to 100.47)	85.14(65.03 to 113.76)	0.41(0.13 to 0.69)	0.004
Prevalence	Age-standardized	Seychelles	Both	Total cancers	59.96(49.82 to 73.13)	54.26(42.92 to 68.92)	1.12(0.66 to 1.58)	<0.001
Prevalence	Age-standardized	Sierra Leone	Both	Total cancers	17.65(11.31 to 27.32)	28.15(18.18 to 42.26)	1.65(1.3 to 1.99)	<0.001
Prevalence	Age-standardized	Singapore	Both	Total cancers	58.7(53.18 to 64.8)	150.85(131.91 to 172.44)	2.85(2.12 to 3.59)	<0.001
Prevalence	Age-standardized	Slovakia	Both	Total cancers	78.02(64.96 to 93.59)	126.1(95.83 to 166.1)	1.51(0.95 to 2.08)	<0.001
Prevalence	Age-standardized	Slovenia	Both	Total cancers	108.64(95.32 to 124.75)	165.97(134.9 to 207.49)	1.84(1.4 to 2.29)	<0.001
Prevalence	Age-standardized	Solomon Islands	Both	Total cancers	18.65(8.28 to 29.29)	31.77(21.1 to 45.84)	1.69(1.42 to 1.97)	<0.001
Prevalence	Age-standardized	Somalia	Both	Total cancers	39.06(26.85 to 54.43)	43.15(29.06 to 60.92)	0.52(0.28 to 0.75)	<0.001
Prevalence	Age-standardized	South Africa	Both	Total cancers	27.74(24.07 to 31.89)	34.08(28.92 to 39.9)	0.51(-0.09 to 1.12)	0.097
Prevalence	Age-standardized	South Sudan	Both	Total cancers	35.96(23.97 to 51.95)	59.1(38.67 to 83.4)	1.32(0.79 to 1.85)	<0.001
Prevalence	Age-standardized	Spain	Both	Total cancers	151.48(136.03 to 169.84)	157.8(137.67 to 181.3)	-0.05(-0.29 to 0.19)	0.682
Prevalence	Age-standardized	Sri Lanka	Both	Total cancers	53.37(44.42 to 63.54)	67.46(48.3 to 91.8)	0.29(-0.25 to 0.84)	0.293
Prevalence	Age-standardized	Sudan	Both	Total cancers	31.22(20.88 to 43.51)	53.65(35.89 to 78.08)	1.83(1.71 to 1.96)	<0.001
Prevalence	Age-standardized	Suriname	Both	Total cancers	49.3(30.92 to 60.93)	65.48(51.76 to 83.31)	0.62(-0.05 to 1.3)	0.07
Prevalence	Age-standardized	Sweden	Both	Total cancers	137.79(120.57 to 156.81)	155.52(134.28 to 177.85)	0.88(0.47 to 1.29)	<0.001
Prevalence	Age-standardized	Switzerland	Both	Total cancers	201.31(175.93 to 231.46)	157.79(133.7 to 189.51)	-0.73(-1.04 to	<0.001

								-0.42)	
Prevalence	Age-standardized	Syrian Arab Republic	Both	Total cancers	37.47(29.56 to 46.83)	68.7(50.84 to 90.42)	1.94(1.7 to 2.18)	<0.001	
Prevalence	Age-standardized	Taiwan (Province of China)	Both	Total cancers	88.65(80.9 to 97.34)	122.65(107.46 to 139.07)	0.83(0.22 to 1.45)	0.008	
Prevalence	Age-standardized	Tajikistan	Both	Total cancers	41.11(33.27 to 49.17)	40.64(31.66 to 51.2)	-0.48(-1.19 to 0.24)	0.191	
Prevalence	Age-standardized	Thailand	Both	Total cancers	54.36(44.08 to 65.91)	103.89(80.32 to 136)	2.1(1.79 to 2.4)	<0.001	
Prevalence	Age-standardized	Timor-Leste	Both	Total cancers	24.39(16.81 to 33.24)	37.6(26.65 to 51.56)	1.7(0.71 to 2.69)	0.001	
Prevalence	Age-standardized	Togo	Both	Total cancers	23.41(16.25 to 33.11)	29.35(18.93 to 43.78)	0.77(0.63 to 0.91)	<0.001	
Prevalence	Age-standardized	Tokelau	Both	Total cancers	30.79(21.01 to 42.74)	165.99(113.82 to 239.61)	5.94(4.38 to 7.52)	<0.001	
Prevalence	Age-standardized	Tonga	Both	Total cancers	40.01(27.13 to 59.53)	71.57(44.38 to 117.9)	1.83(1.51 to 2.15)	<0.001	
Prevalence	Age-standardized	Trinidad and Tobago	Both	Total cancers	55.89(50.45 to 61.68)	78.86(61.82 to 99.63)	1.61(1.31 to 1.91)	<0.001	
Prevalence	Age-standardized	Tunisia	Both	Total cancers	51.08(41.17 to 63.1)	92.93(67.34 to 128.55)	2.03(1.78 to 2.29)	<0.001	
Prevalence	Age-standardized	Turkey	Both	Total cancers	83.41(64.95 to 106.38)	176.15(130.89 to 237.87)	2.65(2.19 to 3.12)	<0.001	
Prevalence	Age-standardized	Turkmenistan	Both	Total cancers	45.76(41.34 to 50.58)	81.3(63.43 to 104.25)	1.91(1.55 to 2.26)	<0.001	
Prevalence	Age-standardized	Tuvalu	Both	Total cancers	25.61(19.26 to 33.43)	38.18(27.1 to 52.31)	1.29(1.23 to 1.36)	<0.001	
Prevalence	Age-standardized	Uganda	Both	Total cancers	48.03(32.38 to 68.9)	83.67(55.61 to 115.62)	1.47(1.06 to 1.87)	<0.001	
Prevalence	Age-standardized	Ukraine	Both	Total cancers	80.75(70.27 to 92.25)	71.1(53.31 to 91.25)	-0.63(-0.81 to -0.45)	<0.001	
Prevalence	Age-standardized	United Arab Emirates	Both	Total cancers	72.27(54.32 to 94.73)	101.48(77.32 to 131.88)	1(-0.49 to 2.5)	0.189	
Prevalence	Age-standardized	United Kingdom	Both	Total cancers	164.04(157.68 to 170.71)	153.01(146.29 to 160.39)	-0.15(-0.48 to 0.18)	0.361	
Prevalence	Age-standardized	United Republic of Tanzania	Both	Total cancers	54.06(39.68 to 72.03)	64.6(43.1 to 87.94)	0.48(0.37 to 0.6)	<0.001	

Prevalence	Age-standardized	United States Virgin Islands	Both	Total cancers	62.71(50.45 to 77.28)	99.45(72.56 to 131.16)	2.31(1.57 to 3.06)	<0.001
Prevalence	Age-standardized	United States of America	Both	Total cancers	156.55(152.34 to 160.96)	137.51(130.92 to 144.61)	-0.48(-0.6 to -0.37)	<0.001
Prevalence	Age-standardized	Uruguay	Both	Total cancers	74.87(64.54 to 87.27)	148.2(124.99 to 176.85)	2.05(1.85 to 2.25)	<0.001
Prevalence	Age-standardized	Uzbekistan	Both	Total cancers	41.94(36 to 49.64)	51.61(42.41 to 62.89)	0.87(0.52 to 1.22)	<0.001
Prevalence	Age-standardized	Vanuatu	Both	Total cancers	19(12.37 to 28.19)	28.74(19.64 to 40.12)	1.36(0.77 to 1.95)	<0.001
Prevalence	Age-standardized	Venezuela (Bolivarian Republic of)	Both	Total cancers	52.82(48.56 to 57.28)	101.9(76.61 to 130.65)	2.71(2.37 to 3.05)	<0.001
Prevalence	Age-standardized	Viet Nam	Both	Total cancers	34.26(24.96 to 46.27)	67.77(50.46 to 89.86)	2.32(2.21 to 2.43)	<0.001
Prevalence	Age-standardized	Yemen	Both	Total cancers	15.75(8.16 to 24.92)	26.22(14.54 to 41.71)	1.78(1.44 to 2.12)	<0.001
Prevalence	Age-standardized	Zambia	Both	Total cancers	56.53(43.4 to 72.01)	82.69(47.41 to 138.36)	1.16(0.78 to 1.53)	<0.001
Prevalence	Age-standardized	Zimbabwe	Both	Total cancers	32.93(23.8 to 44.13)	64.7(46.91 to 87.5)	2.73(1.72 to 3.75)	<0.001

Supplementary Table 3: AAPC Values of Cancer Disease Burden Among Adolescents Aged 10-24 in the World and 204 Countries/Regions (DALY)

Measure	Age	Location	Sex	Cause	1990 year Rate (95%UI)	2021 year Rate (95%UI)	AAPC(95%CI)	P Value
DALYs (Disability-Adjusted Life Years)	Age-standardized	Global	Both	Total cancers	495.45(445.54 to 535.36)	365.6(329.06 to 398.41)	-1.06(-1.11 to -1.01)	<0.001
DALYs (Disability-Adjusted Life Years)	Age-standardized	Afghanistan	Both	Total cancers	789.7(474.32 to 1126.37)	729.96(497.22 to 968.48)	-0.17(-0.28 to -0.07)	0.002
DALYs (Disability-Adjusted Life Years)	Age-standardized	Albania	Both	Total cancers	541.69(460.99 to 629.08)	295.47(231.59 to 366.38)	-1.25(-1.79 to -0.7)	<0.001
DALYs (Disability-Adjusted Life Years)	Age-standardized	Algeria	Both	Total cancers	423.31(333.04 to 528.37)	270.92(211.54 to 338.54)	-1.31(-1.4 to -1.22)	<0.001
DALYs (Disability-Adjusted Life Years)	Age-standardized	American Samoa	Both	Total cancers	327.75(259.94 to 410.77)	396.55(302.65 to 501.53)	0.75(0.6 to 0.9)	<0.001
DALYs (Disability-Adjusted Life Years)	Age-standardized	Andorra	Both	Total cancers	408.28(297.83 to 552.47)	267.97(195.38 to 356.44)	-1.28(-1.41 to -1.15)	<0.001
DALYs (Disability-Adjusted Life Years)	Age-standardized	Angola	Both	Total cancers	291.7(209.98 to 390.54)	262.38(175.39 to 371.16)	-0.21(-0.6 to 0.18)	0.285
DALYs (Disability-Adjusted Life Years)	Age-standardized	Antigua and Barbuda	Both	Total cancers	346.73(311.8 to 385.72)	328.6(299.55 to 360.98)	-0.01(-0.86 to 0.84)	0.979
DALYs (Disability-Adjusted Life Years)	Age-standardized	Argentina	Both	Total cancers	537(490.91 to 580.81)	399.38(367.39 to 434.53)	-1(-1.27 to -0.72)	<0.001
DALYs (Disability-Adjusted Life Years)	Age-standardized	Armenia	Both	Total cancers	527.99(490.54 to 570.76)	365.36(312.11 to 422.46)	-0.86(-1.74 to 0.02)	0.055
DALYs (Disability-Adjusted Life Years)	Age-standardized	Australia	Both	Total cancers	387.2(362.33 to 412.52)	213.7(194.32 to 235.08)	-2.17(-2.36 to -1.99)	<0.001
DALYs (Disability-Adjusted Life Years)	Age-standardized	Austria	Both	Total cancers	356.04(331.78 to 381.39)	220.46(201.61 to 241.78)	-1.35(-1.88 to -0.82)	<0.001
DALYs (Disability-Adjusted Life Years)	Age-standardized	Azerbaijan	Both	Total cancers	646(543.63 to 779.45)	498.88(382.09 to 638.42)	-1.2(-1.56 to -0.84)	<0.001
DALYs (Disability-Adjusted Life Years)	Age-standardized	Bahamas	Both	Total cancers	428.06(386.4 to 472.4)	435.52(346.48 to 538.87)	0.19(-0.34 to 0.72)	0.477
DALYs (Disability-Adjusted Life Years)	Age-standardized	Bahrain	Both	Total cancers	432.89(364.5 to 509.29)	262.18(213.44 to 325.28)	-1.17(-1.46 to -0.87)	<0.001
DALYs (Disability-Adjusted Life Years)	Age-standardized	Bangladesh	Both	Total cancers	499.27(382.03 to 632.13)	431.1(314.08 to 578.26)	-0.33(-0.56 to -0.1)	0.006
DALYs (Disability-Adjusted Life Years)	Age-standardized	Barbados	Both	Total cancers	625.64(574.29 to 680.17)	455.35(348.91 to 589.61)	-0.57(-0.82 to -0.32)	<0.001
DALYs (Disability-Adjusted Life Years)	Age-standardized	Belarus	Both	Total cancers	406.16(372.83 to 440.98)	269.7(226.18 to 318.67)	-1.67(-2.26 to -1.07)	<0.001
DALYs (Disability-Adjusted Life Years)	Age-standardized	Belgium	Both	Total cancers	417.3(386.77 to 449.36)	249.36(226.57 to 275.09)	-2.07(-2.59 to -1.55)	<0.001
DALYs (Disability-Adjusted Life Years)	Age-standardized	Belize	Both	Total cancers	325.14(285.6 to 373.57)	273.58(243.28 to 306.12)	0.32(-0.35 to 0.99)	0.349
DALYs (Disability-Adjusted Life Years)	Age-standardized	Benin	Both	Total cancers	238(176.38 to 324.8)	224.98(154.93 to 313.34)	-0.22(-0.33 to -0.1)	0.001
DALYs (Disability-Adjusted Life Years)	Age-standardized	Bermuda	Both	Total cancers	466.22(414.73 to 525.86)	317.04(264.97 to 389.53)	-1.23(-1.42 to -1.03)	<0.001
DALYs (Disability-Adjusted Life Years)	Age-standardized	Bhutan	Both	Total cancers	350.84(202.1 to 489.98)	327.19(214.11 to 471.65)	-0.12(-0.51 to 0.28)	0.568

DALYs (Disability-Adjusted Life Years)	Age-standardized	Bolivia (Plurinational State of)	Both	Total cancers	748.03(598.41 to 910.45)	578.94(409.63 to 805.04)	-0.82(-0.86 to -0.78)	<0.001
DALYs (Disability-Adjusted Life Years)	Age-standardized	Bosnia and Herzegovina	Both	Total cancers	394.83(339.39 to 458.58)	312.15(253.66 to 376.5)	-1.08(-1.63 to -0.53)	<0.001
DALYs (Disability-Adjusted Life Years)	Age-standardized	Botswana	Both	Total cancers	300.54(208.31 to 400.19)	274.4(192.54 to 366.75)	-0.27(-0.77 to 0.23)	0.284
DALYs (Disability-Adjusted Life Years)	Age-standardized	Brazil	Both	Total cancers	428.67(404.85 to 449.7)	406.02(381.55 to 427.45)	-0.13(-0.27 to 0.02)	0.081
DALYs (Disability-Adjusted Life Years)	Age-standardized	Brunei Darussalam	Both	Total cancers	685.14(546.23 to 835.05)	338.02(278.2 to 407.81)	-1.94(-2.1 to -1.78)	<0.001
DALYs (Disability-Adjusted Life Years)	Age-standardized	Bulgaria	Both	Total cancers	567.08(513.28 to 620.21)	412.16(350.03 to 466.62)	-0.96(-2.12 to 0.22)	0.112
DALYs (Disability-Adjusted Life Years)	Age-standardized	Burkina Faso	Both	Total cancers	243.8(174.04 to 354.75)	257.45(174.93 to 356.41)	0.44(0.17 to 0.7)	0.001
DALYs (Disability-Adjusted Life Years)	Age-standardized	Burundi	Both	Total cancers	632.38(485.16 to 804.62)	477.69(349.6 to 639.62)	-1.03(-1.21 to -0.85)	<0.001
DALYs (Disability-Adjusted Life Years)	Age-standardized	Cabo Verde	Both	Total cancers	272.73(205.27 to 352.01)	333.88(255.25 to 426.65)	0.53(0.26 to 0.81)	<0.001
DALYs (Disability-Adjusted Life Years)	Age-standardized	Cambodia	Both	Total cancers	590.95(437.19 to 772.52)	488.15(332.66 to 697.7)	-0.74(-0.99 to -0.5)	<0.001
DALYs (Disability-Adjusted Life Years)	Age-standardized	Cameroon	Both	Total cancers	265.73(203.84 to 345.17)	275.39(180.42 to 386.04)	0.03(-0.12 to 0.19)	0.662
DALYs (Disability-Adjusted Life Years)	Age-standardized	Canada	Both	Total cancers	346.66(326.24 to 368.75)	238.04(216.5 to 263.04)	-1.22(-1.33 to -1.12)	<0.001
DALYs (Disability-Adjusted Life Years)	Age-standardized	Central African Republic	Both	Total cancers	317.93(248.06 to 397.14)	295.4(216.15 to 385.6)	-0.3(-0.5 to -0.11)	0.003
DALYs (Disability-Adjusted Life Years)	Age-standardized	Chad	Both	Total cancers	197.92(140.69 to 277.97)	251.68(177.93 to 352.24)	1.01(0.82 to 1.19)	<0.001
DALYs (Disability-Adjusted Life Years)	Age-standardized	Chile	Both	Total cancers	439.72(406.37 to 475.28)	322.74(295.69 to 350.52)	-0.97(-1.33 to -0.61)	<0.001
DALYs (Disability-Adjusted Life Years)	Age-standardized	China	Both	Total cancers	657.43(556.84 to 747.65)	386.44(317.68 to 461.21)	-1.87(-2.23 to -1.5)	<0.001
DALYs (Disability-Adjusted Life Years)	Age-standardized	Colombia	Both	Total cancers	602.81(560.75 to 645.02)	494.14(419.78 to 572.83)	-0.72(-1.01 to -0.42)	<0.001
DALYs (Disability-Adjusted Life Years)	Age-standardized	Comoros	Both	Total cancers	534.65(211.86 to 752.21)	523.78(373.61 to 698.73)	-0.47(-1.91 to 0.99)	0.524
DALYs (Disability-Adjusted Life Years)	Age-standardized	Congo	Both	Total cancers	273.18(199.79 to 354.95)	276.24(208.23 to 352.65)	0.32(-0.26 to 0.9)	0.281
DALYs (Disability-Adjusted Life Years)	Age-standardized	Cook Islands	Both	Total cancers	190.75(146.31 to 249.01)	144.29(109.19 to 193.71)	-0.84(-0.91 to -0.76)	<0.001
DALYs (Disability-Adjusted Life Years)	Age-standardized	Costa Rica	Both	Total cancers	456.85(423.55 to 495.08)	524.44(472.01 to 577.99)	0.39(0.07 to 0.7)	0.016
DALYs (Disability-Adjusted Life Years)	Age-standardized	Coted'Ivoire	Both	Total cancers	208.85(153.16 to 281.19)	217.43(147.1 to 303.63)	0.08(-0.28 to 0.45)	0.65
DALYs (Disability-Adjusted Life Years)	Age-standardized	Croatia	Both	Total cancers	393.97(356.03 to 436.75)	266.12(231.68 to 299.55)	-0.89(-2.13 to 0.37)	0.166

DALYs (Disability-Adjusted Life Years)	Age-standardized	Cuba	Both	Total cancers	553.69(513.96 to 597.9)	369.05(319.82 to 421.71)	-1.13(-1.44 to -0.82)	<0.001
DALYs (Disability-Adjusted Life Years)	Age-standardized	Cyprus	Both	Total cancers	273.33(222.67 to 334.51)	173.45(138.07 to 214.9)	-0.98(-1.58 to -0.36)	0.002
DALYs (Disability-Adjusted Life Years)	Age-standardized	Czechia	Both	Total cancers	440.6(406.56 to 476.54)	260.49(223.83 to 301.69)	-1.11(-1.5 to -0.72)	<0.001
DALYs (Disability-Adjusted Life Years)	Age-standardized	Democratic People's Republic of Korea	Both	Total cancers	544.56(370.23 to 762.46)	452.44(340.06 to 618.32)	-0.67(-0.76 to -0.59)	<0.001
DALYs (Disability-Adjusted Life Years)	Age-standardized	Democratic Republic of the Congo	Both	Total cancers	240.78(180.7 to 310.28)	223(163.41 to 298.37)	-0.29(-0.5 to -0.08)	0.007
DALYs (Disability-Adjusted Life Years)	Age-standardized	Denmark	Both	Total cancers	317.05(291.31 to 343.74)	187.21(168.84 to 206.98)	-2.03(-2.67 to -1.39)	<0.001
DALYs (Disability-Adjusted Life Years)	Age-standardized	Djibouti	Both	Total cancers	364.95(253.4 to 502.61)	398.65(254.64 to 592.68)	0.54(0.04 to 1.03)	0.033
DALYs (Disability-Adjusted Life Years)	Age-standardized	Dominica	Both	Total cancers	467.06(394.25 to 556.7)	564.71(433.91 to 705.12)	0.82(0.64 to 1)	<0.001
DALYs (Disability-Adjusted Life Years)	Age-standardized	Dominican Republic	Both	Total cancers	381.26(320.05 to 452.92)	385.69(302.81 to 482.07)	0.41(0.14 to 0.68)	0.003
DALYs (Disability-Adjusted Life Years)	Age-standardized	Ecuador	Both	Total cancers	533.46(481.21 to 584.1)	537.85(446.94 to 638.07)	0.62(0.17 to 1.06)	0.006
DALYs (Disability-Adjusted Life Years)	Age-standardized	Egypt	Both	Total cancers	428.96(355.4 to 531.55)	427.2(339.21 to 531.51)	0.33(0.08 to 0.58)	0.011
DALYs (Disability-Adjusted Life Years)	Age-standardized	El Salvador	Both	Total cancers	518.3(450.53 to 609.28)	499.36(377.43 to 638.06)	0.08(-0.31 to 0.47)	0.697
DALYs (Disability-Adjusted Life Years)	Age-standardized	Equatorial Guinea	Both	Total cancers	281.97(205.56 to 373.79)	244.97(136.11 to 447.52)	-0.44(-0.83 to -0.05)	0.029
DALYs (Disability-Adjusted Life Years)	Age-standardized	Eritrea	Both	Total cancers	491.3(380.79 to 627.2)	498.09(359.72 to 659.52)	0.01(-0.09 to 0.11)	0.81
DALYs (Disability-Adjusted Life Years)	Age-standardized	Estonia	Both	Total cancers	492.82(449.74 to 540.99)	332.47(289.13 to 378.29)	-1.7(-2.46 to -0.94)	<0.001
DALYs (Disability-Adjusted Life Years)	Age-standardized	Eswatini	Both	Total cancers	322.01(249.67 to 404.57)	438.7(264.51 to 626.38)	1.32(0.91 to 1.73)	<0.001
DALYs (Disability-Adjusted Life Years)	Age-standardized	Ethiopia	Both	Total cancers	928.33(687.18 to 1178.39)	561.91(434.23 to 700.22)	-1.86(-2.08 to -1.64)	<0.001
DALYs (Disability-Adjusted Life Years)	Age-standardized	Fiji	Both	Total cancers	480.74(369.77 to 602.51)	452.63(324.63 to 609.11)	-0.03(-0.22 to 0.16)	0.745
DALYs (Disability-Adjusted Life Years)	Age-standardized	Finland	Both	Total cancers	315.3(291.75 to 340.51)	247.89(222.44 to 277.08)	-0.71(-0.95 to -0.48)	<0.001
DALYs (Disability-Adjusted Life Years)	Age-standardized	France	Both	Total cancers	393.86(371.65 to 417.12)	233.97(215.68 to 256.44)	-1.73(-1.83 to -1.64)	<0.001
DALYs (Disability-Adjusted Life Years)	Age-standardized	Gabon	Both	Total cancers	257.36(199.5 to 321.8)	250.04(162.9 to 355.83)	-0.12(-0.34 to 0.11)	0.304
DALYs (Disability-Adjusted Life Years)	Age-standardized	Gambia	Both	Total cancers	245.14(169.24 to 335.78)	295.06(192.7 to 411.22)	0.25(-0.06 to 0.56)	0.111
DALYs (Disability-Adjusted Life Years)	Age-standardized	Georgia	Both	Total cancers	598.84(523.18 to 669.23)	514.36(439.75 to 585.34)	-0.55(-1.38 to 0.28)	0.193
DALYs (Disability-Adjusted Life Years)	Age-standardized	Germany	Both	Total cancers	394.67(370.55 to 419.5)	222.89(205.26 to 243.62)	-1.95(-2.15 to -1.75)	<0.001

DALYs (Disability-Adjusted Life Years)	Age-standardized	Ghana	Both	Total cancers	406.31(252.32 to 566.17)	268.29(191.38 to 364.09)	-1.71(-2.31 to -1.11)	<0.001
DALYs (Disability-Adjusted Life Years)	Age-standardized	Greece	Both	Total cancers	467.92(442.27 to 496.75)	315.97(291.78 to 341.4)	-1.27(-1.6 to -0.94)	<0.001
DALYs (Disability-Adjusted Life Years)	Age-standardized	Greenland	Both	Total cancers	612.55(457.68 to 908.01)	197(142.44 to 271.21)	-3.72(-3.94 to -3.5)	<0.001
DALYs (Disability-Adjusted Life Years)	Age-standardized	Grenada	Both	Total cancers	613.31(537.56 to 699.85)	462.16(394.56 to 539.63)	-0.48(-0.86 to -0.1)	0.014
DALYs (Disability-Adjusted Life Years)	Age-standardized	Guam	Both	Total cancers	332.13(277.3 to 389.54)	236.53(187.97 to 289.54)	-1.22(-3.86 to 1.49)	0.373
DALYs (Disability-Adjusted Life Years)	Age-standardized	Guatemala	Both	Total cancers	472.23(442.23 to 505.53)	442.81(382.01 to 509.95)	-0.09(-0.47 to 0.3)	0.664
DALYs (Disability-Adjusted Life Years)	Age-standardized	Guinea	Both	Total cancers	293.44(215.76 to 391.43)	296.48(209.92 to 419)	0.06(-0.12 to 0.25)	0.514
DALYs (Disability-Adjusted Life Years)	Age-standardized	Guinea-Bissau	Both	Total cancers	382.93(275.12 to 506.92)	344.47(249.55 to 497.08)	-0.29(-0.37 to -0.2)	<0.001
DALYs (Disability-Adjusted Life Years)	Age-standardized	Guyana	Both	Total cancers	371.83(288.29 to 439.59)	379.46(286.73 to 489.95)	0.73(0.2 to 1.27)	0.007
DALYs (Disability-Adjusted Life Years)	Age-standardized	Haiti	Both	Total cancers	735.48(492.72 to 984.34)	611.48(395.33 to 859.34)	-0.39(-0.73 to -0.05)	0.026
DALYs (Disability-Adjusted Life Years)	Age-standardized	Honduras	Both	Total cancers	515.74(422.43 to 628.05)	310.73(193.94 to 470.54)	-1.79(-1.99 to -1.58)	<0.001
DALYs (Disability-Adjusted Life Years)	Age-standardized	Hungary	Both	Total cancers	458.2(427.35 to 492.76)	267.13(234.61 to 302.1)	-1.5(-1.83 to -1.17)	<0.001
DALYs (Disability-Adjusted Life Years)	Age-standardized	Iceland	Both	Total cancers	402.44(364.77 to 440.94)	299.94(265.21 to 338.39)	-1.24(-1.72 to -0.75)	<0.001
DALYs (Disability-Adjusted Life Years)	Age-standardized	India	Both	Total cancers	378.39(316.85 to 437.85)	297.92(254.41 to 350.53)	-0.76(-0.91 to -0.6)	<0.001
DALYs (Disability-Adjusted Life Years)	Age-standardized	Indonesia	Both	Total cancers	460.96(370.9 to 553.88)	425.65(338.33 to 541.99)	-0.26(-0.31 to -0.2)	<0.001
DALYs (Disability-Adjusted Life Years)	Age-standardized	Iran (Islamic Republic of)	Both	Total cancers	556.58(425.96 to 653.18)	459.87(361.26 to 533.43)	-0.43(-0.76 to -0.1)	0.012
DALYs (Disability-Adjusted Life Years)	Age-standardized	Iraq	Both	Total cancers	535.31(412.07 to 694.76)	393.31(296.19 to 518.04)	-1.13(-1.29 to -0.96)	<0.001
DALYs (Disability-Adjusted Life Years)	Age-standardized	Ireland	Both	Total cancers	373.49(344.04 to 405.6)	171.44(153.49 to 191.51)	-2.4(-2.81 to -1.99)	<0.001
DALYs (Disability-Adjusted Life Years)	Age-standardized	Israel	Both	Total cancers	372.24(342.56 to 404.66)	225.71(204.73 to 250.35)	-1.51(-1.92 to -1.11)	<0.001
DALYs (Disability-Adjusted Life Years)	Age-standardized	Italy	Both	Total cancers	491.78(477.83 to 505.34)	269.04(256.48 to 282.81)	-2.22(-2.69 to -1.74)	<0.001
DALYs (Disability-Adjusted Life Years)	Age-standardized	Jamaica	Both	Total cancers	340.74(297.25 to 381.05)	297.04(232.75 to 380.24)	-1.08(-1.7 to -0.46)	0.001
DALYs (Disability-Adjusted Life Years)	Age-standardized	Japan	Both	Total cancers	329.99(322.47 to 338.11)	214.54(208.74 to 220.63)	-1.34(-1.59 to -1.1)	<0.001
DALYs (Disability-Adjusted Life Years)	Age-standardized	Jordan	Both	Total cancers	480.3(390.17 to 596.53)	305.4(237.23 to 386.48)	-1.56(-1.73 to -1.39)	<0.001
DALYs (Disability-Adjusted Life Years)	Age-standardized	Kazakhstan	Both	Total cancers	699.22(648.69 to 753.63)	292.56(256.93 to 333.33)	-2.79(-3.07 to -2.52)	<0.001
DALYs (Disability-Adjusted Life Years)	Age-standardized	Kenya	Both	Total cancers	292.49(225.25 to 360.44)	332.15(256.91 to 426.54)	0.71(0.31 to 1.12)	0.001

DALYs (Disability-Adjusted Life Years)	Age-standardized	Kiribati	Both	Total cancers	392.54(298.15 to 500.15)	404.52(278.69 to 568.71)	0.11(0.07 to 0.14)	<0.001
DALYs (Disability-Adjusted Life Years)	Age-standardized	Kuwait	Both	Total cancers	369.02(331.05 to 410.42)	190.65(158.5 to 229.47)	-2.37(-2.7 to -2.04)	<0.001
DALYs (Disability-Adjusted Life Years)	Age-standardized	Kyrgyzstan	Both	Total cancers	484.71(421.63 to 552.55)	330.64(278.15 to 393.83)	-0.86(-1.24 to -0.49)	<0.001
DALYs (Disability-Adjusted Life Years)	Age-standardized	Lao People's Democratic Republic	Both	Total cancers	590.54(396.01 to 814.03)	486.37(334.34 to 684.19)	-0.63(-0.71 to -0.54)	<0.001
DALYs (Disability-Adjusted Life Years)	Age-standardized	Latvia	Both	Total cancers	459.53(422.71 to 498.57)	276.61(245.03 to 306.72)	-1.05(-1.68 to -0.42)	0.001
DALYs (Disability-Adjusted Life Years)	Age-standardized	Lebanon	Both	Total cancers	449.88(339.06 to 596.71)	291.65(229.96 to 372.35)	-1.26(-1.43 to -1.08)	<0.001
DALYs (Disability-Adjusted Life Years)	Age-standardized	Lesotho	Both	Total cancers	228.45(168.73 to 295.34)	415.4(291.17 to 553.7)	2.61(2.02 to 3.2)	<0.001
DALYs (Disability-Adjusted Life Years)	Age-standardized	Liberia	Both	Total cancers	253.12(180.9 to 342.04)	290.73(195.92 to 401.57)	0.62(0.17 to 1.07)	0.007
DALYs (Disability-Adjusted Life Years)	Age-standardized	Libya	Both	Total cancers	496.16(394.37 to 617.28)	502.08(375.71 to 657.14)	0.44(-0.04 to 0.91)	0.07
DALYs (Disability-Adjusted Life Years)	Age-standardized	Lithuania	Both	Total cancers	387.44(355.46 to 422.54)	279.52(247.83 to 311.13)	-0.85(-1.14 to -0.55)	<0.001
DALYs (Disability-Adjusted Life Years)	Age-standardized	Luxembourg	Both	Total cancers	511.03(469.93 to 556.1)	191.52(168.76 to 217.61)	-3.99(-4.25 to -3.73)	<0.001
DALYs (Disability-Adjusted Life Years)	Age-standardized	Madagascar	Both	Total cancers	477.58(382.08 to 593.41)	458.59(325.05 to 602.14)	-0.1(-0.19 to -0.02)	0.021
DALYs (Disability-Adjusted Life Years)	Age-standardized	Malawi	Both	Total cancers	536.29(401.45 to 697.66)	542(364.65 to 780.48)	0.12(0.04 to 0.21)	0.005
DALYs (Disability-Adjusted Life Years)	Age-standardized	Malaysia	Both	Total cancers	466.16(361.3 to 578.7)	343.58(270.44 to 421.76)	-1.21(-1.63 to -0.77)	<0.001
DALYs (Disability-Adjusted Life Years)	Age-standardized	Maldives	Both	Total cancers	351.33(246.47 to 463.06)	187.8(147.18 to 237.8)	-1.8(-2.06 to -1.55)	<0.001
DALYs (Disability-Adjusted Life Years)	Age-standardized	Mali	Both	Total cancers	303(226.9 to 389.7)	262.69(190.88 to 345.03)	-0.34(-0.46 to -0.22)	<0.001
DALYs (Disability-Adjusted Life Years)	Age-standardized	Malta	Both	Total cancers	354.17(310.95 to 391.82)	371.53(311.57 to 435.39)	-0.79(-1.29 to -0.29)	0.003
DALYs (Disability-Adjusted Life Years)	Age-standardized	Marshall Islands	Both	Total cancers	362.26(281.7 to 468.69)	394.04(278.69 to 532.79)	0.38(0.11 to 0.65)	0.005
DALYs (Disability-Adjusted Life Years)	Age-standardized	Mauritania	Both	Total cancers	275.38(191.82 to 387.8)	218.53(156.85 to 301.94)	-0.75(-0.96 to -0.54)	<0.001
DALYs (Disability-Adjusted Life Years)	Age-standardized	Mauritius	Both	Total cancers	300(278.22 to 322.01)	277.26(246.18 to 300.79)	-0.25(-0.74 to 0.24)	0.324
DALYs (Disability-Adjusted Life Years)	Age-standardized	Mexico	Both	Total cancers	510.91(495.25 to 528.82)	521.44(481.19 to 562.59)	0.26(0.14 to 0.37)	<0.001
DALYs (Disability-Adjusted Life Years)	Age-standardized	Micronesia (Federated States of)	Both	Total cancers	394.26(305.2 to 504.46)	380.67(266.15 to 524.14)	-0.15(-0.2 to -0.09)	<0.001
DALYs (Disability-Adjusted Life Years)	Age-standardized	Monaco	Both	Total cancers	515.15(390.3 to 654.93)	512.87(400.29 to 664.88)	-0.12(-0.24 to -0.01)	0.038
DALYs (Disability-Adjusted Life Years)	Age-standardized	Mongolia	Both	Total cancers	573.39(475.29 to 706.57)	489.34(417.78 to 570.88)	-0.6(-1.03 to -0.17)	0.008

DALYs (Disability-Adjusted Life Years)	Age-standardized	Montenegro	Both	Total cancers	446.81(375.34 to 525.79)	356.32(287.62 to 433.51)	-0.42(-0.99 to 0.14)	0.143
DALYs (Disability-Adjusted Life Years)	Age-standardized	Morocco	Both	Total cancers	234.97(181.89 to 297.22)	182.42(137.55 to 260.92)	-0.85(-0.92 to -0.78)	<0.001
DALYs (Disability-Adjusted Life Years)	Age-standardized	Mozambique	Both	Total cancers	376.91(278.76 to 508.31)	429.23(279.67 to 610.77)	0.56(0.27 to 0.86)	<0.001
DALYs (Disability-Adjusted Life Years)	Age-standardized	Myanmar	Both	Total cancers	681.86(447.03 to 937.86)	470.83(347.47 to 644.04)	-1.38(-1.73 to -1.03)	<0.001
DALYs (Disability-Adjusted Life Years)	Age-standardized	Namibia	Both	Total cancers	313.21(238.43 to 401.96)	327.11(228.84 to 493.14)	0.21(0.07 to 0.34)	0.003
DALYs (Disability-Adjusted Life Years)	Age-standardized	Nauru	Both	Total cancers	446.64(337.96 to 573.18)	476.45(333.57 to 643.32)	0.22(0.17 to 0.27)	<0.001
DALYs (Disability-Adjusted Life Years)	Age-standardized	Nepal	Both	Total cancers	400.53(289.15 to 522.63)	338.83(242.08 to 472.56)	-0.46(-0.59 to -0.32)	<0.001
DALYs (Disability-Adjusted Life Years)	Age-standardized	Netherlands	Both	Total cancers	327.11(306.38 to 347.08)	196.84(179.11 to 215.22)	-1.8(-2.13 to -1.47)	<0.001
DALYs (Disability-Adjusted Life Years)	Age-standardized	New Zealand	Both	Total cancers	436.6(403.49 to 473.06)	245.32(223.6 to 267.71)	-1.82(-2.14 to -1.49)	<0.001
DALYs (Disability-Adjusted Life Years)	Age-standardized	Nicaragua	Both	Total cancers	412.67(354.63 to 491.19)	314.66(251.21 to 386.63)	-1.17(-1.7 to -0.65)	<0.001
DALYs (Disability-Adjusted Life Years)	Age-standardized	Niger	Both	Total cancers	236.18(165.13 to 336.11)	197.4(127.43 to 293.5)	-0.5(-0.68 to -0.33)	<0.001
DALYs (Disability-Adjusted Life Years)	Age-standardized	Nigeria	Both	Total cancers	295.94(186.76 to 414.81)	258.71(148.43 to 397.07)	-0.46(-0.66 to -0.25)	<0.001
DALYs (Disability-Adjusted Life Years)	Age-standardized	Niue	Both	Total cancers	305.09(219.59 to 426.41)	762.78(599.44 to 963.58)	3.05(1.91 to 4.21)	<0.001
DALYs (Disability-Adjusted Life Years)	Age-standardized	North Macedonia	Both	Total cancers	536.21(469.06 to 618.66)	406.58(318.17 to 503.4)	-0.96(-1.35 to -0.57)	<0.001
DALYs (Disability-Adjusted Life Years)	Age-standardized	Northern Mariana Islands	Both	Total cancers	251.85(186.01 to 338.22)	234.45(185.88 to 289.16)	-0.25(-0.79 to 0.29)	0.355
DALYs (Disability-Adjusted Life Years)	Age-standardized	Norway	Both	Total cancers	325.43(312.02 to 338.78)	262.78(249.15 to 277.57)	-1.26(-1.92 to -0.6)	<0.001
DALYs (Disability-Adjusted Life Years)	Age-standardized	Oman	Both	Total cancers	248.29(179.04 to 340.89)	160.03(126.75 to 200.84)	-1.04(-2.06 to -0.01)	0.048
DALYs (Disability-Adjusted Life Years)	Age-standardized	Pakistan	Both	Total cancers	528.57(421.81 to 634.81)	677.38(546.7 to 838.89)	0.79(0.63 to 0.95)	<0.001
DALYs (Disability-Adjusted Life Years)	Age-standardized	Palau	Both	Total cancers	426.33(289.13 to 610)	508.46(381.43 to 675.52)	0.52(0.39 to 0.65)	<0.001
DALYs (Disability-Adjusted Life Years)	Age-standardized	Palestine	Both	Total cancers	530.75(398.02 to 704.99)	388.84(316.21 to 487.39)	-0.82(-1.16 to -0.48)	<0.001
DALYs (Disability-Adjusted Life Years)	Age-standardized	Panama	Both	Total cancers	515.23(473.79 to 562.41)	477.13(395.75 to 561.95)	-0.21(-0.85 to 0.42)	0.509
DALYs (Disability-Adjusted Life Years)	Age-standardized	Papua New Guinea	Both	Total cancers	297.02(113.66 to 453.08)	321.6(187.49 to 465.88)	0.18(0.04 to 0.32)	0.014
DALYs (Disability-Adjusted Life Years)	Age-standardized	Paraguay	Both	Total cancers	366.32(306.55 to 442.03)	397.11(310.5 to 512.75)	0.3(0.07 to 0.53)	0.009
DALYs (Disability-Adjusted Life Years)	Age-standardized	Peru	Both	Total cancers	639.78(542.35 to 753.83)	482.04(372.88 to 619.4)	-0.69(-0.82 to -0.56)	<0.001
DALYs (Disability-Adjusted Life Years)	Age-standardized	Philippines	Both	Total cancers	504.82(441.98 to 564.43)	434.22(369.53 to 500.17)	-0.34(-0.58 to -0.1)	0.005

DALYs (Disability-Adjusted Life Years)	Age-standardized	Poland	Both	Total cancers	479.53(463.17 to 493.22)	267.18(249.43 to 281.84)	-1.67(-1.77 to -1.58)	<0.001
DALYs (Disability-Adjusted Life Years)	Age-standardized	Portugal	Both	Total cancers	592.98(552.4 to 637.91)	237.43(213.37 to 263.64)	-3.36(-3.63 to -3.1)	<0.001
DALYs (Disability-Adjusted Life Years)	Age-standardized	Puerto Rico	Both	Total cancers	427.14(388.48 to 468.65)	285.72(236.18 to 338.44)	-1.35(-1.83 to -0.88)	<0.001
DALYs (Disability-Adjusted Life Years)	Age-standardized	Qatar	Both	Total cancers	353.95(272.66 to 457.35)	212.51(160.44 to 281.63)	-1.23(-1.58 to -0.88)	<0.001
DALYs (Disability-Adjusted Life Years)	Age-standardized	Republic of Korea	Both	Total cancers	603.95(467.99 to 690.58)	207.33(161.81 to 243.3)	-3.82(-4.19 to -3.46)	<0.001
DALYs (Disability-Adjusted Life Years)	Age-standardized	Republic of Moldova	Both	Total cancers	567.01(531.04 to 606.49)	249.52(220.3 to 283.11)	-2.11(-2.63 to -1.59)	<0.001
DALYs (Disability-Adjusted Life Years)	Age-standardized	Romania	Both	Total cancers	525.75(484.68 to 568.69)	311.07(276.19 to 349.38)	-1.43(-1.76 to -1.11)	<0.001
DALYs (Disability-Adjusted Life Years)	Age-standardized	Russian Federation	Both	Total cancers	574.94(558.18 to 591.28)	272.05(256.24 to 286.95)	-2.62(-3.13 to -2.12)	<0.001
DALYs (Disability-Adjusted Life Years)	Age-standardized	Rwanda	Both	Total cancers	756.01(609.74 to 918.45)	458.65(321.96 to 636.05)	-1.98(-2.64 to -1.33)	<0.001
DALYs (Disability-Adjusted Life Years)	Age-standardized	Saint Kitts and Nevis	Both	Total cancers	415.3(376.89 to 456.11)	377.7(313.25 to 458.14)	-0.62(-1.05 to -0.19)	0.004
DALYs (Disability-Adjusted Life Years)	Age-standardized	Saint Lucia	Both	Total cancers	443.95(406.74 to 482.17)	425.14(350.46 to 512.63)	-0.28(-0.63 to 0.07)	0.122
DALYs (Disability-Adjusted Life Years)	Age-standardized	Saint Vincent and the Grenadines	Both	Total cancers	552.45(494.22 to 619.23)	623.8(538.96 to 723.54)	0.48(0.08 to 0.88)	0.017
DALYs (Disability-Adjusted Life Years)	Age-standardized	Samoa	Both	Total cancers	404.36(282.58 to 560.08)	387.1(274.75 to 536.03)	-0.07(-0.15 to 0.01)	0.08
DALYs (Disability-Adjusted Life Years)	Age-standardized	San Marino	Both	Total cancers	482.15(398.07 to 591.37)	316.55(231.86 to 423.38)	-1.41(-1.54 to -1.28)	<0.001
DALYs (Disability-Adjusted Life Years)	Age-standardized	Sao Tome and Principe	Both	Total cancers	184.8(98.49 to 301.26)	208.56(136.13 to 319.12)	0.61(-0.12 to 1.34)	0.101
DALYs (Disability-Adjusted Life Years)	Age-standardized	Saudi Arabia	Both	Total cancers	282.09(205.92 to 378.6)	241.61(185.1 to 313.37)	-0.39(-0.51 to -0.27)	<0.001
DALYs (Disability-Adjusted Life Years)	Age-standardized	Senegal	Both	Total cancers	251.61(187.22 to 338.23)	243.8(174.45 to 337.3)	0.16(-0.02 to 0.35)	0.084
DALYs (Disability-Adjusted Life Years)	Age-standardized	Serbia	Both	Total cancers	489.7(412.13 to 573.31)	216.31(177.51 to 263.75)	-2.55(-3 to -2.1)	<0.001
DALYs (Disability-Adjusted Life Years)	Age-standardized	Seychelles	Both	Total cancers	496.72(420.37 to 585.15)	204.92(170.41 to 242.75)	-0.51(-1.11 to 0.1)	0.099
DALYs (Disability-Adjusted Life Years)	Age-standardized	Sierra Leone	Both	Total cancers	201.71(131.82 to 302.96)	239.74(158.99 to 342.42)	0.8(0.45 to 1.15)	<0.001
DALYs (Disability-Adjusted Life Years)	Age-standardized	Singapore	Both	Total cancers	372.23(343.39 to 403.89)	322.76(292.12 to 356.42)	-0.94(-1.63 to -0.25)	0.008
DALYs (Disability-Adjusted Life Years)	Age-standardized	Slovakia	Both	Total cancers	393.92(345.18 to 454.02)	280.15(230.49 to 333.16)	-1.07(-1.75 to -0.38)	0.002
DALYs (Disability-Adjusted Life Years)	Age-standardized	Slovenia	Both	Total cancers	351.86(326.87 to 380.22)	198.61(171.61 to 233.27)	-1.95(-2.38 to -1.53)	<0.001
DALYs (Disability-Adjusted Life Years)	Age-standardized	Solomon Islands	Both	Total cancers	296.63(127.51 to 456.26)	353.84(235.27 to 494.07)	0.64(0.5 to 0.77)	<0.001
DALYs (Disability-Adjusted Life Years)	Age-standardized	Somalia	Both	Total cancers	455.33(324.32 to 597.11)	467.34(337.18 to 623.55)	0.29(0.05 to 0.54)	0.018

DALYs (Disability-Adjusted Life Years)	Age-standardized	South Africa	Both	Total cancers	255.22(221.78 to 292.49)	248.29(214.75 to 287.37)	-0.35(-0.85 to 0.16)	0.177
DALYs (Disability-Adjusted Life Years)	Age-standardized	South Sudan	Both	Total cancers	366.56(255.7 to 504.21)	508.78(353.54 to 703.47)	0.75(0.21 to 1.29)	0.006
DALYs (Disability-Adjusted Life Years)	Age-standardized	Spain	Both	Total cancers	483.01(453.09 to 512.51)	256.75(234.35 to 280.89)	-2.29(-2.51 to -2.07)	<0.001
DALYs (Disability-Adjusted Life Years)	Age-standardized	Sri Lanka	Both	Total cancers	512.27(408.72 to 619.08)	284.18(202.18 to 380.78)	-2.29(-2.85 to -1.72)	<0.001
DALYs (Disability-Adjusted Life Years)	Age-standardized	Sudan	Both	Total cancers	487.93(324.65 to 670.33)	464.72(296.79 to 675.67)	-0.04(-0.18 to 0.11)	0.607
DALYs (Disability-Adjusted Life Years)	Age-standardized	Suriname	Both	Total cancers	490.33(310.8 to 583.84)	493.1(403.51 to 599.95)	-0.3(-0.9 to 0.3)	0.322
DALYs (Disability-Adjusted Life Years)	Age-standardized	Sweden	Both	Total cancers	318.91(295.42 to 343.9)	216.4(194.84 to 239.07)	-0.84(-1.02 to -0.66)	<0.001
DALYs (Disability-Adjusted Life Years)	Age-standardized	Switzerland	Both	Total cancers	409.48(378.02 to 442.83)	194.91(175.77 to 217.16)	-2.38(-2.65 to -2.1)	<0.001
DALYs (Disability-Adjusted Life Years)	Age-standardized	Syrian Arab Republic	Both	Total cancers	521.02(404.29 to 651.85)	368.37(278.05 to 483.78)	-1.33(-1.74 to -0.92)	<0.001
DALYs (Disability-Adjusted Life Years)	Age-standardized	Taiwan (Province of China)	Both	Total cancers	422.74(397.99 to 448.95)	311.97(280.91 to 342)	-1.12(-1.58 to -0.65)	<0.001
DALYs (Disability-Adjusted Life Years)	Age-standardized	Tajikistan	Both	Total cancers	585.21(495.26 to 689.7)	475.98(371.72 to 602.39)	-1.12(-1.82 to -0.42)	0.002
DALYs (Disability-Adjusted Life Years)	Age-standardized	Thailand	Both	Total cancers	503.78(409.72 to 601.61)	449.51(346.78 to 581.38)	-0.48(-0.85 to -0.1)	0.014
DALYs (Disability-Adjusted Life Years)	Age-standardized	Timor-Leste	Both	Total cancers	393.06(273.73 to 538.77)	376.33(274.73 to 507.45)	-0.06(-0.85 to 0.73)	0.875
DALYs (Disability-Adjusted Life Years)	Age-standardized	Togo	Both	Total cancers	223.13(168.95 to 294.81)	233.08(156.87 to 328.41)	0.14(-0.03 to 0.3)	0.102
DALYs (Disability-Adjusted Life Years)	Age-standardized	Tokelau	Both	Total cancers	336.63(235.77 to 445.47)	845.62(630.41 to 1114.5)	3.38(1.97 to 4.81)	<0.001
DALYs (Disability-Adjusted Life Years)	Age-standardized	Tonga	Both	Total cancers	291.08(223.85 to 378.8)	354.26(258.35 to 504.19)	0.86(0.58 to 1.13)	<0.001
DALYs (Disability-Adjusted Life Years)	Age-standardized	Trinidad and Tobago	Both	Total cancers	458.16(420.01 to 494.35)	426.03(335.03 to 533.93)	0.26(-0.03 to 0.55)	0.079
DALYs (Disability-Adjusted Life Years)	Age-standardized	Tunisia	Both	Total cancers	348.39(287.17 to 422.27)	272.83(200.9 to 369.04)	-0.73(-0.89 to -0.57)	<0.001
DALYs (Disability-Adjusted Life Years)	Age-standardized	Turkey	Both	Total cancers	811.83(632.32 to 1013.02)	437.03(349.03 to 537.57)	-1.98(-2.23 to -1.74)	<0.001
DALYs (Disability-Adjusted Life Years)	Age-standardized	Turkmenistan	Both	Total cancers	540.13(507.01 to 577.82)	669.37(539.96 to 836.05)	0.72(0.36 to 1.08)	<0.001
DALYs (Disability-Adjusted Life Years)	Age-standardized	Tuvalu	Both	Total cancers	365.5(281.45 to 466.72)	318.55(235.03 to 411.72)	-0.4(-0.46 to -0.34)	<0.001
DALYs (Disability-Adjusted Life Years)	Age-standardized	Uganda	Both	Total cancers	449.66(311.52 to 624.25)	609.69(421.15 to 821.1)	0.71(0.33 to 1.1)	<0.001
DALYs (Disability-Adjusted Life Years)	Age-standardized	Ukraine	Both	Total cancers	679.8(617.95 to 745.36)	366.56(282.75 to 455.25)	-2.4(-3.06 to -1.73)	<0.001
DALYs (Disability-Adjusted Life Years)	Age-standardized	United Arab Emirates	Both	Total cancers	547.81(412.34 to 727.26)	371.62(286.32 to 465.39)	-1.23(-2.57 to 0.14)	0.078
DALYs (Disability-Adjusted Life Years)	Age-standardized	United Kingdom	Both	Total cancers	374.49(367.88 to 381.07)	244.36(236.2 to 252.24)	-1.39(-1.52 to -1.25)	<0.001

DALYs (Disability-Adjusted Life Years)	Age-standardized	United Republic of Tanzania	Both	Total cancers	504.63(393.88 to 637.13)	473.95(330.3 to 628.82)	-0.19(-0.3 to -0.08)	0.001
DALYs (Disability-Adjusted Life Years)	Age-standardized	United States Virgin Islands	Both	Total cancers	482.87(385.19 to 603.17)	459.78(322.02 to 616.19)	0.59(0.27 to 0.91)	0.001
DALYs (Disability-Adjusted Life Years)	Age-standardized	United States of America	Both	Total cancers	355.41(348.72 to 362.78)	233.58(222.73 to 244.81)	-1.44(-1.53 to -1.36)	<0.001
DALYs (Disability-Adjusted Life Years)	Age-standardized	Uruguay	Both	Total cancers	478.27(436.59 to 522.28)	468.88(421.42 to 520.17)	-0.27(-0.68 to 0.14)	0.194
DALYs (Disability-Adjusted Life Years)	Age-standardized	Uzbekistan	Both	Total cancers	515.21(461.36 to 575.87)	433.06(367.42 to 507.93)	-0.4(-0.76 to -0.04)	0.028
DALYs (Disability-Adjusted Life Years)	Age-standardized	Vanuatu	Both	Total cancers	263.7(170.75 to 377.1)	311.8(220.83 to 419.09)	0.59(0.22 to 0.96)	0.002
DALYs (Disability-Adjusted Life Years)	Age-standardized	Venezuela (Bolivarian Republic of)	Both	Total cancers	559.36(525.41 to 594.3)	611.64(461.41 to 778.63)	0.56(0.38 to 0.75)	<0.001
DALYs (Disability-Adjusted Life Years)	Age-standardized	Viet Nam	Both	Total cancers	319.57(240.19 to 412.98)	293.63(222.17 to 377.75)	-0.22(-0.29 to -0.15)	<0.001
DALYs (Disability-Adjusted Life Years)	Age-standardized	Yemen	Both	Total cancers	262.51(125.24 to 422.39)	280.39(141.27 to 455.91)	0.16(-0.18 to 0.5)	0.362
DALYs (Disability-Adjusted Life Years)	Age-standardized	Zambia	Both	Total cancers	566.57(458.65 to 698.09)	573.92(356.3 to 898.24)	0.03(-0.27 to 0.32)	0.864
DALYs (Disability-Adjusted Life Years)	Age-standardized	Zimbabwe	Both	Total cancers	299.97(216.78 to 386.55)	587.22(441.27 to 765.89)	2.79(1.95 to 3.64)	<0.001

Supplementary Table 4: AAPC Values of Cancer Disease Burden Among Adolescents Aged 10-24 in the World and 204 Countries/Regions (Death)

Measure	Age	Location	Sex	Cause	1990 year Rate (95%UI)	2021 year Rate (95%UI)	AAPC(95%CI)	P Value
Deaths	Age-standardized	Global	Both	Total cancers	6.8(6.11 to 7.34)	5.01(4.51 to 5.45)	-1.07(-1.12 to -1.02)	<0.001
Deaths	Age-standardized	Afghanistan	Both	Total cancers	10.9(6.53 to 15.54)	10.09(6.85 to 13.39)	-0.18(-0.28 to -0.08)	0.001
Deaths	Age-standardized	Albania	Both	Total cancers	7.41(6.33 to 8.59)	4(3.13 to 4.97)	-1.31(-1.84 to -0.78)	<0.001
Deaths	Age-standardized	Algeria	Both	Total cancers	5.73(4.52 to 7.15)	3.67(2.87 to 4.59)	-1.32(-1.41 to -1.23)	<0.001
Deaths	Age-standardized	American Samoa	Both	Total cancers	4.54(3.6 to 5.69)	5.49(4.19 to 6.94)	0.75(0.6 to 0.91)	<0.001
Deaths	Age-standardized	Andorra	Both	Total cancers	5.49(4 to 7.43)	3.55(2.59 to 4.73)	-1.33(-1.47 to -1.19)	<0.001
Deaths	Age-standardized	Angola	Both	Total cancers	4.02(2.89 to 5.39)	3.63(2.42 to 5.13)	-0.2(-0.59 to 0.19)	0.319
Deaths	Age-standardized	Antigua and Barbuda	Both	Total cancers	4.79(4.3 to 5.33)	4.5(4.1 to 4.95)	-0.02(-0.85 to 0.82)	0.958
Deaths	Age-standardized	Argentina	Both	Total cancers	7.37(6.74 to 7.97)	5.45(5.02 to 5.93)	-1.02(-1.29 to -0.74)	<0.001
Deaths	Age-standardized	Armenia	Both	Total cancers	7.29(6.78 to 7.87)	5.01(4.28 to 5.79)	-0.91(-1.79 to -0.02)	0.045
Deaths	Age-standardized	Australia	Both	Total cancers	5.24(4.91 to 5.58)	2.83(2.58 to 3.11)	-2.03(-2.48 to -1.57)	<0.001
Deaths	Age-standardized	Austria	Both	Total cancers	4.83(4.5 to 5.17)	2.91(2.67 to 3.18)	-1.47(-2.01 to -0.92)	<0.001
Deaths	Age-standardized	Azerbaijan	Both	Total cancers	8.88(7.48 to 10.7)	6.8(5.21 to 8.69)	-1.22(-1.59 to -0.86)	<0.001
Deaths	Age-standardized	Bahamas	Both	Total cancers	5.95(5.38 to 6.56)	6.06(4.82 to 7.5)	0.22(-0.32 to 0.76)	0.419
Deaths	Age-standardized	Bahrain	Both	Total cancers	5.95(5.01 to 7)	3.57(2.91 to 4.42)	-1.19(-1.49 to -0.9)	<0.001
Deaths	Age-standardized	Bangladesh	Both	Total cancers	6.82(5.22 to 8.61)	5.86(4.26 to 7.88)	-0.35(-0.58 to -0.13)	0.002
Deaths	Age-standardized	Barbados	Both	Total cancers	8.59(7.9 to 9.33)	6.24(4.78 to 8.09)	-0.61(-0.85 to -0.37)	<0.001
Deaths	Age-standardized	Belarus	Both	Total cancers	5.58(5.12 to 6.04)	3.64(3.05 to 4.3)	-1.76(-2.35 to -1.18)	<0.001
Deaths	Age-standardized	Belgium	Both	Total cancers	5.66(5.25 to 6.08)	3.3(3 to 3.63)	-2.14(-2.66 to -1.62)	<0.001
Deaths	Age-standardized	Belize	Both	Total cancers	4.48(3.94 to 5.14)	3.78(3.36 to 4.23)	0.32(-0.34 to 1)	0.342
Deaths	Age-standardized	Benin	Both	Total cancers	3.28(2.43 to 4.49)	3.1(2.13 to 4.32)	-0.23(-0.35 to -0.11)	<0.001
Deaths	Age-standardized	Bermuda	Both	Total cancers	6.5(5.79 to 7.33)	4.31(3.59 to 5.29)	-1.32(-1.52 to -1.11)	<0.001

Deaths	Age-standardized	Bhutan	Both	Total cancers	4.84(2.82 to 6.75)	4.48(2.93 to 6.46)	-0.14(-0.52 to 0.24)	0.47
Deaths	Age-standardized	Bolivia (Plurinational State of)	Both	Total cancers	10.26(8.2 to 12.49)	7.94(5.61 to 11.05)	-0.82(-0.86 to -0.78)	<0.001
Deaths	Age-standardized	Bosnia and Herzegovina	Both	Total cancers	5.44(4.68 to 6.32)	4.27(3.46 to 5.16)	-1.11(-1.66 to -0.55)	<0.001
Deaths	Age-standardized	Botswana	Both	Total cancers	4.15(2.87 to 5.54)	3.77(2.63 to 5.05)	-0.29(-0.8 to 0.21)	0.251
Deaths	Age-standardized	Brazil	Both	Total cancers	5.89(5.57 to 6.18)	5.59(5.27 to 5.88)	-0.13(-0.27 to 0.02)	0.081
Deaths	Age-standardized	Brunei Darussalam	Both	Total cancers	9.46(7.54 to 11.52)	4.66(3.83 to 5.61)	-1.92(-2.08 to -1.76)	<0.001
Deaths	Age-standardized	Bulgaria	Both	Total cancers	7.78(7.05 to 8.52)	5.67(4.8 to 6.41)	-0.96(-2.13 to 0.24)	0.116
Deaths	Age-standardized	Burkina Faso	Both	Total cancers	3.38(2.4 to 4.93)	3.56(2.41 to 4.93)	0.42(0.15 to 0.69)	0.002
Deaths	Age-standardized	Burundi	Both	Total cancers	8.7(6.68 to 11.07)	6.59(4.83 to 8.83)	-1.02(-1.2 to -0.84)	<0.001
Deaths	Age-standardized	Cabo Verde	Both	Total cancers	3.8(2.87 to 4.89)	4.59(3.5 to 5.87)	0.5(0.23 to 0.76)	<0.001
Deaths	Age-standardized	Cambodia	Both	Total cancers	8.15(6.04 to 10.64)	6.74(4.57 to 9.65)	-0.74(-0.99 to -0.5)	<0.001
Deaths	Age-standardized	Cameroon	Both	Total cancers	3.67(2.81 to 4.76)	3.79(2.47 to 5.32)	0.03(-0.13 to 0.18)	0.731
Deaths	Age-standardized	Canada	Both	Total cancers	4.63(4.38 to 4.91)	3.14(2.86 to 3.47)	-1.25(-1.36 to -1.15)	<0.001
Deaths	Age-standardized	Central African Republic	Both	Total cancers	4.39(3.43 to 5.48)	4.08(2.99 to 5.32)	-0.3(-0.5 to -0.11)	0.003
Deaths	Age-standardized	Chad	Both	Total cancers	2.73(1.94 to 3.84)	3.46(2.45 to 4.85)	0.99(0.81 to 1.18)	<0.001
Deaths	Age-standardized	Chile	Both	Total cancers	6.05(5.59 to 6.53)	4.38(4.02 to 4.75)	-0.87(-0.97 to -0.77)	<0.001
Deaths	Age-standardized	China	Both	Total cancers	9.04(7.66 to 10.27)	5.3(4.35 to 6.32)	-1.88(-2.24 to -1.51)	<0.001
Deaths	Age-standardized	Colombia	Both	Total cancers	8.3(7.73 to 8.87)	6.74(5.72 to 7.81)	-0.74(-1.04 to -0.45)	<0.001
Deaths	Age-standardized	Comoros	Both	Total cancers	7.38(2.89 to 10.38)	7.23(5.16 to 9.64)	-0.47(-1.93 to 1)	0.527
Deaths	Age-standardized	Congo	Both	Total cancers	3.79(2.77 to 4.92)	3.83(2.89 to 4.9)	0.32(-0.26 to 0.9)	0.283
Deaths	Age-standardized	Cook Islands	Both	Total cancers	2.63(2.02 to 3.43)	1.98(1.5 to 2.65)	-0.87(-0.94 to -0.79)	<0.001
Deaths	Age-standardized	Costa Rica	Both	Total cancers	6.24(5.78 to 6.75)	7.17(6.44 to 7.89)	0.39(0.07 to 0.7)	0.016
Deaths	Age-standardized	Coted'Ivoire	Both	Total cancers	2.86(2.1 to 3.85)	2.98(2.01 to 4.16)	0.08(-0.29 to 0.46)	0.665
Deaths	Age-standardized	Croatia	Both	Total cancers	5.39(4.88 to 5.97)	3.59(3.12 to 4.05)	-0.94(-2.16 to 0.3)	0.136
Deaths	Age-standardized	Cuba	Both	Total cancers	7.61(7.06 to 8.21)	5.03(4.36 to 5.73)	-1.17(-1.47 to -0.86)	<0.001
Deaths	Age-standardized	Cyprus	Both	Total cancers	3.72(3.04 to 4.56)	2.29(1.82 to 2.83)	-1.12(-1.7 to -0.53)	<0.001

Deaths	Age-standardized	Czechia	Both	Total cancers	6.03(5.57 to 6.51)	3.51(3.02 to 4.06)	-1.15(-1.54 to -0.76)	<0.001
Deaths	Age-standardized	Democratic People's Republic of Korea	Both	Total cancers	7.51(5.1 to 10.54)	6.26(4.71 to 8.57)	-0.66(-0.75 to -0.58)	<0.001
Deaths	Age-standardized	Democratic Republic of the Congo	Both	Total cancers	3.32(2.49 to 4.28)	3.08(2.26 to 4.13)	-0.29(-0.5 to -0.08)	0.007
Deaths	Age-standardized	Denmark	Both	Total cancers	4.29(3.94 to 4.65)	2.47(2.23 to 2.73)	-2.14(-2.79 to -1.49)	<0.001
Deaths	Age-standardized	Djibouti	Both	Total cancers	4.98(3.45 to 6.87)	5.46(3.47 to 8.12)	0.55(0.04 to 1.05)	0.033
Deaths	Age-standardized	Dominica	Both	Total cancers	6.43(5.43 to 7.66)	7.72(5.92 to 9.66)	0.8(0.62 to 0.97)	<0.001
Deaths	Age-standardized	Dominican Republic	Both	Total cancers	5.23(4.39 to 6.21)	5.29(4.15 to 6.61)	0.43(0.14 to 0.72)	0.004
Deaths	Age-standardized	Ecuador	Both	Total cancers	7.33(6.62 to 8.03)	7.39(6.14 to 8.78)	0.62(0.17 to 1.06)	0.006
Deaths	Age-standardized	Egypt	Both	Total cancers	5.84(4.85 to 7.21)	5.84(4.64 to 7.27)	0.35(0.09 to 0.6)	0.007
Deaths	Age-standardized	El Salvador	Both	Total cancers	7.09(6.17 to 8.34)	6.83(5.16 to 8.74)	0.06(-0.32 to 0.45)	0.744
Deaths	Age-standardized	Equatorial Guinea	Both	Total cancers	3.88(2.83 to 5.15)	3.38(1.88 to 6.12)	-0.44(-0.83 to -0.05)	0.026
Deaths	Age-standardized	Eritrea	Both	Total cancers	6.74(5.23 to 8.6)	6.87(4.96 to 9.09)	0.02(-0.08 to 0.13)	0.649
Deaths	Age-standardized	Estonia	Both	Total cancers	6.72(6.14 to 7.37)	4.37(3.8 to 4.96)	-1.88(-2.65 to -1.1)	<0.001
Deaths	Age-standardized	Eswatini	Both	Total cancers	4.43(3.44 to 5.56)	6.05(3.63 to 8.66)	1.33(0.92 to 1.75)	<0.001
Deaths	Age-standardized	Ethiopia	Both	Total cancers	12.77(9.49 to 16.17)	7.72(5.96 to 9.62)	-1.87(-2.09 to -1.64)	<0.001
Deaths	Age-standardized	Fiji	Both	Total cancers	6.62(5.11 to 8.3)	6.21(4.46 to 8.36)	-0.03(-0.21 to 0.15)	0.748
Deaths	Age-standardized	Finland	Both	Total cancers	4.29(3.97 to 4.63)	3.28(2.94 to 3.65)	-0.81(-1.05 to -0.58)	<0.001
Deaths	Age-standardized	France	Both	Total cancers	5.36(5.06 to 5.67)	3.11(2.87 to 3.39)	-1.83(-1.92 to -1.73)	<0.001
Deaths	Age-standardized	Gabon	Both	Total cancers	3.56(2.76 to 4.45)	3.45(2.25 to 4.91)	-0.12(-0.34 to 0.09)	0.263
Deaths	Age-standardized	Gambia	Both	Total cancers	3.38(2.34 to 4.64)	4.08(2.66 to 5.7)	0.26(-0.06 to 0.57)	0.11
Deaths	Age-standardized	Georgia	Both	Total cancers	8.23(7.2 to 9.18)	7.12(6.09 to 8.09)	-0.54(-1.37 to 0.29)	0.2
Deaths	Age-standardized	Germany	Both	Total cancers	5.36(5.04 to 5.7)	2.95(2.72 to 3.21)	-2.07(-2.27 to -1.86)	<0.001
Deaths	Age-standardized	Ghana	Both	Total cancers	5.61(3.48 to 7.81)	3.71(2.65 to 5.04)	-1.7(-2.29 to -1.1)	<0.001
Deaths	Age-standardized	Greece	Both	Total cancers	6.31(5.97 to 6.7)	4.23(3.91 to 4.56)	-1.28(-1.6 to -0.95)	<0.001
Deaths	Age-standardized	Greenland	Both	Total cancers	8.46(6.34 to 12.51)	2.7(1.95 to 3.72)	-3.74(-3.96 to -3.52)	<0.001
Deaths	Age-standardized	Grenada	Both	Total cancers	8.53(7.47 to 9.72)	6.33(5.4 to 7.4)	-0.54(-0.94 to -0.14)	0.008

Deaths	Age-standardized	Guam	Both	Total cancers	4.55(3.81 to 5.34)	3.27(2.59 to 3.99)	-1.18(-3.8 to 1.52)	0.39
Deaths	Age-standardized	Guatemala	Both	Total cancers	6.5(6.1 to 6.96)	6.1(5.26 to 7.02)	-0.1(-0.5 to 0.3)	0.614
Deaths	Age-standardized	Guinea	Both	Total cancers	4.05(2.97 to 5.4)	4.1(2.91 to 5.8)	0.07(-0.11 to 0.26)	0.449
Deaths	Age-standardized	Guinea-Bissau	Both	Total cancers	5.31(3.82 to 7.03)	4.78(3.46 to 6.83)	-0.28(-0.37 to -0.2)	<0.001
Deaths	Age-standardized	Guyana	Both	Total cancers	5.13(3.98 to 6.07)	5.25(3.97 to 6.78)	0.59(0 to 1.19)	0.048
Deaths	Age-standardized	Haiti	Both	Total cancers	10.1(6.77 to 13.52)	8.39(5.43 to 11.78)	-0.39(-0.74 to -0.04)	0.028
Deaths	Age-standardized	Honduras	Both	Total cancers	7.06(5.79 to 8.6)	4.27(2.66 to 6.47)	-1.78(-1.99 to -1.57)	<0.001
Deaths	Age-standardized	Hungary	Both	Total cancers	6.31(5.89 to 6.79)	3.66(3.2 to 4.13)	-1.55(-1.86 to -1.23)	<0.001
Deaths	Age-standardized	Iceland	Both	Total cancers	5.33(4.83 to 5.85)	3.82(3.38 to 4.3)	-1.35(-1.82 to -0.88)	<0.001
Deaths	Age-standardized	India	Both	Total cancers	5.2(4.37 to 6.01)	4.1(3.5 to 4.82)	-0.76(-0.91 to -0.6)	<0.001
Deaths	Age-standardized	Indonesia	Both	Total cancers	6.35(5.11 to 7.62)	5.87(4.66 to 7.48)	-0.26(-0.31 to -0.2)	<0.001
Deaths	Age-standardized	Iran (Islamic Republic of)	Both	Total cancers	7.59(5.8 to 8.9)	6.23(4.9 to 7.21)	-0.45(-0.77 to -0.12)	0.008
Deaths	Age-standardized	Iraq	Both	Total cancers	7.3(5.63 to 9.47)	5.36(4.03 to 7.06)	-1.14(-1.31 to -0.97)	<0.001
Deaths	Age-standardized	Ireland	Both	Total cancers	5.05(4.67 to 5.47)	2.25(2.02 to 2.5)	-2.51(-2.92 to -2.09)	<0.001
Deaths	Age-standardized	Israel	Both	Total cancers	5.06(4.67 to 5.51)	3.01(2.74 to 3.32)	-1.6(-2.01 to -1.2)	<0.001
Deaths	Age-standardized	Italy	Both	Total cancers	6.63(6.45 to 6.79)	3.52(3.37 to 3.67)	-2.28(-2.77 to -1.8)	<0.001
Deaths	Age-standardized	Jamaica	Both	Total cancers	4.62(4.03 to 5.16)	4.06(3.18 to 5.2)	-1.09(-1.72 to -0.47)	0.001
Deaths	Age-standardized	Japan	Both	Total cancers	4.48(4.38 to 4.59)	2.86(2.8 to 2.93)	-1.4(-1.63 to -1.17)	<0.001
Deaths	Age-standardized	Jordan	Both	Total cancers	6.58(5.33 to 8.16)	4.14(3.21 to 5.25)	-1.61(-1.79 to -1.43)	<0.001
Deaths	Age-standardized	Kazakhstan	Both	Total cancers	9.67(8.97 to 10.43)	4.03(3.54 to 4.59)	-2.82(-3.1 to -2.54)	<0.001
Deaths	Age-standardized	Kenya	Both	Total cancers	4.02(3.09 to 4.96)	4.57(3.53 to 5.87)	0.7(0.31 to 1.1)	<0.001
Deaths	Age-standardized	Kiribati	Both	Total cancers	5.47(4.17 to 6.96)	5.64(3.89 to 7.9)	0.11(0.07 to 0.15)	<0.001
Deaths	Age-standardized	Kuwait	Both	Total cancers	4.96(4.46 to 5.52)	2.55(2.12 to 3.07)	-2.42(-2.75 to -2.09)	<0.001
Deaths	Age-standardized	Kyrgyzstan	Both	Total cancers	6.69(5.82 to 7.62)	4.55(3.83 to 5.42)	-0.89(-1.26 to -0.53)	<0.001
Deaths	Age-standardized	Lao People's Democratic Republic	Both	Total cancers	8.12(5.45 to 11.15)	6.71(4.61 to 9.44)	-0.62(-0.7 to -0.53)	<0.001
Deaths	Age-standardized	Latvia	Both	Total cancers	6.29(5.79 to 6.82)	3.83(3.39 to 4.24)	-1.07(-1.69 to -0.45)	0.001

Deaths	Age-standardized	Lebanon	Both	Total cancers	6.17(4.64 to 8.21)	3.94(3.11 to 5.03)	-1.31(-1.48 to -1.13)	<0.001
Deaths	Age-standardized	Lesotho	Both	Total cancers	3.13(2.31 to 4.05)	5.72(4.01 to 7.61)	2.63(2.04 to 3.22)	<0.001
Deaths	Age-standardized	Liberia	Both	Total cancers	3.48(2.49 to 4.71)	4.02(2.7 to 5.54)	0.64(0.19 to 1.09)	0.006
Deaths	Age-standardized	Libya	Both	Total cancers	6.81(5.4 to 8.47)	6.86(5.12 to 8.99)	0.39(-0.09 to 0.88)	0.11
Deaths	Age-standardized	Lithuania	Both	Total cancers	5.31(4.87 to 5.79)	3.81(3.38 to 4.23)	-0.88(-1.17 to -0.59)	<0.001
Deaths	Age-standardized	Luxembourg	Both	Total cancers	6.97(6.41 to 7.58)	2.55(2.25 to 2.89)	-4.07(-4.34 to -3.81)	<0.001
Deaths	Age-standardized	Madagascar	Both	Total cancers	6.59(5.28 to 8.18)	6.34(4.49 to 8.32)	-0.1(-0.19 to -0.02)	0.02
Deaths	Age-standardized	Malawi	Both	Total cancers	7.38(5.52 to 9.58)	7.48(5.03 to 10.76)	0.13(0.04 to 0.21)	0.003
Deaths	Age-standardized	Malaysia	Both	Total cancers	6.4(4.97 to 7.93)	4.71(3.72 to 5.8)	-1.21(-1.64 to -0.79)	<0.001
Deaths	Age-standardized	Maldives	Both	Total cancers	4.8(3.37 to 6.31)	2.56(2.01 to 3.26)	-1.82(-2.08 to -1.56)	<0.001
Deaths	Age-standardized	Mali	Both	Total cancers	4.2(3.15 to 5.4)	3.64(2.65 to 4.79)	-0.35(-0.47 to -0.23)	<0.001
Deaths	Age-standardized	Malta	Both	Total cancers	4.79(4.23 to 5.29)	4.73(3.97 to 5.52)	-0.91(-1.4 to -0.43)	0.001
Deaths	Age-standardized	Marshall Islands	Both	Total cancers	5.09(3.97 to 6.58)	5.47(3.88 to 7.39)	0.36(0.07 to 0.66)	0.015
Deaths	Age-standardized	Mauritania	Both	Total cancers	3.82(2.65 to 5.4)	3.01(2.16 to 4.16)	-0.77(-0.98 to -0.56)	<0.001
Deaths	Age-standardized	Mauritius	Both	Total cancers	4.12(3.82 to 4.42)	3.83(3.4 to 4.16)	-0.5(-0.93 to -0.07)	0.023
Deaths	Age-standardized	Mexico	Both	Total cancers	7.01(6.8 to 7.25)	7.17(6.63 to 7.73)	0.26(0.15 to 0.37)	<0.001
Deaths	Age-standardized	Micronesia (Federated States of)	Both	Total cancers	5.46(4.23 to 6.99)	5.29(3.69 to 7.28)	-0.14(-0.19 to -0.09)	<0.001
Deaths	Age-standardized	Monaco	Both	Total cancers	6.89(5.24 to 8.7)	6.74(5.22 to 8.67)	-0.18(-0.29 to -0.07)	0.001
Deaths	Age-standardized	Mongolia	Both	Total cancers	7.94(6.6 to 9.76)	6.75(5.77 to 7.87)	-0.62(-1.04 to -0.19)	0.006
Deaths	Age-standardized	Montenegro	Both	Total cancers	6.1(5.1 to 7.17)	4.78(3.84 to 5.81)	-0.46(-1.01 to 0.11)	0.113
Deaths	Age-standardized	Morocco	Both	Total cancers	3.22(2.49 to 4.07)	2.49(1.88 to 3.57)	-0.87(-0.94 to -0.8)	<0.001
Deaths	Age-standardized	Mozambique	Both	Total cancers	5.18(3.83 to 6.98)	5.93(3.87 to 8.43)	0.58(0.29 to 0.87)	<0.001
Deaths	Age-standardized	Myanmar	Both	Total cancers	9.41(6.17 to 12.94)	6.48(4.78 to 8.87)	-1.39(-1.74 to -1.04)	<0.001
Deaths	Age-standardized	Namibia	Both	Total cancers	4.32(3.29 to 5.54)	4.48(3.13 to 6.77)	0.19(0.05 to 0.32)	0.007
Deaths	Age-standardized	Nauru	Both	Total cancers	6.19(4.69 to 7.94)	6.6(4.62 to 8.91)	0.22(0.17 to 0.26)	<0.001
Deaths	Age-standardized	Nepal	Both	Total cancers	5.5(3.97 to 7.17)	4.65(3.32 to 6.49)	-0.46(-0.59 to -0.32)	<0.001

Deaths	Age-standardized	Netherlands	Both	Total cancers	4.39(4.12 to 4.67)	2.59(2.37 to 2.83)	-1.9(-2.25 to -1.55)	<0.001
Deaths	Age-standardized	New Zealand	Both	Total cancers	5.95(5.51 to 6.44)	3.28(2.99 to 3.57)	-1.84(-2.18 to -1.5)	<0.001
Deaths	Age-standardized	Nicaragua	Both	Total cancers	5.63(4.84 to 6.71)	4.31(3.44 to 5.3)	-1.16(-1.69 to -0.63)	<0.001
Deaths	Age-standardized	Niger	Both	Total cancers	3.25(2.27 to 4.63)	2.72(1.75 to 4.04)	-0.51(-0.68 to -0.33)	<0.001
Deaths	Age-standardized	Nigeria	Both	Total cancers	4.04(2.55 to 5.67)	3.53(2.02 to 5.44)	-0.47(-0.67 to -0.26)	<0.001
Deaths	Age-standardized	Niue	Both	Total cancers	4.2(3.03 to 5.88)	10.2(8.04 to 12.85)	2.95(1.83 to 4.08)	<0.001
Deaths	Age-standardized	North Macedonia	Both	Total cancers	7.35(6.42 to 8.46)	5.5(4.31 to 6.83)	-1(-1.38 to -0.62)	<0.001
Deaths	Age-standardized	Northern Mariana Islands	Both	Total cancers	3.49(2.58 to 4.69)	3.23(2.56 to 3.98)	-0.26(-0.77 to 0.26)	0.319
Deaths	Age-standardized	Norway	Both	Total cancers	4.36(4.19 to 4.53)	3.44(3.26 to 3.62)	-1.37(-2.03 to -0.7)	<0.001
Deaths	Age-standardized	Oman	Both	Total cancers	3.39(2.44 to 4.66)	2.16(1.71 to 2.71)	-1.08(-2.08 to -0.08)	0.035
Deaths	Age-standardized	Pakistan	Both	Total cancers	7.28(5.82 to 8.74)	9.34(7.53 to 11.58)	0.79(0.63 to 0.94)	<0.001
Deaths	Age-standardized	Palau	Both	Total cancers	5.98(4.05 to 8.54)	7.13(5.34 to 9.46)	0.52(0.39 to 0.65)	<0.001
Deaths	Age-standardized	Palestine	Both	Total cancers	7.27(5.45 to 9.69)	5.3(4.32 to 6.64)	-0.83(-1.17 to -0.49)	<0.001
Deaths	Age-standardized	Panama	Both	Total cancers	7.02(6.46 to 7.66)	6.51(5.4 to 7.68)	-0.22(-0.86 to 0.42)	0.497
Deaths	Age-standardized	Papua New Guinea	Both	Total cancers	4.09(1.56 to 6.23)	4.42(2.58 to 6.4)	0.18(0.04 to 0.32)	0.016
Deaths	Age-standardized	Paraguay	Both	Total cancers	5.02(4.2 to 6.06)	5.42(4.23 to 6.99)	0.3(0.07 to 0.53)	0.01
Deaths	Age-standardized	Peru	Both	Total cancers	8.78(7.44 to 10.35)	6.55(5.08 to 8.42)	-0.73(-0.86 to -0.59)	<0.001
Deaths	Age-standardized	Philippines	Both	Total cancers	6.94(6.08 to 7.75)	5.98(5.09 to 6.88)	-0.34(-0.58 to -0.1)	0.005
Deaths	Age-standardized	Poland	Both	Total cancers	6.6(6.36 to 6.78)	3.64(3.39 to 3.83)	-1.71(-1.8 to -1.61)	<0.001
Deaths	Age-standardized	Portugal	Both	Total cancers	8.07(7.52 to 8.69)	3.16(2.83 to 3.52)	-3.44(-3.71 to -3.17)	<0.001
Deaths	Age-standardized	Puerto Rico	Both	Total cancers	5.91(5.37 to 6.48)	3.95(3.26 to 4.69)	-1.36(-1.83 to -0.88)	<0.001
Deaths	Age-standardized	Qatar	Both	Total cancers	4.86(3.74 to 6.29)	2.88(2.17 to 3.83)	-1.29(-1.63 to -0.94)	<0.001
Deaths	Age-standardized	Republic of Korea	Both	Total cancers	8.28(6.4 to 9.47)	2.78(2.17 to 3.26)	-3.9(-4.27 to -3.53)	<0.001
Deaths	Age-standardized	Republic of Moldova	Both	Total cancers	7.76(7.27 to 8.29)	3.39(2.99 to 3.84)	-2.13(-2.65 to -1.61)	<0.001
Deaths	Age-standardized	Romania	Both	Total cancers	7.2(6.64 to 7.79)	4.24(3.76 to 4.76)	-1.45(-1.79 to -1.12)	<0.001
Deaths	Age-standardized	Russian Federation	Both	Total cancers	7.86(7.64 to 8.07)	3.71(3.49 to 3.91)	-2.68(-3.13 to -2.23)	<0.001

Deaths	Age-standardized	Rwanda	Both	Total cancers	10.42(8.41 to 12.65)	6.32(4.42 to 8.79)	-1.99(-2.65 to -1.33)	<0.001
Deaths	Age-standardized	Saint Kitts and Nevis	Both	Total cancers	5.68(5.15 to 6.24)	5.15(4.26 to 6.27)	-0.65(-1.08 to -0.22)	0.003
Deaths	Age-standardized	Saint Lucia	Both	Total cancers	6.15(5.64 to 6.67)	5.85(4.82 to 7.04)	-0.28(-0.64 to 0.07)	0.119
Deaths	Age-standardized	Saint Vincent and the Grenadines	Both	Total cancers	7.57(6.78 to 8.48)	8.54(7.37 to 9.91)	0.46(0.07 to 0.85)	0.021
Deaths	Age-standardized	Samoa	Both	Total cancers	5.55(3.88 to 7.7)	5.32(3.77 to 7.38)	-0.07(-0.15 to 0.01)	0.11
Deaths	Age-standardized	San Marino	Both	Total cancers	6.38(5.25 to 7.82)	4.15(3.04 to 5.57)	-1.43(-1.58 to -1.28)	<0.001
Deaths	Age-standardized	Sao Tome and Principe	Both	Total cancers	2.51(1.31 to 4.13)	2.88(1.88 to 4.4)	0.65(-0.09 to 1.41)	0.087
Deaths	Age-standardized	Saudi Arabia	Both	Total cancers	3.86(2.81 to 5.19)	3.32(2.54 to 4.32)	-0.37(-0.5 to -0.25)	<0.001
Deaths	Age-standardized	Senegal	Both	Total cancers	3.47(2.58 to 4.67)	3.36(2.4 to 4.65)	0.16(-0.02 to 0.35)	0.084
Deaths	Age-standardized	Serbia	Both	Total cancers	6.7(5.65 to 7.84)	2.94(2.41 to 3.59)	-2.64(-3.04 to -2.23)	<0.001
Deaths	Age-standardized	Seychelles	Both	Total cancers	6.94(5.89 to 8.15)	2.93(2.44 to 3.46)	-0.51(-1.11 to 0.08)	0.089
Deaths	Age-standardized	Sierra Leone	Both	Total cancers	2.77(1.8 to 4.18)	3.3(2.18 to 4.72)	0.83(0.5 to 1.16)	<0.001
Deaths	Age-standardized	Singapore	Both	Total cancers	5.09(4.7 to 5.51)	4.31(3.9 to 4.75)	-1.01(-1.69 to -0.34)	0.003
Deaths	Age-standardized	Slovakia	Both	Total cancers	5.4(4.74 to 6.22)	3.8(3.13 to 4.51)	-1.1(-1.78 to -0.42)	0.002
Deaths	Age-standardized	Slovenia	Both	Total cancers	4.82(4.48 to 5.2)	2.63(2.27 to 3.1)	-2.09(-2.52 to -1.66)	<0.001
Deaths	Age-standardized	Solomon Islands	Both	Total cancers	4.1(1.75 to 6.29)	4.9(3.27 to 6.83)	0.65(0.51 to 0.79)	<0.001
Deaths	Age-standardized	Somalia	Both	Total cancers	6.29(4.48 to 8.25)	6.45(4.65 to 8.62)	0.29(0.05 to 0.52)	0.017
Deaths	Age-standardized	South Africa	Both	Total cancers	3.55(3.09 to 4.06)	3.4(2.94 to 3.94)	-0.38(-0.89 to 0.12)	0.135
Deaths	Age-standardized	South Sudan	Both	Total cancers	5.05(3.52 to 6.95)	6.97(4.84 to 9.64)	0.73(0.2 to 1.27)	0.007
Deaths	Age-standardized	Spain	Both	Total cancers	6.57(6.16 to 6.97)	3.41(3.12 to 3.73)	-2.39(-2.61 to -2.16)	<0.001
Deaths	Age-standardized	Sri Lanka	Both	Total cancers	7.06(5.65 to 8.53)	3.9(2.77 to 5.24)	-2.32(-2.88 to -1.75)	<0.001
Deaths	Age-standardized	Sudan	Both	Total cancers	6.67(4.45 to 9.16)	6.36(4.05 to 9.27)	-0.03(-0.18 to 0.12)	0.662
Deaths	Age-standardized	Suriname	Both	Total cancers	6.76(4.3 to 8.03)	6.79(5.55 to 8.27)	-0.32(-0.92 to 0.29)	0.309
Deaths	Age-standardized	Sweden	Both	Total cancers	4.27(3.97 to 4.6)	2.87(2.58 to 3.17)	-0.89(-1.06 to -0.71)	<0.001
Deaths	Age-standardized	Switzerland	Both	Total cancers	5.52(5.11 to 5.98)	2.54(2.3 to 2.82)	-2.52(-2.8 to -2.24)	<0.001
Deaths	Age-standardized	Syrian Arab Republic	Both	Total cancers	7.12(5.53 to 8.93)	5(3.77 to 6.57)	-1.36(-1.76 to -0.95)	<0.001

Deaths	Age-standardized	Taiwan (Province of China)	Both	Total cancers	5.8(5.47 to 6.16)	4.24(3.82 to 4.65)	-1.15(-1.6 to -0.7)	<0.001
Deaths	Age-standardized	Tajikistan	Both	Total cancers	8.05(6.82 to 9.47)	6.54(5.11 to 8.28)	-1.12(-1.82 to -0.41)	0.002
Deaths	Age-standardized	Thailand	Both	Total cancers	6.91(5.63 to 8.25)	6.19(4.77 to 8.02)	-0.47(-0.85 to -0.09)	0.015
Deaths	Age-standardized	Timor-Leste	Both	Total cancers	5.39(3.75 to 7.4)	5.18(3.78 to 6.99)	-0.05(-0.85 to 0.76)	0.909
Deaths	Age-standardized	Togo	Both	Total cancers	3.08(2.33 to 4.07)	3.22(2.16 to 4.53)	0.14(-0.03 to 0.3)	0.113
Deaths	Age-standardized	Tokelau	Both	Total cancers	4.65(3.26 to 6.14)	11.34(8.51 to 14.92)	3.28(1.91 to 4.68)	<0.001
Deaths	Age-standardized	Tonga	Both	Total cancers	4(3.08 to 5.19)	4.87(3.55 to 6.91)	0.86(0.6 to 1.13)	<0.001
Deaths	Age-standardized	Trinidad and Tobago	Both	Total cancers	6.28(5.76 to 6.77)	5.88(4.61 to 7.39)	0.28(-0.01 to 0.56)	0.057
Deaths	Age-standardized	Tunisia	Both	Total cancers	4.71(3.89 to 5.72)	3.69(2.71 to 4.99)	-0.73(-0.89 to -0.57)	<0.001
Deaths	Age-standardized	Turkey	Both	Total cancers	11.16(8.69 to 13.92)	5.91(4.7 to 7.27)	-2.05(-2.29 to -1.8)	<0.001
Deaths	Age-standardized	Turkmenistan	Both	Total cancers	7.45(7 to 7.96)	9.24(7.44 to 11.55)	0.73(0.37 to 1.09)	<0.001
Deaths	Age-standardized	Tuvalu	Both	Total cancers	5.06(3.9 to 6.45)	4.41(3.26 to 5.7)	-0.4(-0.46 to -0.34)	<0.001
Deaths	Age-standardized	Uganda	Both	Total cancers	6.18(4.28 to 8.57)	8.37(5.77 to 11.28)	0.71(0.32 to 1.1)	<0.001
Deaths	Age-standardized	Ukraine	Both	Total cancers	9.34(8.51 to 10.23)	5.05(3.89 to 6.29)	-2.39(-3.03 to -1.74)	<0.001
Deaths	Age-standardized	United Arab Emirates	Both	Total cancers	7.52(5.64 to 9.99)	5.16(3.98 to 6.46)	-1.17(-2.51 to 0.19)	0.093
Deaths	Age-standardized	United Kingdom	Both	Total cancers	5.04(4.97 to 5.11)	3.27(3.17 to 3.36)	-1.42(-1.55 to -1.28)	<0.001
Deaths	Age-standardized	United Republic of Tanzania	Both	Total cancers	6.92(5.4 to 8.75)	6.51(4.53 to 8.63)	-0.19(-0.29 to -0.08)	0.001
Deaths	Age-standardized	United States Virgin Islands	Both	Total cancers	6.63(5.28 to 8.28)	6.5(4.55 to 8.71)	0.7(0.37 to 1.03)	<0.001
Deaths	Age-standardized	United States of America	Both	Total cancers	4.79(4.71 to 4.87)	3.12(2.99 to 3.26)	-1.46(-1.55 to -1.38)	<0.001
Deaths	Age-standardized	Uruguay	Both	Total cancers	6.56(5.99 to 7.16)	6.45(5.81 to 7.15)	-0.26(-0.66 to 0.14)	0.207
Deaths	Age-standardized	Uzbekistan	Both	Total cancers	7.08(6.34 to 7.91)	5.95(5.04 to 6.98)	-0.41(-0.76 to -0.05)	0.025
Deaths	Age-standardized	Vanuatu	Both	Total cancers	3.65(2.37 to 5.23)	4.33(3.07 to 5.8)	0.59(0.21 to 0.97)	0.002
Deaths	Age-standardized	Venezuela (Bolivarian Republic of)	Both	Total cancers	7.65(7.19 to 8.13)	8.4(6.31 to 10.7)	0.51(0.32 to 0.71)	<0.001
Deaths	Age-standardized	Viet Nam	Both	Total cancers	4.41(3.31 to 5.7)	4.02(3.04 to 5.17)	-0.25(-0.32 to -0.18)	<0.001
Deaths	Age-standardized	Yemen	Both	Total cancers	3.53(1.65 to 5.71)	3.8(1.89 to 6.22)	0.18(-0.17 to 0.54)	0.312
Deaths	Age-standardized	Zambia	Both	Total cancers	7.84(6.34 to 9.65)	7.95(4.91 to 12.46)	0.02(-0.27 to 0.31)	0.891

Deaths	Age-standardized	Zimbabwe	Both	Total cancers	4.15(3 to 5.33)	8.12(6.1 to 10.62)	2.8(1.96 to 3.64)	<0.001

Supplementary Table 5: Distribution of Different Tumors Across Various Measures in 1990 and 2021(Number)

Measure	Type of Cancer	1990 Year Number (95%UI)	2021 Year Number (95%UI)
Prevalence	Total cancers	891522.37(845124.39 to 930115.75)	1349913.04(1252506.95 to 1442998.32)
	Bladder cancer	5789.38(4651.4 to 6340.01)	6499.91(5776.2 to 7360.2)
	Brain and central nervous system cancer	70073.7(59780.91 to 78008.05)	129045.2(110304.35 to 150927.73)
	Breast cancer	27483.38(25375.38 to 30063.43)	66460.77(56782.41 to 76440.61)
	Cervical cancer	55336.53(49879.36 to 61600.92)	74064.88(65314.6 to 85848.04)
	Colon and rectum cancer	26778.7(23199.38 to 29495.47)	33125.26(29888.68 to 36449.19)
	Esophageal cancer	1418.51(1213.96 to 1575.36)	1217.61(1080.18 to 1406.5)
	Eye cancer	13038.97(8124.91 to 19737.09)	18924.89(11084.93 to 29346.92)
	Gallbladder and biliary tract cancer	180.57(135.73 to 202.49)	248.89(191.53 to 292.33)
	Hodgkin lymphoma	71443.6(63074.23 to 76856.55)	79268.28(63394.79 to 94078.81)
	Kidney cancer	16341.66(15485.77 to 17323.93)	25657.11(23880.74 to 27557.5)
	Larynx cancer	954.02(768.42 to 1050.94)	1110.45(963.7 to 1307.62)
	Leukemia	88734.31(75871.43 to 99510.22)	118157.7(93427.67 to 133101.48)
	Lip and oral cavity cancer	9127.83(8595.06 to 9743.99)	17899.63(14279.23 to 21531.4)
	Liver cancer	4604.49(4080.26 to 5324.2)	4245.16(3809.39 to 4776.3)
	Malignant neoplasm of bone and articular cartilage	91281.52(78952.66 to 107210.2)	133895.21(113220.93 to 160864.74)
	Malignant skin melanoma	22624.32(21448.8 to 23621.36)	32737.09(28748.45 to 35753.44)
	Mesothelioma	191.42(165.66 to 234.88)	267.51(227.92 to 303.52)

	Multiple myeloma	190.39(160.78 to 244.51)	581.18(374.94 to 733.71)
	Nasopharynx cancer	16731.8(15004.57 to 18572.22)	18251.27(16011.25 to 20739.57)
	Neuroblastoma and other peripheral nervous cell tumors	2873.86(2030.17 to 3878.82)	5353.26(3874.98 to 6935.79)
	Non-Hodgkin lymphoma	101673.61(93265.4 to 109805.34)	159001.64(144469.7 to 177976.12)
	Non-melanoma skin cancer	4935.41(2848.87 to 7973.63)	4496.57(2839.14 to 6865.15)
	Other malignant neoplasms	57620.49(47978.26 to 63990.88)	81357.1(68437.16 to 91930.82)
	Other neoplasms	7719759.67(4926949.34 to 11874805.3)	8549991.34(5697081.22 to 12667006.07)
	Other pharynx cancer	176.41(153.31 to 193.58)	436.14(358.98 to 510.48)
	Ovarian cancer	46675.87(35896.25 to 57721.46)	77407.83(62846.77 to 89228.07)
	Pancreatic cancer	617.63(558.02 to 673.28)	664.59(600.74 to 747.77)
	Prostate cancer	1410.9(1148.76 to 1602.19)	2780.21(2198.13 to 3201.3)
	Soft tissue and other extraosseous sarcomas	37294.31(30904.99 to 44989.61)	42889.95(36170.6 to 55380.77)
	Stomach cancer	10982.26(9443.16 to 12023.31)	7450.06(6271.06 to 8224.61)
	Testicular cancer	53481.46(49998.25 to 57278.64)	110852.12(102501.1 to 121324.29)
	Thyroid cancer	41980.24(36477.36 to 48566.21)	85310.81(69781.25 to 109872.31)
	Tracheal, bronchus, and lung cancer	5096.31(4642.13 to 5597.21)	4576.44(4218.25 to 4932.28)
	Uterine cancer	4378.53(2866.94 to 5321.9)	5678.34(4336.01 to 6659.16)
Incidence	Total cancers	187467(174955.6 to 197932.54)	235249.05(217211.16 to 251070.1)

Bladder cancer	785.95(627.49 to 860.41)	840.53(747.27 to 959.83)
Brain and central nervous system cancer	18559.13(15290.1 to 21282.04)	25873.55(21932.68 to 30758.72)
Breast cancer	3433.6(3166.46 to 3765.61)	8152.23(6920.06 to 9429.52)
Cervical cancer	8689.84(7774.59 to 9742.95)	10896.45(9563.67 to 12771.67)
Colon and rectum cancer	5372.34(4635.58 to 5940.84)	5685.05(5134.12 to 6248.6)
Esophageal cancer	583.37(500.62 to 648.44)	453.29(403.76 to 529.91)
Eye cancer	1461.39(912.63 to 2206.81)	2123.61(1244.87 to 3279.56)
Gallbladder and biliary tract cancer	152.31(110.22 to 173.65)	169.58(129.61 to 204.18)
Hodgkin lymphoma	11980.94(9929.91 to 13260.08)	12797.77(9722.46 to 15540.7)
Kidney cancer	2267.24(2145.49 to 2403.07)	3370.94(3136.17 to 3623.51)
Larynx cancer	205.27(162.79 to 227.58)	220.76(188.62 to 262.75)
Leukemia	39131.53(32838.43 to 43732.06)	34684.74(27664.82 to 38775.94)
Lip and oral cavity cancer	1955.14(1831.04 to 2101.27)	3559.11(2789.08 to 4332.81)
Liver cancer	2736.63(2418.6 to 3173.19)	2409.02(2152.04 to 2726.47)
Malignant neoplasm of bone and articular cartilage	12950.62(11198.55 to 15235.33)	18927.57(16009.84 to 22749.3)
Malignant skin melanoma	2821.68(2624.86 to 2974.94)	3938.09(3370.1 to 4352.17)
Mesothelioma	85.06(73.48 to 103.56)	114.12(97.59 to 129.5)
Multiple myeloma	88.46(71.8 to 118.67)	201(131.93 to 249.04)
Nasopharynx cancer	3923.45(3524.03 to 4346.4)	3457.45(3018.21 to 3910.78)
Neuroblastoma and other peripheral nervous cell tumors	425.2(321.94 to 558.56)	812.42(623.91 to 1020.86)
Non-Hodgkin lymphoma	15488.42(14226.18 to 16931.63)	21511.72(19487.01 to 24114.87)

	Non-melanoma skin cancer	8447.12(5574 to 12141.87)	11161.83(7614.97 to 15775.55)
	Other malignant neoplasms	11417.67(9344.73 to 12906.99)	13629.65(11431.96 to 15271.14)
	Other neoplasms	4707195.26(3263066.04 to 7796361.25)	5108055.68(3591448.97 to 8190587.58)
	Other pharynx cancer	167.73(138.2 to 191.61)	302.75(233.4 to 370.41)
	Ovarian cancer	6878.04(5252.96 to 8544.59)	11087.49(8975.46 to 12812.8)
	Pancreatic cancer	434.21(390.4 to 475.24)	426.85(382.73 to 485.52)
	Prostate cancer	162.83(130.89 to 185.44)	306.82(242.48 to 353.16)
	Soft tissue and other extraosseous sarcomas	6505.9(5392.53 to 7832.71)	7440.87(6265.59 to 9626.61)
	Stomach cancer	4707.56(4047.92 to 5165.32)	2765.18(2347.42 to 3027.96)
	Testicular cancer	7633.05(7223.49 to 8070.47)	15560.29(14685.07 to 16732.38)
	Thyroid cancer	4739.64(4118.63 to 5470.26)	9472.35(7742.6 to 12198.75)
	Tracheal, bronchus, and lung cancer	2725.36(2477.12 to 2998.37)	2209.6(2040.37 to 2383.43)
	Uterine cancer	550.31(355.21 to 673.23)	686.34(523.65 to 807.31)
Death	Total cancers	105662.59(95297.08 to 113611.51)	94856.02(85970.2 to 102769.63)
	Bladder cancer	245.15(194.13 to 271.05)	192.5(167.24 to 224.29)
	Brain and central nervous system cancer	11838.84(9321.34 to 13896.12)	12199.58(10059.68 to 14590.21)
	Breast cancer	1187.98(1067.5 to 1327.87)	2234.48(1822.04 to 2715.15)
	Cervical cancer	2464.61(2174.08 to 2845.77)	2476.79(2144.44 to 2955.55)
	Colon and rectum cancer	3535.41(3001.81 to 3935.95)	2591.81(2298.86 to 2917.62)
	Esophageal cancer	573.44(494.37 to 637.96)	411.02(364.32 to 487.24)
	Eye cancer	117.88(87.85 to 138.6)	165.75(111.26 to 219.5)
	Gallbladder and biliary tract cancer	138.78(97.65 to 160.44)	131.65(97.75 to 161.49)

Hodgkin lymphoma	4740.44(3202.13 to 5640.64)	4100.06(2677.05 to 5359.73)
Kidney cancer	720.92(678.3 to 769.82)	722.52(658.82 to 786.41)
Larynx cancer	156.3(121.32 to 174.51)	141.3(117.11 to 172.58)
Leukemia	35374.44(29370.95 to 39906.19)	25688.54(20441.25 to 28782.07)
Lip and oral cavity cancer	928.25(861.45 to 1007.22)	1421.42(1096.66 to 1743.29)
Liver cancer	2780.68(2463.42 to 3215.56)	2299.49(2038.38 to 2628.81)
Malignant neoplasm of bone and articular cartilage	8352.89(7121.79 to 10055.18)	10696(9107.49 to 13103.59)
Malignant skin melanoma	482.02(394.95 to 535.41)	506.56(361.17 to 645.07)
Mesothelioma	80.22(69.06 to 97.72)	105.21(89.63 to 119.86)
Multiple myeloma	75.18(60.04 to 103)	142.16(95.14 to 174.65)
Nasopharynx cancer	2646.39(2367.92 to 2931.84)	1649.08(1413.22 to 1909.51)
Neuroblastoma and other peripheral nervous cell tumors	234.73(192.46 to 286.45)	411.76(343.39 to 469.82)
Non-Hodgkin lymphoma	8580.44(7636.55 to 9671.89)	8676.76(7615.23 to 10035.26)
Non-melanoma skin cancer	151.08(129.67 to 180.45)	151.07(120.3 to 167.72)
Other malignant neoplasms	7517.66(5898.89 to 8698.65)	6650.42(5509.05 to 7533.52)
Other neoplasms	239.86(168.09 to 387.22)	309.98(243.38 to 452.87)
Other pharynx cancer	129.25(105.34 to 149.37)	197.02(149.03 to 243.71)
Ovarian cancer	1132.11(850.65 to 1408.88)	1573.32(1268.67 to 1819.27)
Pancreatic cancer	428.02(384.17 to 469.1)	404.68(361.32 to 460.97)
Prostate cancer	58.16(41.02 to 69.88)	60.56(44.93 to 71.6)
Soft tissue and other extraosseous sarcomas	2784.76(2239.81 to 3405.66)	2750.14(2304.13 to 3647.09)
Stomach cancer	3595.35(3102.28 to 3952.98)	1739.05(1487.52 to 1886.92)

	Testicular cancer	1207.35(1119.65 to 1296.89)	1458.41(1362.41 to 1561.92)
	Thyroid cancer	559.28(488.16 to 655.01)	722.43(570.06 to 948.69)
	Tracheal, bronchus, and lung cancer	2688.73(2441.53 to 2955.63)	2062.63(1899.4 to 2227.29)
	Uterine cancer	155.9(88.77 to 198.16)	121.87(92.71 to 147.01)
DALYs (Disability-Adjusted Life Years)	Total cancers	7689222.5(6935362.88 to 8261872.84)	6918657.72(6254353.93 to 7480202.6)
	Bladder cancer	17573.04(13888.15 to 19441.33)	13906.7(12111.65 to 16213.72)
	Brain and central nervous system cancer	872580.55(686899.71 to 1023052.43)	900848.39(742453.49 to 1081650.44)
	Breast cancer	84488.4(76010.99 to 94403.19)	160007.74(130815.53 to 194085.33)
	Cervical cancer	174198.7(153202.71 to 200791.38)	175945.12(152096.45 to 209720.23)
	Colon and rectum cancer	248466.53(210799.31 to 276661.88)	182608.86(162128.22 to 205212.45)
	Esophageal cancer	39000.73(33616.8 to 43392.11)	27950.51(24764.96 to 33173.63)
	Eye cancer	9333.04(6993.58 to 10975.93)	13054.82(8700.15 to 17503.68)
	Gallbladder and biliary tract cancer	9427.34(6633.17 to 10895.41)	8945.34(6643.48 to 10975.45)
	Hodgkin lymphoma	345248.68(233545.27 to 410949.25)	300278.46(195822.04 to 392818.68)
	Kidney cancer	53385.81(50266.52 to 56804.6)	53972.16(49031.06 to 58891.43)
	Larynx cancer	10670.56(8288.52 to 11917.8)	9662.34(8007.36 to 11811.86)
	Leukemia	2592112.7(2151716.29 to 2925640.64)	1879886.2(1494484.09 to 2110457.32)

Lip and oral cavity cancer	65354.95(60697.23 to 70931.38)	100095.14(77151.32 to 122770.78)
Liver cancer	198037.42(175466.14 to 228699.1)	163380.66(144834.04 to 187190.05)
Malignant neoplasm of bone and articular cartilage	614608.75(524197.79 to 739574.91)	786164.9(666998.85 to 965705.7)
Malignant skin melanoma	34697.3(28670.83 to 38420.9)	36913.89(26534.89 to 46840.31)
Mesothelioma	5457.08(4696.56 to 6647.58)	7154.96(6098.06 to 8152.98)
Multiple myeloma	5115.2(4083.55 to 7009.65)	9682.85(6481.98 to 11880.39)
Nasopharynx cancer	191256.29(171321.19 to 211643.88)	120014.56(102776.67 to 139432.94)
Neuroblastoma and other peripheral nervous cell tumors	17575.75(14414.29 to 21488.11)	30737.03(25507.71 to 35108.61)
Non-Hodgkin lymphoma	629440.07(558655.98 to 708227.89)	638332.13(562512.42 to 737541.5)
Non-melanoma skin cancer	10413.99(8936.25 to 12345.16)	10369.88(8324.29 to 11504.98)
Other malignant neoplasms	552341.49(431876.88 to 639876.12)	488421.97(404082.85 to 553467.45)
Other neoplasms	27885.21(21196.12 to 39851.11)	31750.99(25416.57 to 41857.76)
Other pharynx cancer	8789.22(7162.83 to 10158.08)	13409.84(10147.76 to 16582.23)
Ovarian cancer	82306.42(61990.59 to 102049.06)	115074.77(92424.79 to 133110.32)
Pancreatic cancer	29779.09(26728.47 to 32655.88)	28087.2(25072.02 to 31988.44)
Prostate cancer	4078.03(2902.84 to 4938.86)	4348.51(3226.66 to 5154.91)

	Soft tissue and other extraosseous sarcomas	205571.92(165404.13 to 250414.16)	202534.59(169461.57 to 268256.91)
	Stomach cancer	249871.21(215393.97 to 274243.58)	120759.97(103252.42 to 131095.64)
	Testicular cancer	87099(80738.55 to 93465.67)	107980.89(100337.33 to 115571.86)
	Thyroid cancer	42172.72(36830.23 to 49221.86)	55811.7(44160.94 to 74031.71)
	Tracheal, bronchus, and lung cancer	187927.28(170512.96 to 206786.75)	143696.2(132394.18 to 155251.72)
	Uterine cancer	10843.21(6158.31 to 13745.9)	8619.43(6538.57 to 10405.64)

Supplementary Table 6: Results of Decompositional Analysis for the 10-24 Age Group

location	sex	cause	measure	Aging	Population	Epidemiological change	Overll difference
Global	Both	Total cancers	Deaths	-215.87 (-0.2%)	20185.56 (19.1%)	-30776.25 (-29.13%)	-10806.57(-10.23%)
Global	Both	Total cancers	DALYs (Disability-Adjusted Life Years)	-12212.57 (-0.16%)	1470406.15 (19.12%)	-2228758.35 (-28.99%)	-770564.78(-10.02%)
Global	Both	Total cancers	Prevalence	-3503.51 (-0.39%)	219928.31 (24.67%)	241965.87 (27.14%)	458390.67(51.42%)
Global	Both	Total cancers	Incidence	-624.28 (-0.33%)	41857.81 (22.33%)	6548.51 (3.49%)	47782.05(25.49%)
High SDI	Both	Total cancers	Deaths	-21.23 (-0.2%)	-447.78 (-4.2%)	-4113.95 (-38.61%)	-4582.96(-43.01%)
High SDI	Both	Total cancers	DALYs (Disability-Adjusted Life Years)	-1353.46 (-0.17%)	-32989.56 (-4.23%)	-295104.86 (-37.8%)	-329447.87(-42.2%)
High SDI	Both	Total cancers	Prevalence	-705.88 (-0.28%)	-13907.3 (-5.49%)	23686.19 (9.36%)	9073.01(3.58%)
High SDI	Both	Total cancers	Incidence	-86.4 (-0.21%)	-2154.26 (-5.27%)	407.1 (1%)	-1833.56(-4.49%)
High-middle SDI	Both	Total cancers	Deaths	-195.03 (-0.82%)	-3791.81 (-15.97%)	-9065.04 (-38.17%)	-13051.88(-54.96%)
High-middle SDI	Both	Total cancers	DALYs (Disability-Adjusted Life Years)	-11281.5 (-0.65%)	-276144.14 (-16.03%)	-651737.36 (-37.83%)	-939163(-54.52%)
High-middle SDI	Both	Total cancers	Prevalence	-3677.19 (-1.69%)	-54728.88 (-25.15%)	93973.63 (43.18%)	35567.56(16.34%)
High-middle SDI	Both	Total cancers	Incidence	-612.25	-9395.25	4760.29	-5247.22(-12.01%)

				(-1.4%)	(-21.5%)	(10.89%)	
Middle SDI	Both	Total cancers	Deaths	-170.31 (-0.42%)	247.86 (0.61%)	-11931.07 (-29.28%)	-11853.52(-29.09%)
Middle SDI	Both	Total cancers	DALYs (Disability-Adjusted Life Years)	-9559.71 (-0.32%)	18011.86 (0.61%)	-860798.55 (-29.12%)	-852346.39(-28.83%)
Middle SDI	Both	Total cancers	Prevalence	-2024.54 (-0.84%)	2234.88 (0.93%)	146155.03 (60.64%)	146365.37(60.73%)
Middle SDI	Both	Total cancers	Incidence	-410.39 (-0.68%)	464.71 (0.77%)	9850.57 (16.32%)	9904.9(16.41%)
Low-middle SDI	Both	Total cancers	Deaths	371.85 (1.78%)	10629.39 (50.91%)	-2234.74 (-10.7%)	8766.49(41.99%)
Low-middle SDI	Both	Total cancers	DALYs (Disability-Adjusted Life Years)	19607.41 (1.29%)	774209.16 (50.79%)	-164344.85 (-10.78%)	629471.71(41.29%)
Low-middle SDI	Both	Total cancers	Prevalence	4172.55 (3.42%)	80647.64 (66.07%)	74430.51 (60.98%)	159250.69(130.47%)
Low-middle SDI	Both	Total cancers	Incidence	875.72 (2.99%)	17176.93 (58.63%)	7443.37 (25.4%)	25496.02(87.02%)
Low SDI	Both	Total cancers	Deaths	97.89 (1.03%)	12187.87 (127.66%)	-2357.57 (-24.69%)	9928.18(103.99%)
Low SDI	Both	Total cancers	DALYs (Disability-Adjusted Life Years)	5311.54 (0.76%)	890365.89 (127.46%)	-173821.71 (-24.88%)	721855.72(103.34%)
Low SDI	Both	Total cancers	Prevalence	957.03 (1.68%)	86641.56 (152.39%)	20331.31 (35.76%)	107929.9(189.83%)
Low SDI	Both	Total cancers	Incidence	202.65 (1.55%)	18368.71 (140.58%)	877.94 (6.72%)	19449.3(148.85%)
Sub-Saharan Africa	Both	Total cancers	Deaths	46.43	10994.77	-1604.76	9436.44(112.78%)

				(0.55%)	(131.41%)	(-19.18%)	
Sub-Saharan Africa	Both	Total cancers	DALYs (Disability-Adjusted Life Years)	2577.11 (0.42%)	802895.42 (131.34%)	-117422.25 (-19.21%)	688050.27(112.55%)
Sub-Saharan Africa	Both	Total cancers	Prevalence	445.67 (0.81%)	84802.18 (153.44%)	19048.33 (34.47%)	104296.17(188.72%)
Sub-Saharan Africa	Both	Total cancers	Incidence	98.74 (0.82%)	17357.47 (143.36%)	1183.97 (9.78%)	18640.18(153.95%)
Southeast Asia, East Asia, and Oceania	Both	Total cancers	Deaths	-622.87 (-1.42%)	-7251.94 (-16.51%)	-13192.18 (-30.04%)	-21067(-47.98%)
Southeast Asia, East Asia, and Oceania	Both	Total cancers	DALYs (Disability-Adjusted Life Years)	-35208.47 (-1.11%)	-525799.74 (-16.56%)	-952157.49 (-29.99%)	-1513165.7(-47.66%)
Southeast Asia, East Asia, and Oceania	Both	Total cancers	Prevalence	-6912.06 (-2.81%)	-65984.59 (-26.83%)	156513.19 (63.64%)	83616.54(34%)
Southeast Asia, East Asia, and Oceania	Both	Total cancers	Incidence	-1431.32 (-2.28%)	-13513.41 (-21.51%)	9704.71 (15.45%)	-5240.02(-8.34%)
African Union	Both	Total cancers	Deaths	49.27 (0.46%)	12245.78 (113.55%)	-1816.56 (-16.84%)	10478.48(97.17%)
African Union	Both	Total cancers	DALYs (Disability-Adjusted Life Years)	2603.4 (0.33%)	895376 (113.44%)	-134305.13 (-17.02%)	763674.27(96.75%)
African Union	Both	Total cancers	Prevalence	499.71 (0.69%)	97812.37 (135.52%)	30123.72 (41.74%)	128435.81(177.95%)
African Union	Both	Total cancers	Incidence	109.96 (0.7%)	19796 (125.61%)	2394.43 (15.19%)	22300.39(141.5%)
South Asia	Both	Total cancers	Deaths	375.63 (2.02%)	10116.38 (54.45%)	-2662.83 (-14.33%)	7829.18(42.14%)
South Asia	Both	Total cancers	DALYs (Disability-Adjusted Life Years)	19622.55	736305.86	-193848.15	562080.25(41.47%)

				(1.45%)	(54.32%)	(-14.3%)	
South Asia	Both	Total cancers	Prevalence	4676.84 (4.25%)	79706.85 (72.45%)	71949.11 (65.4%)	156332.81(142.11%)
South Asia	Both	Total cancers	Incidence	905.3 (3.49%)	16509.45 (63.7%)	6822.23 (26.32%)	24236.98(93.52%)
Latin America and Caribbean	Both	Total cancers	Deaths	165.45 (1.94%)	1268.77 (14.89%)	-604.65 (-7.09%)	829.57(9.73%)
Latin America and Caribbean	Both	Total cancers	DALYs (Disability-Adjusted Life Years)	8624.84 (1.39%)	92335.31 (14.85%)	-43779.86 (-7.04%)	57180.29(9.2%)
Latin America and Caribbean	Both	Total cancers	Prevalence	2519.41 (4.32%)	11654.01 (19.99%)	37031.02 (63.51%)	51204.45(87.81%)
Latin America and Caribbean	Both	Total cancers	Incidence	657.81 (4.62%)	2571.91 (18.06%)	5089.28 (35.74%)	8319(58.43%)
WHO region	Both	Total cancers	Deaths	-223.29 (-0.21%)	20345.5 (19.34%)	-30706.5 (-29.2%)	-10584.28(-10.06%)
WHO region	Both	Total cancers	DALYs (Disability-Adjusted Life Years)	-12605.67 (-0.16%)	1482066.85 (19.36%)	-2223864.71 (-29.06%)	-754403.53(-9.86%)
WHO region	Both	Total cancers	Prevalence	-3627.26 (-0.41%)	221112.68 (24.99%)	241085.86 (27.25%)	458571.28(51.83%)
WHO region	Both	Total cancers	Incidence	-649.07 (-0.35%)	42128.82 (22.61%)	6482.22 (3.48%)	47961.97(25.74%)
Four World Regions	Both	Total cancers	Deaths	-217.1 (-0.21%)	20207.18 (19.15%)	-30749.4 (-29.14%)	-10759.33(-10.2%)
Four World Regions	Both	Total cancers	DALYs (Disability-Adjusted Life Years)	-12275.9 (-0.16%)	1471985.43 (19.17%)	-2226844.14 (-29%)	-767134.61(-9.99%)
Four World Regions	Both	Total cancers	Prevalence	-3524.48	220089.13	241566.44	458131.08(51.48%)

				(-0.4%)	(24.73%)	(27.14%)	
Four World Regions	Both	Total cancers	Incidence	-628.65 (-0.34%)	41895.03 (22.38%)	6522.18 (3.48%)	47788.56(25.53%)
Commonwealth	Both	Total cancers	Deaths	323.16 (1.32%)	15836.19 (64.62%)	-3659.93 (-14.93%)	12499.42(51.01%)
Commonwealth	Both	Total cancers	DALYs (Disability-Adjusted Life Years)	17114.95 (0.96%)	1154515.71 (64.53%)	-267082.29 (-14.93%)	904548.38(50.55%)
Commonwealth	Both	Total cancers	Prevalence	4925.45 (2.65%)	145472.28 (78.19%)	69699.75 (37.46%)	220097.48(118.3%)
Commonwealth	Both	Total cancers	Incidence	907.12 (2.32%)	28263.6 (72.32%)	5692.41 (14.57%)	34863.13(89.21%)
North Africa and Middle East	Both	Total cancers	Deaths	61.39 (0.81%)	3303.86 (43.61%)	-2135.52 (-28.19%)	1229.73(16.23%)
North Africa and Middle East	Both	Total cancers	DALYs (Disability-Adjusted Life Years)	2643.63 (0.48%)	242464.16 (43.6%)	-155052.92 (-27.88%)	90054.88(16.19%)
North Africa and Middle East	Both	Total cancers	Prevalence	1389.76 (2.63%)	35535.99 (67.17%)	47990.27 (90.71%)	84916.02(160.51%)
North Africa and Middle East	Both	Total cancers	Incidence	225.08 (1.92%)	6560.39 (56.04%)	4019.58 (34.34%)	10805.05(92.3%)
World Bank Income Levels	Both	Total cancers	Deaths	-216.31 (-0.2%)	20182.27 (19.12%)	-30759.6 (-29.14%)	-10793.65(-10.22%)
World Bank Income Levels	Both	Total cancers	DALYs (Disability-Adjusted Life Years)	-12235.46 (-0.16%)	1470165.63 (19.14%)	-2227557.53 (-28.99%)	-769627.36(-10.02%)
World Bank Income Levels	Both	Total cancers	Prevalence	-3511.12 (-0.39%)	219874.34 (24.69%)	241821.91 (27.15%)	458185.13(51.44%)
World Bank Income Levels	Both	Total cancers	Incidence	-625.9	41849.67	6545.54	47769.31(25.5%)

				(-0.33%)	(22.34%)	(3.49%)	
OECD Countries	Both	Total cancers	Deaths	-2.93 (-0.02%)	-711.91 (-4.28%)	-5416.95 (-32.54%)	-6131.79(-36.84%)
OECD Countries	Both	Total cancers	DALYs (Disability-Adjusted Life Years)	-322.53 (-0.03%)	-52348.72 (-4.29%)	-389042.9 (-31.91%)	-441714.15(-36.22%)
OECD Countries	Both	Total cancers	Prevalence	334.71 (0.1%)	-18142.54 (-5.51%)	46632.69 (14.16%)	28824.86(8.75%)
OECD Countries	Both	Total cancers	Incidence	100.16 (0.18%)	-2895.72 (-5.27%)	2802.19 (5.1%)	6.63(0.01%)
World Bank Regions	Both	Total cancers	Deaths	-216.76 (-0.21%)	20190.14 (19.13%)	-30750.03 (-29.14%)	-10776.64(-10.21%)
World Bank Regions	Both	Total cancers	DALYs (Disability-Adjusted Life Years)	-12259.54 (-0.16%)	1470738.2 (19.15%)	-2226877.84 (-29%)	-768399.18(-10.01%)
World Bank Regions	Both	Total cancers	Prevalence	-3518.29 (-0.4%)	219936.69 (24.7%)	241659.38 (27.14%)	458077.78(51.45%)
World Bank Regions	Both	Total cancers	Incidence	-627.38 (-0.34%)	41863.34 (22.36%)	6535.31 (3.49%)	47771.27(25.52%)
High-income	Both	Total cancers	Deaths	-57.67 (-0.51%)	-580.78 (-5.1%)	-4415.7 (-38.75%)	-5054.15(-44.35%)
High-income	Both	Total cancers	DALYs (Disability-Adjusted Life Years)	-3470 (-0.41%)	-42902.63 (-5.13%)	-316863.29 (-37.88%)	-363235.92(-43.42%)
High-income	Both	Total cancers	Prevalence	-2851.21 (-0.98%)	-19049.4 (-6.57%)	18448.63 (6.37%)	-3451.99(-1.19%)
High-income	Both	Total cancers	Incidence	-463.95 (-1.01%)	-2902.26 (-6.32%)	-529.34 (-1.15%)	-3895.55(-8.49%)
G20	Both	Total cancers	Deaths	-216.5	205.86	-25287.09	-25297.73(-34.74%)

				(-0.3%)	(0.28%)	(-34.72%)	
G20	Both	Total cancers	DALYs (Disability-Adjusted Life Years)	-12613.09 (-0.24%)	14977.47 (0.28%)	-1826248.3 (-34.52%)	-1823883.92(-34.48%)
G20	Both	Total cancers	Prevalence	-3706.42 (-0.56%)	2565.5 (0.39%)	175692.18 (26.51%)	174551.25(26.34%)
G20	Both	Total cancers	Incidence	-653.43 (-0.48%)	475 (0.35%)	4120.6 (3.01%)	3942.17(2.88%)
Central Europe, Eastern Europe, and Central Asia	Both	Total cancers	Deaths	-15.55 (-0.21%)	-1390.84 (-19.03%)	-2603.96 (-35.62%)	-4010.35(-54.86%)
Central Europe, Eastern Europe, and Central Asia	Both	Total cancers	DALYs (Disability-Adjusted Life Years)	-921.95 (-0.17%)	-101516.28 (-19.05%)	-189090.62 (-35.49%)	-291528.85(-54.71%)
Central Europe, Eastern Europe, and Central Asia	Both	Total cancers	Prevalence	-323.39 (-0.41%)	-19015.78 (-23.98%)	815.85 (1.03%)	-18523.33(-23.36%)
Central Europe, Eastern Europe, and Central Asia	Both	Total cancers	Incidence	-47.47 (-0.32%)	-3285.81 (-22.24%)	-1750.3 (-11.85%)	-5083.58(-34.41%)
Nordic Region	Both	Total cancers	Deaths	-2.85 (-1.36%)	3.43 (1.65%)	-63.98 (-30.68%)	-63.39(-30.39%)
Nordic Region	Both	Total cancers	DALYs (Disability-Adjusted Life Years)	-162.65 (-1.06%)	256.05 (1.66%)	-4488.7 (-29.17%)	-4395.3(-28.57%)
Nordic Region	Both	Total cancers	Prevalence	-181.23 (-2.79%)	145.07 (2.23%)	1984.41 (30.5%)	1948.26(29.94%)
Nordic Region	Both	Total cancers	Incidence	-25.52 (-2.77%)	19.49 (2.11%)	171.32 (18.57%)	165.29(17.92%)
Gulf Cooperation Council	Both	Total cancers	Deaths	12.79 (4.58%)	197.96 (70.9%)	-75.69 (-27.11%)	135.06(48.37%)
Gulf Cooperation Council	Both	Total cancers	DALYs (Disability-Adjusted Life Years)	742.34	14411.61	-5579.25	9574.7(46.87%)

				(3.63%)	(70.55%)	(-27.31%)	
Gulf Cooperation Council	Both	Total cancers	Prevalence	301.85 (11.12%)	3172.86 (116.86%)	3665.7 (135.02%)	7140.4(263%)
Gulf Cooperation Council	Both	Total cancers	Incidence	45.82 (9.13%)	500.76 (99.81%)	372.82 (74.31%)	919.41(183.26%)
Association of Southeast Asian Nations	Both	Total cancers	Deaths	150.98 (1.62%)	1368.49 (14.68%)	-1284.79 (-13.78%)	234.68(2.52%)
Association of Southeast Asian Nations	Both	Total cancers	DALYs (Disability-Adjusted Life Years)	8398.76 (1.24%)	99363.02 (14.65%)	-93213.07 (-13.75%)	14548.71(2.15%)
Association of Southeast Asian Nations	Both	Total cancers	Prevalence	1929.15 (3.3%)	11230.76 (19.23%)	28124.17 (48.17%)	41284.07(70.7%)
Association of Southeast Asian Nations	Both	Total cancers	Incidence	359.82 (2.61%)	2333.17 (16.92%)	2279.46 (16.53%)	4972.45(36.06%)
Organization of Islamic Cooperation	Both	Total cancers	Deaths	206.03 (0.99%)	13791.83 (66.43%)	-3349.01 (-16.13%)	10648.85(51.29%)
Organization of Islamic Cooperation	Both	Total cancers	DALYs (Disability-Adjusted Life Years)	10429.31 (0.69%)	1008068.09 (66.35%)	-244480.94 (-16.09%)	774016.46(50.95%)
Organization of Islamic Cooperation	Both	Total cancers	Prevalence	2863.94 (2.16%)	118333.07 (89.34%)	94424.82 (71.29%)	215621.83(162.79%)
Organization of Islamic Cooperation	Both	Total cancers	Incidence	553.21 (1.81%)	23802.74 (78.08%)	8565.18 (28.1%)	32921.13(107.99%)
European Union	Both	Total cancers	Deaths	-33.77 (-0.6%)	-1004.74 (-17.82%)	-2224.12 (-39.45%)	-3262.63(-57.87%)
European Union	Both	Total cancers	DALYs (Disability-Adjusted Life Years)	-2022.64 (-0.49%)	-74134.29 (-17.97%)	-158731.66 (-38.47%)	-234888.59(-56.93%)
European Union	Both	Total cancers	Prevalence	-1640.7	-31835.2	19098.85	-14377.06(-11.21%)

				(-1.28%)	(-24.83%)	(14.9%)	
European Union	Both	Total cancers	Incidence	-228.88 (-1.18%)	-4505.66 (-23.17%)	403.53 (2.08%)	-4331.01(-22.27%)
Sahel Region	Both	Total cancers	Deaths	-0.19 (-0.01%)	3877.16 (177.74%)	-455.45 (-20.88%)	3421.52(156.86%)
Sahel Region	Both	Total cancers	DALYs (Disability-Adjusted Life Years)	-6.74 (0%)	283975.19 (177.78%)	-33239.22 (-20.81%)	250729.23(156.97%)
Sahel Region	Both	Total cancers	Prevalence	-12.38 (-0.1%)	26375.94 (207.91%)	5263.78 (41.49%)	31627.33(249.3%)
Sahel Region	Both	Total cancers	Incidence	-2.98 (-0.1%)	5787.49 (193.47%)	349.16 (11.67%)	6133.67(205.04%)

Supplementary Table 7. BAPC projection model results to 2050

Location	Year	Sex	Measure	Cause	Number(95%CI)	Rate(95%CI)
Global	2050	Male	Incidence	Total cancers	102794.19 (0 to 222892.18)	8.93 (-0.43 to 18.28)
Global	2050	Female	Incidence	Total cancers	122109.21 (1318.86 to 250313.93)	11.43 (0.48 to 22.39)
Global	2050	Both	Incidence	Total cancers	224903.4 (1318.86 to 473206.11)	11.6 (0.06 to 24.48)
Global	2050	Male	Prevalence	Total cancers	669751.14 (0 to 1522980.07)	57.03 (-7.22 to 121.29)
Global	2050	Female	Prevalence	Total cancers	810956.5 (0 to 1712738.91)	75.04 (-0.63 to 150.71)
Global	2050	Both	Prevalence	Total cancers	1480707.64 (0 to 3235718.98)	76.46 (0 to 167.58)
Global	2050	Male	DALYs (Disability-Adjusted Life Years)	Total cancers	2137005.93 (0 to 4653458.08)	187.03 (-10.87 to 384.92)
Global	2050	Female	DALYs (Disability-Adjusted Life Years)	Total cancers	2284068.6 (0 to 4854223.12)	213.1 (-3.9 to 430.1)
Global	2050	Both	DALYs (Disability-Adjusted Life Years)	Total cancers	4421074.52 (0 to 9507681.2)	230.76 (0 to 498.2)
Global	2050	Male	Deaths	Total cancers	30319.4 (238.53 to 63140.53)	2.69 (0.02 to 5.37)
Global	2050	Female	Deaths	Total cancers	32570.01 (414.88 to 66812.56)	3.07 (0.11 to 6.04)
Global	2050	Both	Deaths	Total cancers	62889.41 (653.42 to 129953.09)	3.27 (0.03 to 6.78)

Abbreviations: BAPC, bayesian age-period-cohort; CI, confidence interval.
